# Obituary: Dr. Hyun-Sul Lim’s (1952-2018) life as an epidemiologist, occupational and environmental medicine researcher, and family physician

**DOI:** 10.4178/epih.e2018033

**Published:** 2018-07-14

**Authors:** Sun Huh

**Affiliations:** Department of Parasitology and Institute of Medical Education, Hallym University College of Medicine, Chuncheon, Korea

Dr. Hyun-Sul Lim passed away on June 21, 2018 (Figures 1 and 2). He had suffered from gallbladder cancer, which was detected in June 2017. He was buried in Catholic Yongin Park Cemetery on June 23, 2018. He left his wife, one son, and one daughter.

I vividly remember a scene from 37 years ago, in March 1981, when I visited Chunseong-gun Health Center in Chuncheon, Korea, where he was the director. At that time, the medical college I attended, Seoul National University College of Medicine, had implemented a curriculum entitled “Community Medicine Practice.” When I and other friends visited Chunseong-gun Health Center, he welcomed us kindly and gave us guidelines for approaching members of the community. Three of us, including me, moved to Dong-myeon Subsidiary Health Center. We stayed there for 5 days and sought a research theme. We eventually finished our work successfully, and the results were presented before him and other friends. He encouraged us and emphasized the importance of community health. Although that meeting with him was short, I had the chance to meet him frequently when I worked at Seoul National University College of Medicine as a research and education assistant from March 1986 to February 1988. After moving to Hallym University in March 1988, I remained in touch with him. Although Dr. Lim was 4 years senior than me as an alumnus, we maintained a close relationship that originated from our experiences in Chuncheon in 1981.

Dr. Lim graduated from Seoul National University College of Medicine in 1978, and received his MPH (Master of Public Health) in 1981 from Seoul National University. His thesis advisor was Dr. Joung Soon Kim (1935- ). He received a PhD in preventive medicine in 1986 from the same university. His PhD thesis advisor was Dr. Dork Ro Yun (1933-2009).

In an interview with Dr. Sue Kyung Park, Professor of Seoul National University, on May 7, 2018 he stated that he decided to work as a medical scientist conducting basic research because of his experience in prison from April to August 1974 related with the Mincheonghakryeon (National Democratic Youth Student Federation) incident. When he was in prison, he pleaded that he would work for the Korean people if he was released. He was released without any sentence, and then developed an interest in preventive medicine [1].

He worked as the Director of the Chunseong-gun Health Center for 2 years. After that, he worked as a research assistant at the Seoul National University Graduate School of Public Health. His supervisor was Dr. Joung Soon Kim, a leading researcher in the field of epidemiology in Korea who worked as a professor from 1966 to 2001. When Dr. Kim studied leptospirosis epidemiology, she recommended Dr. Lim to be trained as a family medicine resident. He agreed with her, and therefore underwent resident training from March 1986 to February 1989 at the Seoul National University Hospital. During his training period, he continued to study the epidemiology of environmental diseases. For example, in 1988, he participated in the investigation of a case with cadmium intoxication. The direct cause of death in that case was cerebral hemorrhage due to hypertension, but exposure to cadmium may have been a triggering factor [2]. In 1988, there was a case of pneumoconiosis in a woman who had lived for 8 years in Sangbong-dong, Seoul near the Mangwoo briquette plant of Gangwon Industrial Co. He participated in an epidemiological survey of residents near the plant. This was the first case of pollution-origin disease to be recognized after a legal dispute [3].

He was recruited as a tenure-track faculty member in the Department of Preventive Medicine, Dongguk University in March 1990, where he worked for 27 years until his retirement in August 2017. Throughout that period, he continued to work on epidemiological studies. His representative achievements are as follows: the analysis of a case of carbon disulfide poisoning from labor at the Wonjin Viscose Rayon Company in 1991 [4]; the investigation of a case of noise-induced hearing loss in Maehyang-ri, Hwaseong, Gyeonggi-do, which resulted in a new law regarding such damage [5]; investigation of a case of glass fiber exposure in Gojan-dong, Incheon [6]; analysis of a case of ulceroglandular tularemia [7]; investigation of a case of green tobacco sickness [8]; and a study of the various health impacts of Agent Orange exposure in Korean Vietnam veterans [9].

Furthermore, his studies on bacterial dysentery [10], cholera [11], and brucellosis [12] were landmarks of the epidemiology of those infectious diseases in Korea. In the field of occupational medicine, he worked on burns caused by hydrogen fluoride [13], including a hydrogen fluoride spill accident in a manufacturing plant located at the fourth complex of the Gumi National Industrial Complex in Gumi City [14], and investigated farmers’ and fishers’ diseases. As for environmental diseases, he investigated pneumoconiosis in workers exposed to fumes containing manganese [15] and dioxin-contaminated soil at the Waegwan US Army Camp [16], and conducted an epidemiological survey of residents near a nuclear plant and analyzed epidemiological data of the victims of a subway fire in Daegu.

It is possible to trace his published articles by searching KoreaMed (https://koreamed.org) with the search term “Lim, Hyun-Sul” [FAU] and PubMed (https://pubmed.gov/) with the search term Lim, Hyun-Sul [Author - Full]. Some articles are found in both databases. Figure 3 presents his research topics through a word cloud of his article titles and abstracts from both databases (Supplementary Materials 1 and 2). The word cloud shows that he devoted himself to workers’ health and epidemiological surveys.

In addition to his scholarly achievements, he devoted himself to a variety of academic societies, serving as President of the Korean Society of Epidemiology from July 2004 to June 2006, President of the Korean Society for Rural Medicine and Community Health from January 2007 to February 2009, Chairman of the Board of Directors of the Korean Society for Preventive Medicine from December 2011 to November 2013, and a lifetime member of the National Academy of Medicine of Korea since 2004. His devotion as a leader of those societies propelled the advancement of those societies’ activities. He also worked as Dean of his medical college from March 2011 to February 2014.

In April 19, 2018, he received a proud alumnus award at the 28th Alumni Day of the Seoul National University College of Medicine in the auditorium of the college building. His academic achievements as an epidemiologist and devotion to scholarly societies were highly appreciated. This is shown by the co-author and keyword networks of articles published in *Journal of Preventive Medicine and Public Health* [17]. He was the top-ranking author according to the network indices by individual and structural effects, such as both the centrality measure and the structural holes measure. This means that he showed the highest level of productivity across a variety of topics.

He was a very unique specialist in Korea, as he was board-qualified in 3 specializations: preventive medicine, family medicine, and industrial and occupational medicine. This combination is very rare in Korea. His knowledge and skills as a specialist in 3 fields enabled him to achieve top-notch achievements in epidemiology and occupational and environmental medicine. In the May 7, 2018 interview, he emphasized the importance of cultivating the basic ability to execute epidemiologic surveys or investigations of occupational diseases as follows [9]: “First, read books of a variety of fields; second, have many experiences; and third, make a hypothesis immediately at the beginning of the survey.” He also recommended that members of the Korean Society of Epidemiology should work towards the advancement of epidemiology in Korea as follows: “Society members should discuss a variety of social problems and publish issues relating to them. In particular, epidemiological hot topics should be dealt with at society meetings or conferences. Such activities will help ease social tension. For scientific soundness, collect primary data. Although large-scale data are required, having high-quality data is more important.” His message was simple and lucid. This is invaluable advice for junior and future scholars.

I close this obituary by expressing my sincere condolences to his family. All society members miss him. He will be remembered as a warm and excellent scholar and teacher in the history of the Korean Society of Epidemiology and Dongguk University College of Medicine.

## Figures and Tables

**Figure 1.**
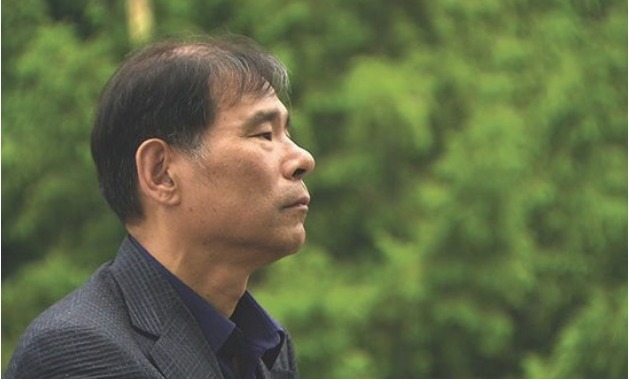
Picture of Dr. Hyun-Sul Lim in 2015 provided by his family.

**Figure 2.**
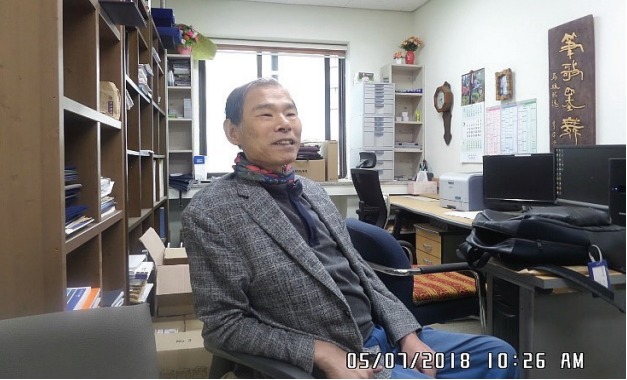
Picture of Dr. Hyun-Sul Lim in May 7, 2018 in his room at Dongguk University College of Medicine, photographed by Ms. Young Ju Choi, the Korean Society of Epidemiology.

**Figure 3.**
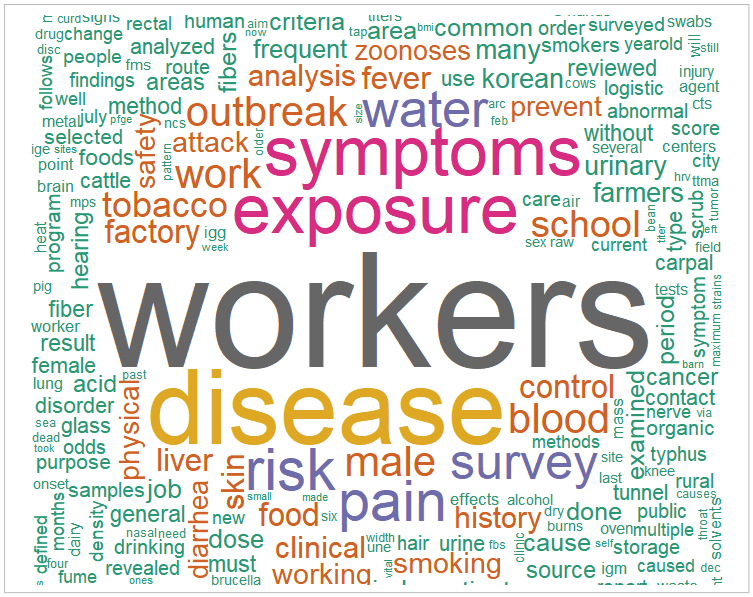
Word cloud presentation of Dr. Hyun-Sul Lim’s published articles based on their topics.

## SUPPLEMENTARY MATERIALS

Supplementary Material 1: Metadata of articles of Dr. Hyun-Sul Lim searchable from KoreaMed (https://koreamed.org) and PubMed (https://pubmed.gov).

Supplementary Material 2: Core topic words and frequency of Dr. Hyun-Sul Lim’s articles listed in KoreaMed (https://koreamed.org) and PubMed (https://pubmed.gov). The word cloud was based on this supplement.
