# Supplementary material for: Obituary: Dr. Hyun-Sul Lim’s (1952-2018) life as an epidemiologist, occupational and environmental medicine researcher, and family physician
Source: Epidemiol Health. 2018 Jul 14;40:e2018033. doi: 10.4178/epih.e2018033 (PMC6186862; doi:10.4178/epih.e2018033)
Supplement: Supplementary file 1 [file epih-40-e2018033-supplementary.pdf]

KoreaMed aritcles (168)

1: J Agric Med Community Health. 2018 Mar;43(1):9-17. Korean.

Status and Awareness of Excessive Heat Exposure among Agricultural Workers.

OBJECTIVES: This study was to assess the status and awareness of excessive heat exposure among agricultural workers.
METHODS: We selected a total of 90 farmers from a villages of Gyeongju-si, during August, 2015. We carried out the temperature measurement for nine times and derived Health Index (HI) and Wet Bulb Globe Temperature (WBGT) index. We compared the HI, WBGT and excessive heat warnings. Status of high temperature exposure, lifestyle, medical history, and awareness about excessive health related exposure illness assessed using survey questionnaires.
RESULTS: The matching rates between the WBGT and the HI during excessive heat warning were high, but when it was a non-excessive heat warning, there were days of excessive HI or WBGT. Out of 90 farmers surveyed, 78 cases (86.7%) were in their 60s and older age group. Slightly more than two third (71.1%) farmers were farming in the dawn-morning (71.1%), and the daily working hours were less than 4 hours (54.4 %), but only 23.3% among farmers took regular breaks. Of total, 14.4% farmers experienced excessive heat exposure related illness in order of tiredness, lethargy, dizziness, headaches, and sweating. Overall, the awareness of the danger for excessive heat and the heat wave warnings were high at 70.0% and 74.4%, respectively.
CONCLUSIONS: Politically, the excessive heat warnings should not be taken

into account the simple temperature measurement but, have to consider WBGT and HI standards at the same time. Farmers need to be promoted and educated to prevent the excessive heat related illness by periodically increasing their rest time during farming.

2: Environ Health Toxicol. 2018 ;33(1):e2018004. English.

Health effects of environmental pollution in population living near industrial complex areas in Korea.

Several epidemiological studies have reported an association between environmental pollution and various health conditions in individuals residing in industrial complexes. To evaluate the effects of pollution from industrial complex on human health, we performed a pooled analysis of environmental epidemiologic monitoring data for residents living near national industrial complexes in Korea. The respiratory and allergic symptoms and the prevalence of acute and chronic diseases, including cancer, were used as the outcome variables for health effects. Multiple logistic regression analysis was used to analyze the relationship between exposure to pollution from industrial complexes and health conditions.
After adjusting for age, sex, smoking status, occupational exposure, level of education, and body mass index, the residents near the industrial complexes were found to have more respiratory symptoms, such as cough (odds ratio [OR], 1.18; 95% confidence interval [CI], 1.06 to 1.31) and sputum production (OR, 1.13; 95% CI, 1.03 to 1.24), and symptoms of atopic

dermatitis (OR, 1.10; 95% CI, 1.01 to 1.20). Among residents of the industrial complexes, the prevalence of acute eye disorders was approximately 40% higher (OR, 1.39; 95% CI, 1.04 to 1.84) and the prevalence of lung and uterine cancer was 3.45 times and 1.88 times higher, respectively, than those among residents of the control area. This study showed that residents living in the vicinity of industrial complexes have a high risk of acute and chronic diseases including respiratory and allergic conditions. These results can be used as basic objective data for developing health management measures for individuals residing near industrial complexes.

3: J Prev Med Public Health. 2017 May;50(3):195-200. English.

Seroreactivity to Q Fever Among Slaughterhouse Workers in South Korea.

OBJECTIVES: Q fever is a zoonotic disease that occurs worldwide; however, little is known about its prevalence in South Korea. We attempted to determine the prevalence of Q fever seroreactivity among Korean slaughterhouse workers and the risk factors for seroreactivity according to the type of work.
METHODS: The study was conducted among 1503 workers at a total of 73 slaughterhouses and 62 residual-product disposal plants. During the study period, sites were visited and surveys were administered to employees involved in slaughterhouse work, and serological tests were performed on blood samples by indirect immunofluorescence assays. Serological samples

were grouped by job classification into those of slaughter workers, residual-product handlers, inspectors and inspection assistants, and grading testers and testing assistants. Employee risk factors were analyzed according to the type of work.
RESULTS: Out of 1481 study subjects who provided a blood sample, 151 (10.2%) showed reactive antibodies. When these results were analyzed in accordance with the type of work, the result of slaughter workers (11.3%) was similar to the result of residual-product handlers (11.4%), and the result of inspectors and assistants (5.3%) was similar to the result of grading testers and assistants (5.4%). Among those who answered in the affirmative to the survey question, "Has there been frequent contact between cattle blood and your mouth while working?" the proportions were 13.4 and 4.6%, respectively, and this was identified as a risk factor that significantly varied between job categories among slaughterhouse workers.
CONCLUSIONS: This study found a Q fever seroreactivity rate of 10.2% for slaughterhouse workers, who are known to be a high-risk population. Contact with cattle blood around the mouth while working was the differential risk factor between job categories among slaughterhouse workers.

4: Ann Occup Environ Med. 2017 ;29(1):40. English.

Psychological effects of a disastrous hydrogen fluoride spillage on the local community.

BACKGROUND: On September 27, 2012, at 3:43 pm, a hydrogen fluoride spill

occurred in a manufacturing plant located at the 4th complex of the Gumi National Industrial Complex in Gumi City, South Korea. The present study aimed to evaluate the psychological effects of the hydrogen fluoride spill on the members of the community and to investigate their relationships with physical symptoms and changes in psychological effects occurring as time passed after the accident.

METHODS: The 1st phase involved a survey of 1359 individuals that was conducted 1 month after the spill, and the 2nd phase involved a survey of 711 individuals that was conducted 7 months after the accident. The questionnaires included items for assessing demographic characteristics, hydrogen fluoride exposure level, physical symptoms, and psychological status. Physical symptoms were assessed to determine the persistence of irritations. Psychological status was assessed to investigate the impact of event level using the Impact of Event Scale – Revised Korean version (IES-R-K), and the anxiety level was assessed using the Beck Anxiety Inventory (BAI).

RESULTS: As the hydrogen fluoride exposure level increased, the impact of event and anxiety levels increased significantly both 1 and 7 months after the accident ($p < 0.05$). The mean score of the impact of event levels decreased significantly from 33.33 ± 14.64 at 1 month after the accident to 28.68 ± 11.80 at 7 months after the accident ($p < 0.05$). The mean score of the anxiety levels increased significantly from 5.16 ± 6.59 at 1 month after the accident to 6.79 ± 8.41 at 7 months after the accident ($p < 0.05$). The risk of persistent physical symptoms at 7 months after the accident was significantly higher in females. The risk of persistent physical symptoms also increased

significantly, with increasing age, hydrogen fluoride exposure, and impact of event levels ($p < 0.05$).
CONCLUSIONS: The present study found that the impact of event level and anxiety level increased with increasing hydrogen fluoride exposure. Anxiety levels persisted even after time passed. The risk of persistent physical symptoms at 7 months after the accident was higher in females, and it increased with increasing age, hydrogen fluoride exposure level, and impact of event levels.

5: Epidemiol Health. 2017 ;39(1):e2017010. English.

Patterns of disease occurrence and management, and public health issues among Korean populations based on information and experiences obtained by field epidemiological studies in various situations with episodic stories never been told.

No abstract available.

Publication Types:
Book Review

6: J Agric Med Community Health. 2015 Sep;40(3):148-157. Korean.

Prevalence of Allergic Rhinitis between Urban and Rural Residents in a Local Community.

OBJECTIVES: The purpose of this study was to examine the prevalence of allergic rhinitis between urban areas in the adjacent areas to a steel industrial complex and rural areas and the impact of pollutants in the industrialized city on allergic rhinitis.
METHODS: From July 28 to August 9 of 2008, 1,043 residents of urban and rural areas in a local community had enrolled in health screening and questionnaire survey. One thousand thirty-three patients also underwent a skin prick test. Prevalence rates of allergic rhinitis were calculated according to residential areas, and the used statistical analysis were Fisher's exact test and chi-square test.
RESULTS: In a survey, the fraction of adults, who complained of sneezing, nasal obstruction and rhinorrhea, during a recent 1 year period, showed the significant difference between urban (30.5%) and rural areas (22.4%). The fractions of positive skin prick tests were not different between two areas in each age group. The prevalence of allergic rhinitis was 8.4% in urban areas and 6.9% in rural areas. Considering the age groups, the adults group only showed the significantly higher prevalence of allergic rhinitis in urban areas (8.2% vs. 3.7%).
CONCLUSIONS: Unlike the children and adolescents groups, the prevalence of allergic rhinitis in adults group was higher in the industrialized urban areas.

7: J Agric Med Community Health. 2015 Jun;40(2):53-61. Korean.

Epidemiological Investigation on an Outbreak of Enterotoxigenic E. coli among the Baseball Club Students of High School in Ulsan City, 2014.

OBJECTIVES: An outbreak of food poisoning occurred among the baseball club students at a high school in Ulsan city in 2014. An epidemiological investigation was carried out to examine the infection source and the transmission route of pathogen, and to prevent a recurrence.

METHODS: A questionnaire survey was conducted for 26 male students and 2 food handlers. Rectal swabs were examined in 7 students and the 2 food handlers, and an environmental investigation was performed. A retrospective cohort study was used to evaluate the association between risk factors and disease.

RESULTS: The attack rate was 35.7% (10 persons/28 persons) from June 9 to 14, and Enterotoxigenic E. coli ST/LT was isolated from 7 among 28 persons. The study revealed that no food was a significant risk factor for the outbreak. There were no connection between environmental factors and the outbreak.

CONCLUSIONS: The major risk factors for this outbreak were presumed to be the contaminated ice cube and ice making machines and eating ice cube from the machines. More strict personal and environmental hygiene need to be enforced to prevent such outbreaks.

8: J Prev Med Public Health. 2015 Jan;48(1):53-61. English.

A Survey on the Status of Hepatitis E Virus Infection Among Slaughterhouse

Workers in South Korea.

OBJECTIVES: The seroprevalence of hepatitis E virus (HEV) among high-risk groups overseas is high, but studies in these groups are rare in South Korea. We conducted the present study from April to November 2012 to obtain data on the seroprevalence and associated risk factors for HEV among slaughterhouse workers in South Korea.

METHODS: Slaughterhouse workers from 80 workplaces nationwide were surveyed in South Korea in 2012. The subjects comprised 1848 cases: 1434 slaughter workers and 414 residual products handlers. By visiting 80 slaughterhouses, which were mixed with 75 of which also performed residual products handling, we conducted a questionnaire survey for risk factors and obtained blood samples in order to determine the seropositivity and seroprevalence of HEV. Anti-HEV IgG and IgM were measured using HEV IgG and IgM enzyme-linked immunospecific assay kits and HEV antigen was measured by reverse transcription polymerase chain reaction (RT-PCR).

RESULTS: The seropositivity of anti-HEV IgG was 33.5% (slaughter workers 32.8% and residual products handlers 36.2%), and among the seropositive individuals the seroprevalence of anti-HEV IgM was 0.5% (slaughter workers 0.5%, residual products handlers 0.7%). The response rate of HEV-antigen as measured by RT-PCR was 0.2%. Risk factors significantly related to anti-HEV IgG seropositivity were age, sex , and working duration (slaughter workers only).

CONCLUSIONS: There were significant risk factors (sex, age, and working duration) for HEV identified in our study. All three positive cases for HEV-antigen by RT-PCR were related to pig slaughter but without statistical

significance. To prevent HEV, an educational program and working guidelines may be needed for high risk groups.

9: J Agric Med Community Health. 2014 Dec;39(4):222-229. Korean.

Awareness on Zoonoses among Pig Farmers in Korea.

OBJECTIVES: Pig farmers are known as the high risk group for the zoonoses, but the study for pig farmers to zoonoses has been rare in Korea. So we surveyed the awareness on zoonoses among pig farmers to suggest directions for education.

METHODS: We visited four regional spots (Gyeongsangnam-do, Gyeongsangbuk-do, Jeollanam-do, and Chungcheongnam-do) where the self education programs of Korea pork producers were convened. We conducted a questionnaire survey of the general, work-related characteristics and the awareness on zoonoses among 278 pig farmers.

RESULTS: The awareness rate of zoonoses itself, brucellosis, tuberculosis and salmonellosis were 52.9%, 85.6%, 87.8%, 89.6%, respectively. The awareness rate of the content related to zoonoses, "Human can be infected by the disease of pig", was 46.9%. Awareness rate of zoonoses tended to increase as the school career, and awareness rate of salmonellosis tended to increase as working duration of pig raising.

CONCLUSIONS: The pig is the principal reservoir of zoonoses. Therefore, effective working guidelines to prevent zoonoses for pig farmers must be developed and an educational program on zoonoses is needed for pig farmers.

Furthermore, publicity activities about the prevention of zoonoses are needed for high-risk groups.

10: J Prev Med Public Health. 2014 Jul;47(4):230-235. English.

Analysis of the Change of Health Status Among the Republic of Korea Air Force Soldiers During Military Service.

OBJECTIVES: The aim of this study was to investigate whether the health status of Republic of Korea Air Force (ROKAF) soldiers changed after one year of military service.
METHODS: We selected 483 ROKAF soldiers from the 11 749 recruits who participated in the 2011 physical examination. The selected soldiers underwent another physical examination in 2012 for advancement to senior airman. Data from 2011 and 2012 were merged. To collect data on lifestyle, a questionnaire was sent to all included subjects via the military intranet e-mail service.
RESULTS: The percentage of recruits with an abnormal alanine transaminase level (normal range <40 IU/L) decreased from recruitment (13.7%) to the following year (2.7%). Moreover, the percentage of obese soldiers (body mass index > or =25 kg/m2) decreased from recruitment (20.5%) to the following year (10.4%). There was a significant change in mean duration of exercise carried out each day before (0.8+/-1.3 hours) and after (1.0+/-0.7 hours) joining the ROKAF service.
CONCLUSIONS: These ROKAF soldiers were generally in good health before and

after joining the armed service. After one year of military service, the health status of most soldiers improved, especially with respect to body mass index and alanine transaminase level.

11: J Prev Med Public Health. 2014 May;47(3):177-181. English.

Epidemiological Investigation of an Outbreak of Salmonellosis in Gyeongju, Korea.

OBJECTIVES: A salmonellosis outbreak occurred within a community of Gyeongju residents who ingested catered food from a wedding in June 2009. We aimed to epidemiologically investigate the probable vehicle of the infection.
METHODS: We conducted a retrospective cohort study on 34 local residents who ingested the wedding food.
RESULTS: Among the 34 residents, 31 (91.2%) reported symptoms of infection after eating the food. Among all of the wedding foods, pan-fried foods were highly associated with the diarrheal attack rate. On bacteriological examination, Salmonella species were detected in the pan-fried foods among the leftover foods and in 17 of the 31 stool specimens from the cases. There were five different types of pan-fried foods, but the onset of symptoms was independent of the ingredients used. We found that the pan-fried food was prepared at a food store in Seoul and that eggs were a common ingredient.
CONCLUSIONS: The major cause of the salmonellosis in this population was

presumed to be the pan-fried food prepared with contaminated eggs. These food items might have been partially undercooked because of their irregular shape, which allowed the Salmonella species to survive and multiply before ingestion.

12: J Prev Med Public Health. 2014 May;47(3):144-149. English.

Airborne Nicotine Concentrations in the Workplaces of Tobacco Farmers.

OBJECTIVES: Nicotine is a natural alkaloid and insecticide in tobacco leaves. Green tobacco sickness (GTS) is known as a disease of acute nicotine intoxication among tobacco farmers. Until now, GTS has been recognized globally as a disease that results from nicotine absorption through the skin. However, we assumed that GTS might also result from nicotine inhalation as well as absorption. We aimed to measure the airborne nicotine concentrations in various work environments of Korean tobacco farmers.

METHODS: We measured the nicotine concentrations in the tobacco fields, private curing barns, and joint curing barns of farmers from July to October 2010. All sampling and analyses of airborne nicotine were conducted according to the National Institute for Occupational Safety and Health manual of analytic methods.

RESULTS: The airborne nicotine concentrations (geometric mean [geometric standard deviation]) in the tobacco field were 83.4 mg/m3 (1.2) in the upper region and 93.3 mg/m3 (1.2) in the lower region. In addition, the

nicotine concentration by personal sampling was 150.1 mg/m3. Similarly, the nicotine concentrations in the private curing barn, workers in curing barns, the front yard of the curing barn, and in the joint curing barn were 323.7 mg/m3 (2.0), 121.0 mg/m3 (1.5), 73.7 mg/m3 (1.7), and 610.3 mg/m3 (1.0), respectively.
CONCLUSIONS: The nicotine concentration in the workplaces of tobacco farmers was very high. Future studies should measure the environmental concentration of nicotine that is inhaled by tobacco farmers.

13: Yonsei Med J. 2014 Mar;55(2):435-441. English.

Low Compliance with National Guidelines for Preventing Transmission of Group 1 Nationally Notifiable Infectious Diseases in Korea.

PURPOSE: This study was performed to evaluate the compliance with, and adequacy of, the Korean national guidelines which had been recommended until 2011 for isolation of patients with group 1 nationally notifiable infectious diseases (NNIDs), namely cholera, typhoid fever, paratyphoid fever, shigellosis, and enterohemorrhagic Escherichia coli (EHEC) infection.
MATERIALS AND METHODS: We evaluated the clinical and microbiological characteristics of confirmed cases of group 1 NNIDs and compliance with the guidelines in 20 Korean hospitals nationwide in 2000-2010. We also compared the Korean guidelines with international guidelines.
RESULTS: Among 528 confirmed cases (8 cases of cholera, 232 of typhoid

fever, 81 of paratyphoid fever, 175 of shigellosis, and 32 EHEC infections), strict compliance with the Korean guideline was achieved in only 2.6% to 50.0%, depending on the disease. While the Korean guidelines recommend isolation of all patients with group 1 NNIDs, international guidelines recommend selective patient isolation and screening for fecal shedding, depending on the type of disease and patient status.
CONCLUSION: Compliance with the previous national guidelines for group 1 NNIDs in Korea was generally very low. Further studies are needed to evaluate whether compliance was improved after implementation of the new guideline in 2012.

14: J Prev Med Public Health. 2013 Mar;46(2):62-73. English.

Epidemiological Investigation of an Outbreak of Hepatitis A at a Residential Facility for the Disabled, 2011.

OBJECTIVES: An outbreak of hepatitis A occurred at a residential facility for the disabled in July 10, 2011. This investigation was carried out to develop a response plan, and to find the infection source of the disease.
METHODS: A field epidemiologist investigated the symptoms, vaccination histories, living environments, and probable infection sources with 51 residents and 31 teachers and staff members. In July 25, 81 subjects were tested for the hepatitis A virus antibody, and specimens of the initial 3 cases and the last case were genetically tested.

RESULTS: Three cases occurred July 10 to 14, twelve cases August 3 to 9, and the last case on August 29. Among the teachers and staff, no one was IgM positive (on July 25). The base sequences of the initial 3 and of the last case were identical. The vehicle of the outbreak was believed to be a single person. The initial 3 patients were exposed at the same time and they might have disseminated the infection among the patients who developed symptoms in early August, and the last patient might have, in turn, been infected by the early August cases.

CONCLUSIONS: The initial source of infection is not clear, but volunteers could freely come into contact with residents, and an infected volunteer might have been the common infection source of the initial patients. Volunteers' washing their hands only after their activity might be the cause of this outbreak. Although there may be other possible causes, it would be reasonable to ask volunteers to wash their hands both before and after their activities.

15: J Korean Med Sci. 2012 Nov;27(11):1283-1284. English.

Health Care Plan for Hydrogen Fluoride Spill, Gumi, Korea.

No abstract available.

Publication Types:

Editorial

16: J Agric Med Community Health. 2012 Mar;37(1):1-11. Korean.

Comparison of the Awareness and Knowledge of Scrub Typhus between Case and Control Groups.

OBJECTIVES: To survey the awareness of patient to scrub typhus to provide data for education and communication concerning scrub typhus.
METHODS: Patients with scrub typhus (case group, n=299) and people without scrub typhus within the previous 2 years (control group, n=598) were matched for age (within 5 years), gender, and occupation (farmer or non-farmer). The participants were recruited from 15 study areas between October and December 2006.
RESULTS: The awareness rate of scrub typhus was 75.1%, and was significantly higher than in the case group (79.4% vs. 66.6%, respectively; p<0.01). The major routes of awareness were from 'past history of scrub typhus in family members or neighbors' (54.9%), 'television' (28.3%), and their past history of scrub typhus (5.5%). The average correct rate of scrub typhus was 48.4%, and the correct response rate of cases was significantly higher than controls (p<0.01). Especially, the correct rate of etiology, incubation period, route of transmission, and acquired immunity was <40%. Through conditional logistic regression test, the factor significantly associated with awareness in case group was age (odds ratio [OR], 0.96; 95% confidence interval [CI], 0.94-0.98). And the factors associated with awareness in control group were female (OR, 1.56; 95% CI, 1.03-2.36) age (OR, 0.98; 95% CI, 0.96-0.99), family history of scrub typhus (OR, 10.18; 95% CI, 1.37-75.99), history of receiving prevention

education (OR, 8.47; 95% CI, 1.14-63.00).
CONCLUSIONS: The rate of awareness was relatively low in study population. Thus, effective working guidelines and educational program to prevent scrub typhus must be developed, and publicity activities about the prevention of scrub typhus are needed for high-risk groups.

17: J Prev Med Public Health. 2012 Mar;45(2):78-89. English.

Vaccine Storage Practices and the Effects of Education in Some Private Medical Institutions.

OBJECTIVES: Although vaccination rates have increased, problems still remain in the storage and handling of vaccines. This study focused on inspecting actual vaccine storage status and awareness, and comparing them before and after education was provided.
METHODS: In the primary inspection, a status survey checklist was completed by visual inspection. A questionnaire on the awareness of proper vaccine storage and handling was also administered to vaccine administrators in private medical institutions in 4 regions in Gyeongsangbuk-province. One-on-one education was then carried out, and our self-produced manual on safe vaccine storage and management methods was provided. In the secondary inspection, the investigators visited the same medical institutions and used the same questionnaire and checklist used during the primary inspection. The results before and after education were compared, by treating each appropriate answer as 1 point.

RESULTS: The average checklists score was 9.74 (out of 15 points), which increased significantly after education was provided (by 0.84, $p<0.001$). The participants demonstrated improved practices in recording storage temperatures ($p=0.016$), storing vaccines in the center of the refrigerator ($p=0.004$), storing vaccines with other medication and non-medical items ($p=0.031$) after education. The average score calculated from the questionnaires was 10.48 (out of 14 points), which increased after education (by 1.03, $p<0.001$).
CONCLUSIONS: This study suggests that vaccine storage practices and awareness are inadequate, but can be partially improved by providing relevant education. Repetitive education and policy-making are required to store vaccines safely because one-off education and unenforced guidelines offer limited efficacy.

18: Korean J Audiol. 2011 Sep;15(2):62-66. English.

A Study on the Possibility of Occupational Noise-Induced Hearing Loss in Firefighters.

BACKGROUND AND OBJECTIVES: Firefighters face serious risks to their health and safety in the performance of their duties. In addition to the diverse occupational hazards well-known to the public, firefighters are also occasionally exposed to high levels of noise, such as sirens, horns, and electronic alerting signals.
MATERIALS AND METHODS: We first measured the noise emitted by two fire

trucks and one ambulance. Next, we enrolled 171 firefighters (164 males, seven females). We designated the employees of a private school as controls for the firefighter group. After selecting workers, including audiometric testing at 1,000 and 4,000 Hz, the groups were age- and gender-matched. Both groups were included separately for the right and left ears at pure-tone test frequencies at 1,000 and 4,000 Hz. We chose the better ear thresholds and analyzed the differences in hearing levels at each frequency and each age group between the firefighters and controls.
RESULTS: The sound pressure levels of the siren in and out of an ambulance, the first fire truck, and the second fire truck were 99.3 dB (A) and 108.9 dB (A), 92.3 dB (A) and 108.3, and 78.8 dB (A) and 99.0 dB (A), respectively. At 4,000 Hz, the hearing threshold was significantly increased by work period ($p<0.01$). Each hearing threshold level was significantly higher than controls ($p<0.01$).
CONCLUSIONS: Many of the noise sources produce sounds exceeding 90 dB (A), and some firefighters may be exposed for brief periods to levels that exceed 105-110 dB (A). The hearing threshold level in firefighters is higher than the general population and noise-induced hearing loss in firefighters is possible. In the future, consistent, effective, and long-standing implementation of hearing conservation programs are needed, and special health examinations for hearing levels in firefighters must be conducted.

19: Korean J Parasitol. 2011 Sep;49(3):273-275. English.

Hypersensitive Reaction to Praziquantel in a Clonorchiasis Patient.

Praziquantel is the drug of choice for clonorchiasis. Since clonorchiasis is endemic in most river basins, praziquantel has been widely used for 30 years in Korea. A 54-year-old Korean woman suffered from hypersensitive reactions, such as nausea, dyspnea, rash, and urticaria after taking the first dose of praziquantel to treat clonorchiasis. She ingested one dose again and the same symptoms appeared, and she was treated at a clinic with anti-histamines. She tried one more dose with anti-histamines but found the same symptoms. Later, she was found to pass eggs of Clonorchis sinensis and medicated with flubendazole. The hypersensitive reaction to praziquantel is rare but occurs. This is the 5th case report in the world.

20: J Prev Med Public Health. 2011 Mar;44(2):65-73. Korean.

Epidemiological Investigation for Outbreak of Food Poisoning Caused by Bacillus cereus Among the Workers at a Local Company in 2010.

OBJECTIVES: In July 2 2010, a diarrhea outbreak occurred among the workers in a company in Gyeungju city, Korea. An epidemiological investigation was performed to clarify the cause and transmission route of the outbreak.
METHODS: We conducted a questionnaire survey among 193 persons, and we examined 21 rectal swabs and 6 environmental specimens. We also delegated the Daegu Bukgu public health center to examine 3 food service employees and 5 environmental specimens from the P buffet which served a buffet on

June 30. The patient case was defined as a worker of L Corporation and who participated in the company meal service and who had diarrhea more than one time. We also collected the underground water filter of the company on July 23.

RESULTS: The attack rate of diarrhea among the employees was 20.3%. The epidemic curve showed that a single exposure peaked on July 1. The relative risk of attendance and non-attendance by date was highest for the lunch of June 30 (35.62; 95% CI, 2.25 to 574.79). There was no specific food that was statistically regarded as the source of the outbreak. Bacillus cereus was cultured from two of the rectal swabs, two of the preserved foods and the underground water filter. We thought the exposure date was lunch of June 30 according the latency period of B. cereus.

CONCLUSIONS: We concluded the route of transmission was infection of dishes, spoons and chopsticks in the lunch buffet of June 30 by the underground water. At the lunch buffet, 50 dishes, 40 spoons, and chopsticks were served as cleaned and wiped with a dishcloth. We thought the underground water contaminated the dishes, spoons, chopsticks and the dishcloth. Those contaminated materials became the cause of this outbreak.

21: J Agric Med Community Health. 2010 Dec;35(4):405-416. Korean.

Factors Related to the Disability of Stroke Patients in Gyeongju, Korea.

OBJECTIVES: This study was conducted to evaluate factors related to the degree of disability of stroke patients in a rural community.

METHODS: The study subjects included 641 patients selected from 975 stroke patients over the age of 50 with brain lesion-related disabilities living in Gyeongju, Korea, an urban-rural consolidated community. The data were obtained by interviews from July to October 2008. The subjects were divided into a seriously disabled group (1st and 2nd disability grade) and mildly disabled group (3th, 4th, 5th and 6th disability grade) by degree of disability according to registered disability grade. The collected data were analyzed using the SPSS 14.0 statistical package.
RESULTS: The study subjects included 379 males (59.1%) and 262 females (40.9%), and most patients were in their 70s (256 cases, 39.9%). The seriously disabled group included 287 patients (44.8%) and the mildly disabled group included 354 persons (55.2%). Age, prehospital delayed time and number of related chronic diseases were risk factors for serious disability ($p<0.05$).
CONCLUSIONS: Our results indicate that the degree of disability can be reduced by decreasing prehospital delayed time. We suggest that education of high risk groups and establishment of emergency services as well as a transport system for stroke patients are required to reduce the degree of disability due to stroke.

22: J Agric Med Community Health. 2010 Dec;35(4):361-369. Korean.

Epidemiological Investigation on an Outbreak of Norovirus Infection at a High School in Gyeongju City, 2009.

OBJECTIVES: An outbreak of norovirus occurred at a high school in Gyeongju city in 2009. An epidemiological investigation was carried out to examine the infection source and the transmission route of norovirus, and to prevent a recurrence.
METHODS: A questionnaire survey was conducted for 520 male students and 8 food handlers. Rectal swabs were examined in 21 symptomatic students and the 8 food handlers by Gyeongsangbukdo Government Public Institute of Health & Environment, and an environmental investigation was performed. A case-control study was used to evaluate the association between risk factors and disease.
RESULTS: The attack rate was 21.3% (111/520) between January 29 and February 10, and norovirus GII was isolated from 12 of 21 students. Food handlers had no symptoms and their stool samples were negative. The case-control study revealed that seasoned soy bean sprouts {odds ratio (OR): 2.542, 95% CI=1.315-4.915} and drinking water from the purifiers in the cafeteria (OR: 2.854, 95% CI=1.107-7.358) supplied on February 3 were significant risk factors for the outbreak. Water pipes and waste pipes were located in the same place where was filled with some water and trace of high water level was detected.
CONCLUSIONS: The major risk factors for this norovirus outbreak were presumed to be the contaminated seasoned soy bean sprouts and drinking water from the purifiers in the cafeteria. More strict personal and environmental hygiene need to be enforced to prevent such outbreaks.

23: J Agric Med Community Health. 2010 Dec;35(4):339-349. Korean.

Awareness of Major Zoonoses among Dairy Farmers in Gyeonggi Province.

OBJECTIVES: We surveyed awareness levels of brucellosis, Q fever and enterohemorrhagic Escherichia coli (EHEC) among dairy farmers in Gyeonggi Province to suggest directions for public education and public relations.
METHODS: We designed questionnaires to evaluate awareness of 3 major zooneses. We conducted a questionnaire survey to assess knowledge of the general characteristics of them, information sources for the awareness of zooneses, and the mode of transmission. Subjects were 716 workers from 482 dairy farms in Gyeonggi province.
RESULTS: The awareness levels for brucellosis, Q fever, and EHEC were 90.2%, 2.5% and 56.6%, respectively. Awareness of brucellosis and EHEC were tended to increase with higher number of school years. Television was the most common route of information for these zoonoses. Most common responses for questions concerning the method of transmission for each zoonoses, 'Contact with parturient fluid or placenta of animal' was 63.2% for brucellosis, 'Ingestion of raw meat or residual product' was 66.7% and 64.2% for Q fever and EHEC, respectively. The most common reason why dairy farmers think that it is difficult to prevent zoonoses was the inconvenience of wearing protection.
CONCLUSIONS: Education programs for zoonoses, especially Q fever, are needed for dairy farmers. In addition, publicity information activities about prevention of zoonoses are needed for high risk groups, such as the dairy farmers surveyed.

24: J Korean Med Assoc. 2010 Jun;53(6):483-491. Korean.

Health Disorders Caused by Physical Factors among Health Care Workers: Focusing on Ionizing Radiation.

Health care workers encounter many physical hazards in hospitals, including ionizing radiation, noise, and heat. Ionizing radiation is used in hospitals for diagnostic radiology, therapeutic radiology, dermatology, nuclear medicine, and in radiopharmaceutical laboratories. Radiation hazards may also exist in areas where radioactive materials are stored or discarded. Occupational exposure to ionizing radiation can lead to erythema or radiodermatitis. Ionizing radiation can cause gene mutation and chromosomal alteration. Other somatic effects that result from irradiation include several types of cancers (myelogenous leukemia, bone cancer, skin cancer, thyroid cancer, etc.), cataracts, aplastic anemia, and sterility.
The chief methods for reducing doses of ionizing radiation from external X-rays and gamma rays are to limit the time of exposure, increase the distance from the source of the exposure, shield the source with protective materials, and avoid unnecessary exposures. Improved equipment, knowledge, and reduced exposures can greatly reduce the risk of radiation exposure for workers. Many work areas in hospitals were identified as noisy enough to decrease productivity. Noise may trigger changes in cardiovascular, endocrine, neurological, and other physiological functions. Working in high-noise areas makes it difficult for workers to communicate among themselves or to concentrate on critical job functions. Many places in

hospitals including the laundry, boiler room, and kitchen may be hot during the summer months, especially in older facilities that have inadequate ventilation and cooling systems. Health care workers should pay attention to health disorders caused by physical factors to protect themselves against many hazards in hospitals.

25: Korean J Occup Environ Med. 2010 Jun;22(2):95-101. Korean.

The Association between Trans, Trans-Muconic Acid as a Biomarker for Benzene Exposure and the Platelet Count.

OBJECTIVE: Recent researches suggest that trans,trans-muconic acid (t,t-MA) would be an adequate metabolic biomarker for low-level benzene exposure. Few study have investigated the association between the t,t-MA and platelet count, and this is the primary aim of this study.
METHODS: From May 2004 to June 2004, 326 chemical factory workers took part in a questionnaire survey and they underwent urinary t,t-MA and CBC assessment. The questionnaire covered general aspects of health and the occupational and smoking histories. Statistical analyses of outcomes were performed using SPSS 14.0 for Windows.
RESULTS: The subjects' urinary t,t-MA concentration (geometric mean and geometric standard deviation) was 0.28 mg/g creatinine (2.62). No significant differences between different occupations among the chemical workers were found. The urinary t,t-MA concentrations in the smokers and non-smokers were 0.32 mg/g creatinine (2.44) and 0.25 mg/g creatinine

(2.77) respectively, and the difference was statistically significant ($p<0.05$). There was no significant difference in the platelet count based on occupation and the smoking history. There was significant correlation between log(platelet) and log(t,t-MA)(r=-0.116 p=0.039). The regression equation log(platelet) = -0.002xAge + [-0.029xlog(t,t-MA)] + 2.436, R2=0.032, was calculated from linear regression analysis with log(platelet) as the dependent variable. Cross-tabulation between 2 subgroups that were divided by the 90-percentile level of the t,t-MA (0.819 mg/g creatinine), and 2 subgroups that were divided by the 10-percentile level of the platelet count (175,000) was conducted. Higher distribution in subgroup with platelet count below 175,000 was demonstrated in the subgroup with a urinary t,t-MA above the 90-percentile, with an odds ratio of 3.01.
CONCLUSIONS: The study may be limited by not taking into account factors such as smoking quantity and sorbic acid, which may confound urinary the t,t-MA concentration as well as medication and infection that may affect the platelet count. Yet it is meaningful that the correlation between the urinary t,t-MA concentration and the platelet count was found through linear regression analysis and the chi-square test, and further, this outcome may be used as a basis for a study to establish the acceptable limit of urinary t,t-MA in Korea.

26: J Prev Med Public Health. 2010 May;43(3):205-212. English.

Work-Related Musculoskeletal Symptoms Among Dairy Farmers in Gyeonggi Province, Korea.

OBJECTIVES: The prevalence of work-related musculoskeletal symptoms (WMS) among Korean dairy farmers has not been investigated. The purpose of this study was to assess the prevalence of WMS and to evaluate the relationship between WMS and risk factors. METHODS: Self-developed questionnaires including the questionnaire developed by the Korean Occupational Safety and Health agency (KOSHA) were used to investigate WMS among dairy farmers in Gyeonggi Province, Korea. We informed selected dairy farmers about the study and sent the questionnaires by registered mail. They visited a public health center nearby or a branch of public health center on the appointed date and skillful researchers identified or conducted the questionnaires by interview. We analyzed 598 (32.8%) of the 1824 dairy farmers. Multiple logistic regression was implemented to estimate the odds ratios of risk factors. RESULTS: The mean age of the respondents was 50.4 +/- 8.7 years and the proportion of males was 63.0%. The prevalence of WMS at any site was 33.3%. The prevalence of neck WMS was 2.2%, shoulders 10.0%, arms/elbows 5.0%, hands/wrists/fingers 4.2%, low back 11.5%, and legs/feet 11.7%. The adjusted odds ratio of low back WMS for milking 4 or more hours per day was 4.231 (95% CI = 1.124 - 15.932) and statistically significant.
Low back WMS (2.827, 95% CI = 1.545 - 5.174) was significantly decreased by education. CONCLUSIONS: Low back WMS increased with milking hours and milking 4 or more hours per day was significantly associated with low back WMS. Low back WMS was significantly reduced with education. We hope that there will be increased attention about WMS in dairy farmers and the subject of future investigations.

27: J Agric Med Community Health. 2010 Mar;35(1):21-35. Korean.

Work-related Injuries and Diseases of Fisheries in Korea.

There are many work-related injuries and diseases of fisheries in Korea. Fishermen have exposed to harmful environment, such as natural disasters, ultraviolet, noise, vibration, organic solvents, ergonomic risk factors, loss of fresh water and food, and psychologic stress. They have suffered from numerous work-related health problems, including musculoskeletal disorders, cardiovascular diseases, infectious diseases, pyschological diseases, decompression sickness, and so on. Sea accidents can cause deaths of fishermen and if sea pollution is occurred, fishermen have a great economic damage. However, the concern of these injuries and diseases has been little because they are small in number and have decreased. Fisheries are important core industries, so the problems of fisheries have to be solved by a government. But work-related injuries and diseases are not investigated properly and accurate diagnoses are not made due to loss of concern. We hope that this article will be a cornerstone to solving the work-related problems of fisheries.

28: J Agric Med Community Health. 2009 Dec;34(3):368-374. Korean.

An Epidemiological Investigation on an Outbreak of Shigellosis during Travelling Cambodia and Vietnam among Korean Rural People, 2005.

OBJECTIVES: A group consisted of 32 Korean rural people had been travelled to Cambodia and Vietnam. During their travel for 7 days, first diarrheal case had occurred on 3rd day of travel and overall 8 people had diarrhea during travel. So we investigated the case for determination of the cause and route of infection.
METHODS: We interviewed all 32 people by visiting or telephone and took 23 stool samples from them, which are examined by Gyeungsangbuk-do Government Public Institute of Health & Environment in Korea. We interviewed travel agency for travel route and schedule.
RESULTS: The attack rate was 25.0% (8 cases) among 32 people. Of them, 3 cases were microbiologically confirmed to have the infection of Shigella flexneri (Serotype 3a). The result of this epidemiological investigation showed that the infection of shigellosis was happened in foreign countries. We assumed the water from Angkor-Watt was contaminated by S. flexneri. We could not able to test environmental specimens because of the infection is thought to be occurred in foreign countries.
CONCLUSIONS: Most common infectious diseases from foreign countries are transmitted by water or food. Travellers to foreign countries must take caution for communicable diseases and must do report and get appropriate treatment when symptoms developed after travel.

29: Korean Diabetes J. 2009 Dec;33(6):537-546. Korean.

The Status of Diabetes Mellitus and Effects of Related Factors on Heart

Rate Variability in a Community.

BACKGROUND: This study was performed to examine the status of diabetes mellitus (DM) in the community and effects of related factors on heart rate variability (HRV). METHODS: The author conducted HRV testing, a questionnaire survey, and blood chemistry analysis for fasting blood sugar (FBS) and HbA1c levels in 855 patients in a community over a period of 10 days, from August 14 to 25, 2006. The subjects were divided into a DM group and normal group by our study criteria. RESULTS: The proportion of DM was 12.6% and increased with old age. The mean measures of HRV (SDNN, Tp, Vlf, Lf, Hf, Lf/Hf) in the DM group were 22.7 (1.6) msec, 364.9 (2.7) msec2, 174.1 (3.0) msec2, 88.1 (3.2) msec2, 55.3 (3.2) msec2, and 1.6 (2.6), respectively, while those in the normal group were 32.2 (1.6) msec, 676.6 (2.8) msec2, 295.7 (3.1) msec2, 169.2 (3.4) msec2, 117.2 (3.2) msec2, and 1.4 (2.6), respectively. All parameters except for Lf/Hf were significantly lower in the DM group than in the normal group ($P < 0.01$). The Spearman's correlation coefficients between HRV and FBS or HbA1c were SDNN -0.222/-0.244 ($P < 0.01$), Tp -0.211/-0.212 ($P < 0.01$), Vlf -0.149/-0.132 ($P < 0.01$), Lf -0.188/-0.235 ($P < 0.01$), Hf -0.207/-0.204 ($P < 0.01$), and Lf/Hf ($P > 0.05$), respectively. CONCLUSION: This study shows that the DM group had a reduced HRV and increased pulse rate in comparison with the normal group. According to our results, the HRV test may be used accessorily for the early detection of cardiovascular autonomic neuropathy (CAN) and its related factors, as well as to prevent CAN.

30: J Agric Med Community Health. 2009 Sep;34(2):168-174. Korean.

Hygienic State and Awareness of Drink Vending Machines in a City.

OBJECTIVE: This study was conducted to investigate the hygienic state and awareness of drink vending machines in a city.
METHODS: Twelve of the most frequently used vending machines in various areas were selected in October 2008. As soon as fresh samples of milk coffee and adlay tea were collected in sterile containers from each machine, the temperature was measured. The samples were carried on ice to the laboratory to test total plate counts and Escherichia coli contamination. College students were inquired about drink vending machine hygiene by implementing self-developed questionnaires.
RESULTS: The temperature of 6 milk coffee samples (50.0%) and 8 adlay tea samples (66.7%) turned out to be inadequate. The total plate counts of milk coffee samples were all adequate, although 9 adlay tea samples (75.0%) were inadequate. All the beverage samples were negative for E. coli. In questionnaires obtained from 74 users of coffee vending machines, only 2 (2.7%) expected the hygienic state of vending machines to be good. There were 27 people (33.3%) that knew the existence of hygiene-related laws for vending machines.
CONCLUSIONS: This study reveals that the hygienic state of drink vending machines insufficiently reaches the standard for the hot beverage and that most people are not aware of importance of vending machine hygiene. It is necessary to make improvement in the hygiene of vending machines and public awareness in this city.

31: J Korean Soc Emerg Med. 2009 Aug;20(4):385-391. Korean.

Application of Sequential Organ Failure Assessment (SOFA) Score in the Prognostic Prediction of Paraquat Poisoning.

PURPOSE: To assess the ability of the Sequential Organ Failure Assessment (SOFA) score to predict the prognosis and organ dysfunction in the paraquat poisoning.
METHODS: We retrospectively evaluated 281 paraquat poisoning patients who arrived at Soonchunhyang University Cheonan Hospital between Sep 2007 and Dec 2008. Sixty eight patients (68) were excluded due to missing data. Finally, 213 patents were included in this study. We investigated the degree of paraquat exposure, the Glasgow coma scale, vital signs, blood laboratory tests, calculated SOFA score, and Yamaguchi Index on first hospital day. The prognostic prediction was compared between SOFA score and Yamaguchi Index.
RESULTS: The urine dithionite test, the amount of paraquat ingestion, arrival interval, aerum paraquat, Glasgow coma scale, HCO3-, potassium, and creatinine were significantly different between survivors and non-survivors. The Hosmer and Lemeshow Goodness-of-Fitness test of SOFA score ($p=0.419$) and Yamaguchi Index ($p=0.084$) indicated a good model performance. In the logistic regression analysis, p values the SOFA score and for the Yamaguchi Index were lower than 0.05 and the odds ratios were 1.930 and 0.108. At c-statistics, the area under receiver operator

characteristic curve of SOFA score was 0.807 and Yamaguchi Index was 0.865. 95% confidence SOFA score and Yamaguchi Index Intervals of did not include 0.5.

CONCLUSION: The SOFA score on the first day of hospital visit could reliably describe organ dysfunction and could distinguish survivors and non-survivors with reliable accuracy, as well as the Yamaguchi Index did in the case of paraquat poisoning.

32: J Prev Med Public Health. 2009 Jul;42(4):237-242. Korean.

Seroprevalence and Risk Factors of Brucellosis among Slaughterhouse Workers in Korea.

OBJECTIVES: The incidence of zoonoses in Korea has increased recently. However, the study of high risk groups for zoonoses has not been conducted to date in Korea. Thus, we did this study to obtain data on brucellosis among slaughterhouse workers in Korea. METHODS: We evaluated the structure of slaughterhouses and the process of slaughtering by reviewing the relevant literature and doing field studies. We visited 73 slaughterhouses and 62 residual products handle houses across the country. In addition, we conducted a questionnaire survey of the work activities, and obtained blood samples in order to determine the seroprevalence and risk factors of brucellosis. The titers of brucellosis antibodies were measured using the standard tube agglutination test (SAT). We diagnosed subjects as seropositive for Brucellosis if the titers were more than 1:160. The data

collected was evaluated using SPSS ver. 17.0. RESULTS: We included 1,503 subjects and obtained 1,482 blood samples among them: 849 workers involved in slaughtering, 351 handlers of residual products, 190 inspectors and their assistants, and 92 grading testers and their assistants. The seroprevalence of brucellosis among the slaughterhouse workers was 0.8% (95% CI=0.4-1.5). Broken down, the seroprevalence of brucellosis among the workers involved in slaughtering was 0.7% (95% CI=0.3-1.6), the handlers of residual products was 1.7% (95% CI=0.7-3.9) respectively. Risk factors for contracting brucellosis among slaughterhouse workers were being splashed with cattle blood around the mouth, cattle secretions around the body and not putting on protective apron while at work. CONCLUSIONS: An educational program is needed for high risk groups on zoonoses about the prevention of infection. Thus, effective working guidelines for workers who participate in the slaughter of animals must be developed in order to protect them from zoonoses.

33: J Agric Med Community Health. 2009 Jun;34(1):101-112. Korean.

Awareness of Zoonoses among Cattle Slaughterhouse Workers in Korea.

OBJECTIVES: Cattle slaughterhouse workers are known as the high risk group for the zoonoses. We surveyed the awareness of zoonoses among cattle slaughterhouse workers to suggest direction of education and public relations.

METHODS: We evaluated the epidemiological characteristics of zoonoses and

the process of cattle slaughtering. We designed different questionnaires for the workers involved in slaughtering, the handlers of residual products, the inspectors and their assistants, and the grading testers and their assistants respectively. We visited 73 cattle slaughterhouses and 62 residual products handle houses across the country and conducted a questionnaire survey of the general characteristics and the awareness of zoonoses. The data collected was evaluated using the SPSS 17.0 statistical package.
RESULTS: The awareness rate of brucellosis, Q fever and Enterohemorrhagic Escherichia coli (EHEC) were 83.9%, 18.5%, 62.7% among cattle slaughterhouse workers. The awareness rate of zoonoses among the workers involved in slaughtering and the handlers of residual products was lower than other workers. The awareness rate of zoonoses tended to increase as the school career. In the correct awareness rate of the transmission of zoonoses, 'transmission by tick bite', 'person to person' and 'air transmission' were lower than other transmission items. The most common reason why cattle slaughterhouse workers think that it is difficult to prevent zoonoses was the ignorance of how to prevent.
CONCLUSIONS: An educational program is needed for the cattle slaughterhouse workers on zoonoses especially Q fever. And publicity activities about prevention of infection are needed for high risk groups.

34: J Agric Med Community Health. 2009 Jun;34(1):24-33. Korean.

An Epidemiological Investigation on an Outbreak of Shigellosis in a Special

School for Handicapped in Yeongcheon-si and in a Rehabilitation Facility in Gyeongsan-si, Korea, 2008.

OBJECTIVES: In September 2008, an outbreak of diarrhea occurred among students attending Y school in Yeongcheon-si. Shigella sonnei was cultured from some of the rectal swabs. An epidemiological investigation was carried out to determine the source of the infection and the mode of transmission of the shigellosis outbreak.
METHODS: The index case lived in the D rehabilitation facility in Gyeongsan-si and an additional epidemiological investigation was carried out there. The cases could not be questioned due to their mental handicaps. The teachers were interviewed instead. A patient case was defined as a resident with diarrhea more than one time a day from September 18 to September 26, 2008 or a resident with confirmed Shigella sonnei at the Y school or the D rehabilitation facility.
RESULTS: The attack rate was 1.2% (8 persons) among 659 persons in the Y school and D rehabilitation facility. Five persons were microbiologically confirmed to have the infection and three persons were diagnosed on the basis of symptoms. Shigella sonnei was cultured from five of the 659 rectal swabs. However, 80 environmental specimens including drinking water, preserved foods, and cooking utensils were negative. All eight patients were Y school students and had been living in group boarding and lodging. Six of them lived in the D rehabilitation facility and two lived in the dormitory at the Y school. Five cases showed pulsed-field gel electrophoresis patterns that were identical for Shigella sonnei.
CONCLUSIONS: The results of this study showed that the infection source of

the shigellosis outbreak, in the two places, were identical. It is likely that the infections initially spread from a teacher or volunteer and then among the students.

35: J Korean Soc Clin Toxicol. 2009 Jun;7(1):41-43. Korean.

A Fatal Case of Methylene Chloride Poisoning.

A 52-year-old man was found dead in the driver's seat of his tank lorry. The tank lorry was used to transport industrial wastewater to a disposal plant; the material was transferred into a storage tank with the help of compressed air. The wastewater contained methylene chloride and 2-chloropyridine. No respiratory protective equipment was used while working under these conditions. The autopsy report showed extensive edema and congestion of the brain, lung, and intraperitoneal organs. The concentrations of methylene chloride in lung and brain were reported at 398 and 67 mg/kg, respectively.

36: Korean J Epidemiol. 2008 Dec;30(2):168-177. Korean.

An Epidemiological Investigation on an Outbreak of Bacillus Cereus Food Poisoning in a Girls' High School in Sangju-si, Korea, 2008.

PURPOSE: In May 2008, a diarrhea outbreak occurred among students of in a

girls' high school (S school) in Sangju-si, Korea. An epidemiological investigation was carried out to clarify the cause and transmission route of the outbreak.

METHODS: S school has been providing meals directly since July 2007. We conducted a questionnaire survey among 591 persons, and examined 283 rectal swabs and 98 environmental specimens. The patient case was defined as a member of S school who had diarrhea more than one time in a day, accompanied with one or more symptoms among abdominal pain or tenesmus from May 19th to May 26th 2008.

RESULTS: The attack rate was 24.0%. Bacillus cereus were cultured from three of the rectal swabs and five of the preserved foods. It was suspicious that contamination was possible in seasoning vegetables, and we found some foods were seasoned with spices after being cooled by moving cooler for about 20 minutes. Enterotoxin positive B. cereus were cultured from two foods cooled by moving cooler. Enterotoxin negative B. cereus were cultured from two environmental specimens of the moving cooler.

CONCLUSIONS: We presumed the cause of the diarrhea outbreak in S school was food poisoning by B. cereus. Because enterotoxin positive B. cereus were cultured from rectal swabs and foods, and the symptoms were corresponded. We estimated the outbreak was occurred by this process that B. cereus in the moving cooler contaminated foods during cooling and then rapidly proliferated.

37: J Korean Acad Rehabil Med. 2008 Jun;32(3):300-304. Korean.

New Method and Usefulness of Study on Sensory Nerve Conduction of Lateral Sural Cutaneous Nerve.

OBJECTIVE: To find a new method and usefulness of study on sensory nerve conduction of lateral sural cutaneous nerve (LSCN).

METHOD: The 60 extremities of 30 adults without peripheral neuropathy were placed in a prone position. Next, each subject was administered with an antidromic stimulation at a point 3 cm lateral from the center of the popliteal fossa. With the aid of active electrodes, the sensory nerve action potentials (SNAPs) were recorded at points 10 cm inferior and 1 cm lateral to the stimulation site. The method of sensory nerve conduction study suggested by Campagnolo et al. was performed simultaneously, to compare of the frequency of SNAPs and the amplitudes.

RESULTS: For the sensory nerve conduction study of the LSCN suggested in this report, SNAPs were obtained in 49 extremities, with a revelation rate of 81.7%. The mean amplitude was 11.91+/-3.68micronV. In the results of the tests suggested by Campagnolo et al., the SNAPs were obtained in 29 extremities, with a revelation rate of 48.3%. The mean amplitude was 8.37+/-5.21micronV. Significance testing of the electrodiagnostic method recommended in this study revealed that many SNAPs were observed for the LSCN, with statistically significant action potential amplitudes.

CONCLUSION: This study presents the new method and reference values of sensory nerve conduction for LSCN, which is thought to be useful in electrodiagnostic studies to diagnose entrapment neuropathy.

38: J Korean Soc Emerg Med. 2008 Apr;19(2):147-152. Korean.

Increase of Vertical Evacuation Interval in Proportion to the Number of Stories in High-rise Buildings.

PURPOSE: The purpose of this study is to determine whether the vertical evacuation interval, the required time for an ambulance to be dispatched after contact from a patient, increases with the number of stories above ground of a patient requesting evacuation from a high-rise building.
METHODS: The rescue diary of a 119 ambulance team dispatched to Jae-Song-Dong, Busan was retrospectively reviewed for 9 months for the period from April 1 to December 31, 2006. A total of 422 cases were included as subjects in the study. Median values of the vertical evacuation intervals collected from four building story groups were evaluated and the changing trend of the vertical evacuation intervals was analyzed.
RESULTS: Amang all cases, the median value of the vertical evacuation intervals and the inter-quartile range were 5.0+/-5.0 minutes. A comparison of the vertical evacuation intervals by the Kruskal-Wallis test revealed statistically significant differences between the four different story groups ($p<0.001$). An increase in building story number was shown to affect the vertical evacuation interval. The y-intercept was found to be 4.885, the regression coefficient was 0.133 ($p<0.001$), and the r-square value was 0.181. A similar result was found for patients having emergency symptoms.
CONCLUSION: These results confirm that there is an increase in vertical evacuation intervals depending upon the number of stories above ground of a patients requesting emergency medical service in high-rise buildings.

39: J Korean Med Sci. 2007 Dec;22(6):968-972. English.

Incidence and Risk Factors of Insulin Resistance Syndrome in 20-59 Year-Old Korean Male Workers.

We investigated the incidence of insulin resistance syndrome (IRS) according to the criteria of diagnoses suggested by the American College of Endocrinology/ American Association of Clinical Endocrinologists and the risk factors associated with the development of IRS. Among 2,048 subjects without a history of/or drug treatment for hypertension, diabetes, dyslipidemia with normal findings at baseline, 1,578 subjects aged 20-59 yr were followed prospectively for 2 yr. The incidence of IRS was 6.9 per 100 persons/year. The relative risk (RR) due to age was 1.03 (95% CI: 1.00-1.05) with every one-year increase in age. The RR associated with an abnormal waist-hip ratio group (> or =0.9) was increased by 1.74 (95% CI: 1.17-2.58) compared to the normal group (<0.9); RR associated with abnormal alanine transferase was increased (> or =35 IU/L) by 1.70 (95% CI: 1.20-2.41) compared to the normal group (<35 IU/L); and the RR associated with abnormal lowdensity lipoprotein (LDL) cholesterol was increased (> or =160 mg/L) by 1.70 (95% CI: 1.19-2.44) compared to the normal LDL cholesterol (<160 mg/L). Lastly, the RR of current smokers was increased by 1.63 (95% CI: 1.09-2.42) compared to that of non-smokers. It is necessary to develop methods of prevention and therapeutic approach to manage the integrated risk factors as opposed to individual factors.

40: Korean J Occup Environ Med. 2007 Sep;19(3):196-203. Korean.

Use of Screening Dose to Evaluate the Work-relatedness of Cancers Occurring among Korean Radiation Workers.

OBJECTIVES: To propose a screening dose based on the probability of causation (PC) to simplify the evaluation procedure for the work-relatedness of the cancers occurring among Korean radiation workers.
METHODS: Under the PC uncertainty distribution, the screening dose was defined in which the maximum PC is equivalent to 50% for single exposure to radiation, after which the values were calculated according to three significance levels: 90%, 95%, and 99%. By using the screening doses for each cancer, we also predicted the number of compensations for the occupational cancers among the current Korean radiation workers to determine a reasonable criterion for the significance level.
RESULTS: If the cumulative dose received by a radiation worker with cancer is less than the screening dose, then PC can not be greater than 50%, i.e., the case can not be awarded as an occupational disease. The numbers of compensation cases for a future decade were predicted to be 3-5, 4-6, and 6-10 at the significance levels of 90%, 95%, and 99%, respectively.
CONCLUSIONS: The procedure to evaluate the occupational disease was greatly simplified by using the screening dose for evaluation. A criterion of 95% was recommended as the PC upper limit following consideration for the results of the predicted cases for compensation and the economic

circumstances in Korea.

41: J Korean Med Assoc. 2007 Jul;50(7):582-591. Korean.

Contributing Factors of Infectious Waterborne and Foodborne Outbreaks in Korea.

Infectious waterborne and foodborne diseases pose a considerable threat to human health and the economy of individuals, families, and nations. They are the results of ingestion of contaminated water and food stuffs. They have increased recently in Korea. The reasons include the increase in international travels and trade, microbial adaptation, and changes in the food production system, human demographics and behavior as well as the climate change. The contributing factors of infectious waterborne and foodborne outbreaks in institutional settings and at home were reviewed through the epidemiological investigations of them. The most commonly reported diseases (possibly of waterborne origin) were typhoid fever, shigellosis, and viral hepatitis A. The sources of infection were any drinking water including well, spring, mountain, tap, and sea water. The water was contaminated with raining ground water, leakage from the damaged septic tank or pipes. The most commonly identified agents (possibly of foodborne origin) were norovirus, pathogenic E. coli, S. aureus, Salmonella spp., and V. parahemolyticus. The mechanisms of infection were raw food, secondary contamination of the raw food and unsafe storage, contaminations from food handlers, or contaminated water. While cholera was often due to

sea water, raw or under-processed seafood were important epidemiological pathways for cholera transmission. Owing to the globalization, imported cases of infectious waterborne and foodborne diseases have been increasing. We should recognize the outbreak rapidly and strengthen the surveillance. Also epidemiological investigations should start timely and be done thoroughly with repeat the situation, if necessary.

42: J Prev Med Public Health. 2007 Jul;40(4):285-290. Korean.

Seroprevalence of Brucellosis among Risk Population in Gyeongsangbuk-do, 2006.

OBJECTIVES: Cases of human brucellosis in Korea have recently increased due to the increasing incidence of bovine brucellosis. The authors conducted this study to elucidate the status of brucellosis through seroepidemiologic study.

METHODS: We selected our study population from a high risk group. We conducted a questionnaire survey and obtained blood samples to determine the seroprevalence of brucellosis antibodies for 10 days in February, 2005. The titers of brucellosis were measured by the combination of standard tube agglutination test (STA) and enzyme-linked immunosorbent assay (ELISA) test.

RESULTS: Our study subjects comprised 1,075 cases: 971 livestock workers, 51 veterinarians, and 53 artificial inseminators. In the STA test, 27 cases (2.5%) had titers of greater than or equal to 1:20. Of 1,068 cases (7 cases

were excluded due to previous brucellosis), 7 cases of brucellosis were diagnosed with titers of 1:160, giving a seroprevalence of brucellosis of 0.66%. The seroprevalence in the male group was 0.95%, and that of livestock workers, veterinarians, and artificial inseminators was 0.52%, 4.17%, and 0.00%, respectively. The Spearman's correlation coefficient between the positive rate of bovine brucellosis per capita and household and human brucellosis was 0.806 and 0.744, respectively. The concordance rate between the Korea National Institute of Health and the Gyeongsangbuk-do Institute of Health and Environment by the STA and ELISA tests was 94.7% and 100.0%, respectively.

CONCLUSIONS: The study results indicated in higher seroprevalence rate among veterinarians than among livestock workers and artificial inseminators. Because veterinarians may be exposed to this high risk, effective working guidelines for veterinarians to guard against brucellosis must be developed. Moreover, more extensive epidemiologic research for laboratory workers and meat handlers is needed.

43: J Prev Med Public Health. 2007 May;40(3):239-248. Korean.

Seasonal Variation of Food Intake in Food Frequency Questionnaire among Workers in a Nuclear Power Plant.

OBJECTIVES: This study was conducted to investigate the systematic error, such as seasonal change or inadequate food items, in a food frequency questionnaire administered to workers in a Nuclear Power Plant, Korea.

METHODS: We performed three repeat-tests with 28 subjects on May 13, July 8 and Dec 16, 1992. Our food frequency questionnaire (FFQ) comprised 84 foods organized into 7 food-groups, and was composed of the items of usual intake frequency (8 categories) and the amount per intake (3 or 4 categories) over the previous year. We compared the means of intake frequency and the frequency of the portion-size according to each season using Repeated Measures ANOVA and Pearson's chisquare test with Fisher's exact test.

RESULTS: We found the significant seasonal changes of several food items in intake frequency measurement. These items were typical seasonal foods such as mandarin orange, plum and green vegetables, while the single questions consisted of inadequate food items such as thick beef or similar soup and various kimchi products. Significant seasonal changes in portion-size were found in only two items: cooked rice-brown and fresh.frozen fishes.

CONCLUSIONS: The systematic errors observed could caused loss of validity in the FFQ. Consideration should be given for seasonal variation in FFQ survey and methodological concerns are needed to improve the quality for measuring usual diet pattern.

44: Korean J Occup Environ Med. 2007 Mar;19(1):1-16. Korean.

Occupational Diseases among Agricultural, Forestry and Fishery Workers Approved by Korea Labor Welfare Corporation.

OBJECTIVES: To analyze the characteristics of occupational diseases among agricultural, forestry and fishery workers compensated under the Industrial Accident Compensation Insurance that is operated by the Korea Labor Welfare Corporation (KLWC).
METHODS: Using the KLWC database, we collected 667 cases of occupational disease compensated during the 8 years between 1 January 1998 and 31 December 2005. We analyzed the characteristics of occupational diseases using the KLWC electronic data and the data investigated by the Korea Occupational Safety and Health Agency.
RESULTS: KLWC approved 667 cases, including 69 deaths (10.3%). Men accounted for 76.0%of the approved cases. The most common age group was 50~59 years of age (37.3%). The most common size of enterprise was 5 to 49 workers (47.5%). The proportion of occupational diseases was 54.1%, of which 45.9% was work-related. Among occupational diseases, the most common was skin diseases (147 cases, 40.7%), followed by infectious diseases (131 cases, 36.3%), including scrub typhus (123 cases) and intoxication (40 cases, 11.1%). The causal hazardous agents were biological (320 cases, 88.6%), chemical (29 cases, 8.0%), and physical (11 cases, 3.0%). The major type of industry and occupation were forestry (445 cases, 66.7%) and elementary (151 among 235 cases, 89.3%), respectively.
CONCLUSIONS: Major compensated occupational diseases were infectious diseases, including scrubtyphus, skin diseases and toxic disease, which occurred among workers who were older, and had short tenure and elementary occupation, and these workers were out of the occupational health policy. A management policy must be established to prevent occupational diseases occurring in such vulnerable workers.

45: J Korean Acad Rehabil Med. 2006 Dec;30(6):646-654. Korean.

Disability Duration in Musculoskeletal Injured Patients due to Automobile Accidents.

OBJECTIVE: The aims of this study were to refer descriptive statistics of the disability duration in musculoskeletal injured patients in the automobile accidents.
METHOD: The authors chose 469,319 injured patients in traffic accidents as subjects that met inclusion criteria with 8 representative Abbreviated Injury Scale (AIS) comparable with 4th edition of Korean standard classification of diseases. Mean, standard deviation, mode, median, maximum and minimum of 8 representative AIS code were calculated.
RESULTS: Mean disability duration for minor cervical sprain was 18.8 days, that of moderate cervical disc herniation was 56.2 days, that of serious cervical disc herniation was 141.5 days, that of minor lumbar sprain was 21.2 days, that of moderate lumbar disc herniation was 53.5 days, that of serious lumbar disc herniation was 128.1 days, that of sprain of 3 major joint in lower limb without desmorrhexis was 24.0 days, and that of dislocation or desmorrhexis of 3 major joint in lower limb was 101.2 days.
CONCLUSION: Disability duration of the representative musculoskeletal diseases comparable to AIS code in 4th edition of Korean standard classification of diseases was presented and this study can be utilized as a basic recommended disability duration of automobile injured persons.

46: J Korean Acad Fam Med. 2006 Oct;27(10):789-797. Korean.

Pattern of Hypertensive Management and Usefulness of Treatment Guideline in Primary Care.

BACKGROUND: This study aimed at finding out how much the rate of the JNC-7 guideline was carried out, and assessed the degree of evidence based clinical practice patterns and the validity of the new guideline by comparatively analyzing primary care practice patterns in hypertensive patients.
METHODS: A questionnaire regarding the management of hypertension was conducted by mail between May 15 and July 15, 2005, to 1,008 of the Korean Association of Family Medicine listed in the address book. There were 195 respondents with a response rate of 19.4%.
RESULTS: Among the total respondents, 91.1% were aware of the new guideline and had received information mainly through training lectures and seminars. They said that the biggest difference between the new guideline from the previous one was the inclusion of prehypertension (62.3%), and 61.1% were using the new term prehypertension in their practice. The most frequency advice given to patients related to lifestyle modification were in the order of smoking cessation (44.8%), exercise (41.7%) and weight reduction (38.0%). Calcium channel blocker was the most commonly used (60.9%) as the first-line agent. When physicians failed to control blood pressure with the first medication in cases of stage 1 hypertension, 67.7% added other

agents. In treating stage 2 hypertension, 59.9% started with a single agent and gradually added other agents.
CONCLUSIONS: Family physicians in primary care clinics had a good understanding of the JNC-7 guideline. However, the rate at which they applied it in treatment was low. To effectively apply the guideline in actual treatments, aggressive education of practitioners and improvement on medical system and treatment guidelines are needed.

47: J Prev Med Public Health. 2006 Sep;39(5):404-410. Korean.

One Year Follow-up Study of Symptomatic Cases of Ulnar Neuropathy at the Elbow in a Rural Population.

OBJECTIVES: This study examined the natural history of symptomatic patients who did or did not display abnormal results on nerve conduction studies (NCS).
METHODS: Forty hundred fifty adults were selected among a total of 578 residents who participated in the health examination in a rural Korean district. A symptom questionnaire and NCS were used to diagnose ulnar neuropathy at the elbow (UNE). There were 6.4% of the subjects with UNE, 5.1% of the subjects showed symptoms without a NCS, and 84.2% of the subjects who were asymptomatic. One year later, 20 symptomatic limbs with an abnormality on the ulnar NCS and 22 symptomatic limbs

without any abnormality in the ulnar NCS were enrolled in a follow-up study. The natural history of UNE was evaluated by examining the changes in the clinical and electrodiagnostic examinations.

RESULTS: The 1-year follow-up of the enrolled limbs found that for the symptomatic limbs with an abnormality on the NCS, the degree of severe of the clinical grade changed from 20% to 10%. In contrast, for the symptomatic limbs that were without any abnormality in the NCS, the change of the severe degree of the clinical grade was from 0% to 18.2%. Also, for the electrodiagnostic change, only symptomatic limbs without NCS abnormalities showed significant changes in motor latency, amplitude and conduction velocity at the 1-year follow-up.

CONCLUSIONS: The 1-year follow-up study revealed symptomatic limbs that were without any abnormality on the ulnar NCS were more likely to progress than the symptomatic limbs with an abnormality on the ulnar NCS.

48: J Korean Acad Rehabil Med. 2006 Aug;30(4):346-352. Korean.

The Two Year's Follow Up Study of Symptomatic Hands without Electrodiagnostic Evidence of Carpal Tunnel Syndrome.

OBJECTIVE: The purpose of this study was to determine the natural history

of symptomatic hands without electrodiagnostic evidence of carpal tunnel syndrome (CTS).
METHOD: This study was comprised of 88 hands of 49 subjects with symptoms consistent with CTS without median mononeuropathy who were recruited during a community health examination and followed after two years. 88 hands of 44 people with age and sex-matched healthy controls were followed for comparison. Symptoms and electrodiagnostic findings were compared between the two groups.
RESULTS: 62 hands of the symptomatic group had persistent symptoms after 2 years while 16 hands of the control group had symptoms consistent with CTS. Median motor distal latencies were significantly delayed after two years in the symptomatic group ($p<0.05$). Median sensory latencies were also delayed in the symptomatic group, but this was not statistically significant ($p=0.064$). The occurrence rate of median mononeuropathy at wrist was significantly higher in the symptomatic group than in the control group (13.6% vs. 2.3%)($p<0.05$).
CONCLUSION: The results of this study suggested that a significant number of symptomatic hands without electrodiagnostic evidence of CTS may have persistent symptoms and may progress to electrodiagnostically evident CTS.

49: J Korean Orthop Assoc. 2006 Aug;41(4):736-745. Korean.

AnAnalysis of the Length of Admission for Some Musculoskeletal Disordersamong the National Health Insurance Corporation Data.

PURPOSE :The goals of this study were to investigate the medical care utilization and statistics of lengths of admissions such as the means, modes, medians, maximums, and minimums for patients with some musculoskeletal diseases among the National Health Insurance Corporation data. We provided basic recommendations for the proper lengths of admissions of occupationally injured workers with these diseases in Korea.

METERIALS AND METHODS: The study subjects were 20 to 59 year-old patients who were admitted from January 1, 2000 to June 30, 2001, over 4-day lengths of admission for the management of 20 different musculoskeletal disorders. We calculated means, standard deviations, modes, medians, maximums, minimums, and points of inflection for the lengths of admission of patients with these disorders.

RESULTS: The mean length of admission for carpal tunnel syndrome was 8.0+/-6.2 days, that of Raynaud's syndrome was 10.5+/-7.7 days, that of internal derangements of the knee was 12.9+/-11.8 days, that of unspecified internal derangements of the knee was 13.3+/-13.0 days, that of cervical disc disorder with radiculopathy was 16.8+/-19.0 days, that of other cervical disc displacements was 15.9+/-15.9 days, that of lumbar and other intervertebral disc disorders with radiculopathy was 14.9 +/-13.8 days, that of unspecified intervertebral disc displacements was 14.9+/-13.9 days, that of unspecified synovitis and tenosynovitis was 12.5+/-9.9 days, that of ganglions was 7.5+/-7.3 days, that of fasciitis, NEC, was 19.8+/-24.7 days, that of adhesive capsulitis of the shoulder was 15.2+/-15.5 days, that of impingement syndrome of the shoulder was 11.4+/-12.0 days, that of medial epicondylitis was 11.2+/-7.3 days, that of lateral epicondylitis was 12.1+/-11.5 days, that of myalgias was 12.4+/-19.5 days, that of sprain and

strain of the cervical spine was 12.4+/-10.2 days, that of sprain and strain of the lumbar spine was 12.3+/-10.9 days, that of a current tear of the meniscus was 13.3+/-13.2 days, and that of sprain and strain involving the cruciate ligaments of the knee was 18.2+/-17.0 days. Every point of inflection was the 80th percentile or the 90th percentile of the lengths of admission of the subjects.

CONCLUSION: This study has significant weaknesses, such as limitations of applicable ranges and errors in the National Health Insurance Corporation data, among others. However, the authors present various statistics and points of inflection of lengths of admissions for patients suffering from 20 work-related musculoskeletal disorders in the general population. Those cases in which the lengths of admissions of patients with the above-mentioned musculoskeletal disorders that are greater than the points of inflection, need to be evaluated carefully.

50: Korean J Med. 2006 Jul;71(1):1-3. Korean.

Diagnosis of human brucellosis.

No abstract available.

Publication Types:

Editorial

51: Korean J Epidemiol. 2006 Jun;28(1):112-118. Korean.

Case Series of Pediatric Mycoplasma Pneumoniae Pneumonia Inpatients in A Hospital.

OBJECTIVES: The aim of this report is to examine characteristics of pediatric Mycoplasma Pneumoniae pneumonia inpatients.
METHODS: The authors conducted a medical record survey among 236 children under seventeen were hospitalized in a university hospital due to Mycoplasma Pneumoniae pneumonia, 2001-2003. The author reviewed gender, age, address, developed date of symptoms, admission day, discharge day, etc.
RESULTS: The number of inpatients was increased during late summer and fall. The mean age of all cases was 3.7+/-2.9 years old. One hundred ninety three cases (81.8%) had lung infiltration findings. The mean symptomatic period was 11.9+/-6.4 days (maximum 44 days, minimum 4 days, median 10 days), the mean hospital length of stay was 5.2+/-2.5 days (maximum 17 days, minimum 0 day, median 5 days).
DISCUSSION: The authors reviewed characteristics during last 3 years for Mycoplasma Pneumoniae pneumonia. This report is meaningful with its basic data for the epidemiologic characteristics of Mycoplasma Pneumoniae pneumonia inpatients.

52: Korean J Epidemiol. 2006 Jun;28(1):28-35. Korean.

Perspectives of Communicable Disease Surveillance in Korea.

Environmental and climatic changes and the mobility of ever-increasing numbers of people increase the risks for the emergence and reemergence of infectious diseases. Since communicable disease trends change rapidly, many nations have developed individualized communicable disease surveillance systems. In Korea, notification of the incidence of communicable diseases has been the most important form of surveillance since 1954. In addition, the government has established various surveillance systems since the late 1990s. Current problem areas of surveillance systems are the low reporting rate, a lack of representativeness, a lack of participation, and poor utilization. The government has not fully evaluated these systems. For many diseases, it is of critical importance to maintain the confidentiality of surveillance data. Issues of confidentiality are critical and must be considered in order to obtain valid data and protect those surveyed. In the future, we have to improve the reporting rate, enhance collaborations with veterinarians and gain the full support from the governmental departments of agriculture and defense. Surveillance systems should be evaluated regularly. The most dynamic and important part of surveillance is the feedback mechanism. To develop positive feedback, we must disseminate the collected and analyzed information and give reimbursement to the reporters. We have to built close partnerships with governmental agencies, international organizations, research institutes, private health corporations, and academia.

53: J Korean Rheum Assoc. 2006 Mar;13(1):18-25. Korean.

Prevalence and Risk Factors of Fibromyalgia Syndrome and Chronic Widespread Pain in Two Communities in Korea-First Report in Korean.

OBJECTIVE: To estimate the prevalence and risk factors of fibromyalgia syndrome (FMS) and chronic widespread pain (CWP) in Korean.
METHODS: Those who participated chronic pain field study in Uljin and Pohang, Kyongsangbuk-do in 2004 were evaluated for the prevalence of FMS and CWP. Diagnosis of FMS and CWP were made using American College of Rheumatology criteria. All 1,028 were interviewed based on a detailed questionnaire and 144 CWP patients were examined for tender points.
RESULTS: Among the 1,028 participants (mean age+/-SD, 63.3+/-12.7 years), 676 participants were female (mean age+/-SD, 62.1+/-13.5 years) and 352 participants were male (mean age+/-SD, 65.7+/-10.5 years). There were 23 cases of FMS (2.2%), which consisted of 21 female cases and 2 cases of male. Prevalence of FMS was significantly higher in female compared to male (3.1% vs 0.6%, p<0.01). Prevalence of FMS showed increasing trend in the ascending order of age in decade (p<0.001). CWP was detected in 144 cases (14.0%), which consisted of 130 female cases and 14 cases of male. Prevalence of CWP was also significantly higher in female compared to male (19.2% vs 4.0%, p<0.001). Prevalence of CWP also showed increasing trend in the ascending order of age in decade (p<0.001). Except for age and sex, hating vegetables is an associated factor for the development of FMS in CWP [OR 18.7 (95% CI 2.4, 145.3)] and diabetes is a risk factor for the development of FMS in normal population [OR 3.7 (95% CI 1.1, 11.9)] on

multivariate analyses.

CONCLUSION: The prevalence of FMS and CWP were 2.2% and 14.0% in Korean, respectively. The prevalence of FMS and CWP were significantly higher in female and aged individuals. Aging, female sex, hating vegetables and diabetes are prognostic factors for the development of FMS.

54: J Prev Med Public Health. 2006 Mar;39(2):105-109. Korean.

Future of Scientific Research on Preventive Medicine in Korea.

The Korean Society of Preventive Medicine has undergone continuous development, after overcoming the difficult early years, in the 59 years since its establishment in 1947. It has repeatedly upgraded its quality and quantity of research with the first journal edition in 1968 and the continuous increase in publication numbers, scientific articles, joint research projects, intra-field exchanges and participation in various international scientific activities. In the future, we should gather a more extensive collection of opinions regarding the introduction of clinical preventive medicine specialists and prepare for the establishment of a training program for clinical specialists into a preventive medicine residency course. Moreover, we should raise interest in the importance of protecting individual information and maintaining medicine ethics. It's impossible to develop academic activities without cooperation. We need such cooperation with basic medical approaches across a wide range of fields. Furthermore, we should strengthen our cooperation with aspects of clinical

and drug epidemiology in many fields including public health, statistics, and dietetics. Finally, we should raise the level of international cooperation with many countries, including North Korea, to prevent diseases and promote health. Preventive medicine is a science in which practice is as important as theory. We must aim to nurture preventive medicine specialists who practice in many areas of society with the goal of preventing diseases, promoting health, improving fertility, and securing healthy elderly life for individuals and the entire population. To this end, we will endeavor to promote both theoretical and practical components of academic development.

55: J Korean Acad Rehabil Med. 2006 Feb;30(1):33-39. Korean.

Characteristics of Disability Recuperation Musculoskeletal Diseases at a Ship-Yard.

OBJECTIVE: This study was performed to analyze the length of work-related musculoskeletal disability and to identify the various related factors of disability duration.
METHOD: Approved cases work-related musculoskeletal disease between Jan 1, 2000 and Jan 6, 2004 were collected by using the workers' compensation company's database and questionnaire. The cases were comprised of 16 more frequent musculoskeletal diseases. 551 approved musculoskeletal disease cases were composed of 295 recuperated cases and 256 recuperating cases.
RESULTS: The average length of work-related musculoskele- tal disability

were 420.3+/-246.2 day in the recuperated group and 428.0+/-328.9 day in the recuperating group. The mean disability length of musculoskeletal diseases were over one year with similar distribution except for ganglion. The length of disability were not affected by age, employment duration, type and number of co-mobidity, time from claim receipt to allowance, type of treatment. But, operation history and accident type were affected to the length of disability.
CONCLUSION: The results suggest that the length of work- related musculoskeletal disability was not determined by biomedical disease related factors, however, it determined by social and social insurance systemic factors.

56: J Korean Diabetes Assoc. 2006 Jan;30(1):73-81. Korean.

Glycemic Control and Health Behaviors through Diabetes Mellitus Education in a Clinic.

BACKGROUND: This study was carried out to examine the changes in the health behaviors and glycemic control before and after administering a Diabetes Mellitus (DM) education program in a clinic.
METHODS: The author conducted a questionnaire and analyzed the blood chemistry with the fasting plasma blood sugar (FBS) and hemoglobin A1c (HbA1c) level of 80 patients in a clinic for 6 months from February to July 2004. The study group was divided into a poorly controlled (PC) group and well-controlled (WC) group according to the FBS or HbA1c level. The author

then educated the subjects about general knowledges for DM over a 6-month period. The changes in the results before and after the DM education were measured as the changes in the health behaviors along with the changes in the FBS, and HbA1c levels.
RESULTS: The study subjects contained 20 males and 20 females in each groups, and the major age group was the fifth decade (22 cases, 27.5%). The mean values for the total health behavior scores after the DM education program in the PC and WC group were 16.2 +/- 1.9, and 16.2 +/- 1.7 respectively, and were significantly higher than that before the education program (11.4 +/- 2.1, 15.3 +/- 1.9, P < 0.05). The mean FBS levels after the DM education program in the PC and WC groups were 130.2 +/- 22.8 mg/dL, and 116.2 +/- 16.6 mg/dL respectively, and was significantly lower than that before the education program (186.3 +/- 33.5 mg/dL, 135.3 +/- 16.3 mg/dL, P < 0.05). The mean HbA1c levels after the DM education program in the PC and WC groups were 7.0 +/- 0.8%, and 6.2 +/- 0.4% respectively, which were significantly lower than that before the education program (9.2 +/- 1.4%, 6.5 +/- 0.4%, P < 0.05).
CONCLUSION: This study suggests that a DM education program in a clinic is effective in improving the health behaviors and laboratory findings in DM patients.

57: Korean J Epidemiol. 2005 Dec;27(2):70-79. Korean.

A Study on the Epidemiologic Characteristics of Scrub Typhus in Gyeongsangbuk-do, 1999-2001.

ONJECTIVE: This study was carried out to investigate the epidemiologic characteristics and magnitudes of scrub typhus (tsutsugamushi disease) in Gyeongsangbuk-do, Korea.
METHODS: Resources of data collected were epidemiologic surveillance report to public health centers on each disease occurrence. The authors reviewed 344 epidemiologic surveillance reports for cases of scrub typhus developed in Gyeongsangbuk-do, Korea from Jan 1, 1999 to Dec 31, 2001.
RESULTS: The scrub typhus cases were 376 during three year period, and the incidence was significantly higher in the county area than in the city area. Incidence of scrub typhus was increasing each year. In 344 epidemiologic surveillance reports, the job of the cases was farmer (67.2%), housewife (4.7%), clerk (3.5%). Two hundred ninety eight cases (86.6%) have experienced outdoor activities before development of the scrub typhus. The outdoor places of exposure were dry field (64.8%), rice field (51.7%), hill (12.8), and grass field (9.7%). The symptoms reported were fever (93.3%), headache (83.7%), chill (69.5%), and rash (54.4%).
CONCLUSIONS: Incidence of scrub typhus was increasing in Gyeongsangbuk-do, Korea, this phenomenon may be related to change of climate, the reporting rate of disease and outing activities. Good surveillance and adequate analysis of epidemiologic data is a essential for the prevention of the disease. With this result, high risk population was identified and effective intervention should be done to prevent the disease.

58: J Prev Med Public Health. 2005 Nov;38(4):482-488. Korean.

Investigation of a Series of Brucellosis Cases in Gyeongsangbuk-do during 2003-2004.

OBJECTIVES: We conducted an investigation on 14 cases of brucellosis in Gyeongsangbuk-do during 2003-2004 to understand the source of infection and the transmission routes of brucellosis. METHODS: The authors visited the each of the health centers and we examined the patients, their written epidemiologic questionnaire and the occurrence of bovine brucellosis. We visited the patients' living and work areas, and we examined their occupations, the date they developed symptoms, the progress of their symptoms, whether or not they were treated, their current status, whether or not they consumed raw milk and raw meat, and if their work was related to cattle breeding and the related details. We reviewed the results of the blood tests and medical records and we examined the cattle's barn. RESULTS: There were 3 patients in 2003 and 11 patients in 2004. All of their brucella antibody titer exceeded 1: 160. The patients' symptoms were fever, myalgia, malaise, chills and an influenza-like illness, but the clinical signs were absent on the medical records. Brucella abortus were cultured from 3 of the patients' blood samples. CONCLUSIONS: When the authors discovered the transmission routes, they were divided into 4 different sorts. The first route was related to cattle birth such that patients touched the calves or placentas that were infected with the Brucella species. The second route was related to performing artificial insemination on the cattle and the semen that was used for artificial insemination. The third route was due to the ingestion

of raw meat and milk. The last route was due to sexual intercourse between the patients.

59: J Prev Med Public Health. 2005 Nov;38(4):457-464. Korean.

Epidemiologic Investigation on an Outbreak of Salmonellosis in Yeongcheon-si, 2004.

OBJECTIVES: An outbreak of salmonellosis occurred among the students and staff of D primary school in Yeongcheon-si, 2004. This investigation was carried out to prevent any recurrence of this outbreak and to study the infection source and transmission of the salmonellosis. METHODS: The authors conducted a questionnaire survey among 1, 205 students and staff members from D primary school about the ingestion of the school lunch and drinking water, and the manifestation of their symptoms. The author examined rectal swabs, the tap water and microorganism cultures, and we also investigated the dining facility and water supply facility. RESULTS: The diarrheal cases were defined as the confirmed cases or the persons who had diarrhea more than one time with symptoms such as fever, vomiting and tenesmus. The diarrheal attack rate was 28.0%. Ingestion of fried bean curd with egg had a significantly high association with the diarrheal attack rate ($p< 0.05$), and the relative risk of the fried bean curd with egg was 10.68 (95% CI=3.88-29.41), as was determined by logistic regression analysis. The bacterial counts in the tap water of the food preparation room and toilet (first floor) exceeded the maximum permissible counts. S.

Enteritidis bacteria were only cultured from the fried bean curd with egg of all the supplied foods on September 3, 2004. CONCLUSIONS: The major cause of salmonellosis was presumed to be the contaminated bean curd via contaminated tap water due to a water leak of a school water pipe. This contaminated bean curd was under prepared, which allowed the S. Enteritidis to survive and multiply prior to its ingestion.

60: J Prev Med Public Health. 2005 Nov;38(4):431-436. Korean.

Seroprevalence of Measles Antibody and its Attributable Factors in Elementary Students of Routine 2-dose Schedule Era with Vaccination Record.

OBJECTIVES: We investigated the seroprevalence of the measles antibody and its attributable factors for the students who underwent routine 2-dose Schedule Era. METHODS: The subjects were 996 students of the national measles seroepidemiologic study in December 2000 who had vaccination records. We conducted a questionnaire survey and we performed serologic testing for the measles specific IgG by using an enzyme linked immunosorbent assay. RESULTS: The coverage for the first dose of the MMR vaccination at 12-15 months of age was 95.1% and the coverage for the second dose of MMR at 4-6 years of age was 35.0%. The proportion of subjects undergoing 2- doses of MMR decreased as the age of the subjects increased. The seropositive rate of the measles antibody was significantly high in the second dose vaccinees (93.5% in the second dose group, 84.7% in the non-second dose group, $p < 0.001$) and it was 72.0% in the 0-dose group,

85.4% in the 1-dose group and 93.7% in the 2-dose group ($p< 0.001$). Two point eight percent of the subjects had a past history of measles infection. On the multiple logistic regression analysis, the first and second dose (odds ratio, 8.54; 95% CI.=3.05-23.91), the first dose (odds ratio, 3.06; 95% CI.=1.20-7.81) and the outbreak in the year 2000 (odds ratio, 1.89; 95% CI.=1.24-2.88) were the significant factors for the eropositivity. CONCLUSIONS: Maintaining high coverage with a 2-dose vaccination program would be the decisive factor to prevent an outbreak of measles and to eliminate measles in Korea.

61: Korean J Epidemiol. 2005 Jun;27(1):173-181. Korean.

Accidents Status of Freshmen for 2 Months before Entrance University.

OBJECTIVES: In recent years, Korea has experienced a loss of manpower and economy due to accidents. The consequences have been comprehensive. Nevertheless, there have been few recent Korean studies on adult accidents. Therefore, this study was carried out to examine the incidence pattern of accidents for 2 months before entering university among university freshmen.
METHODS: The subjects were 1,920 university freshmen who visited the university health center for health examination from March 8 to 12, 2004, and self-reported questionnaire was given to the subjects. The authors developed a questionnaire including age, gender, place and type of high school, experience of accidents, occurrence time and type of accidents and

so on.

RESULTS: The monthly incidence of accident was 9.9 per 1,000 population. The accident cases were 28 in male and 10 in female, and the incidence of males was significantly higher than females. The frequent accident states were 15 cases in 'Ordinary life' (39.8%), 10 in 'Sports activities' (26.3%), 9 in 'Walking' (23.7%) and so on. The accident types were 13 cases of 'Tumbling over' (34.2%), 12 of 'Crash' (31.6%), 8 of 'Traffic accident' (21.0%) and so on. The frequent injury types were 19 cases of 'Sprain' (50.0%), 15 of 'Contusion' (38.4%) and 2 of 'Laceration' (5.3%). One's own carelessness was the most frequent cause of accident (65.8%). After the accident, 28 cases(73.7%) visited the hospital, 7 (18.4%) were hospitalized, and 3 (7.9%) underwent surgery.

CONCLUSIONS: Because the most frequent cause of accidents was one's own carelessness, self-consciousness and attention are needed. The authors expect that this study will go far toward increasing concern of researchers and administrators for the accidents among adults.

62: Korean J Epidemiol. 2005 Jun;27(1):129-139. Korean.

Prevalence and Risk Factors of Green Tobacco Sickness among Korean Tobacco Harvesters, 2003.

OBJECTIVES: This study was carried out to understand the prevalence and risk factors of green tobacco sickness (GTS) among Korean tobacco harvesters.

METHODS: The authors conducted a questionnaire among the tobacco harvesters (842 persons from 449 out of 500 tobacco harvesting households) in Cheongsong-gun for 4 days from Sep 1 to 4, 2003.
RESULTS: The study subjects contained 440 males and 402 females. The prevalence of GTS in 2003 was 39.2%, and was significantly higher in females (53.4%) than in males (25.6%, p<0.01). The incidence density of GTS according to the number of workdays in 2003 was 11.5 spells/100 person-days. The GTS symptoms reported by the tobacco harvesters in 2003 were nausea in 320 cases (97.0%), dizziness in 311 cases (94.2%), headache in 264 cases (80.0%) and vomiting in 209 cases (63.3%) etc. Through multiple logistic regression, the factors significantly associated with GTS were found. Odds ratio for smoking, age over sixty(compared with under forties), and working over 10 hours(compared with under 10 hours) were respectively 0.23 (95% CI: 0.16~0.33), 0.46 (95% CI: 0.23~0.93), and 1.53 (95% CI: 1.12~2.10).
CONCLUSIONS: Our studies before this, there were significant recall bias by time lag between harvesting period and survey time. We tried to study promptly after harvesting tobacco leaves to solve this recall bias. More extensive epidemiologic studies, and educations for harvesters are expected.

63: Korean J Epidemiol. 2005 Jun;27(1):26-36. Korean.

Brucellosis: An Overview.

Brucellosis is zoonotic disease of worldwide distribution and still remains endemic in some developing countries. The main pathogenic species worldwide are B. abortus, responsible for bovine brucellosis, B. melitensis. The B. abortus is most common in Korea. Each Brucella spp. has a preferred natural host that serves as a reservoir of infection. The incubation period varies between 5 and 60 days, and Brucella infection may be asymptomatic or symptomatic. The majority of patients complained of fever (undulating fever), sweats, malaise, anorexia, and arthralgia. The diagnosis of brucellosis requires the isolation of Brucella from blood or body tissues, or the combination of suggestive clinical presentation and positive serology. There were first patients in 2002, thereafter 16 patients in 2003, and 47 patients in 2004, the human brucellosis are increasing more gradually in Korea. Brucellosis is an occupational risk for farmers, veterinarians, and abattoir workers. The main sources of Brucella are infected animals or their products, such as milk, blood, carcasses, and abortion products. Routes of transmission of the infection to humans include direct contact with infected animals and their secretions through cuts and abrasions in the skin, by way of infected aerosols inhaled or via the ingestion of unpasteurized dairy products. A combination of doxycycline and streptomycin has been used widely in brucellosis. Prevention of brucellosis in human still depends on the eradication or control of the disease in animal hosts, the exercise of hygienic precautions to limit exposure to infection through occupational activities and the effective heating of dairy products, and other potentially contaminated foods. Also, physicians and veterinarians must be concerned about specific environments and clinical patterns of brucellosis.

64: Korean J Epidemiol. 2005 Jun;27(1):12-25. Korean.

Anthrax: An Overview.

Human anthrax has been a zoonotic disease affecting those who have close contact with animals or animal products contaminated with the spore-forming bacterium Bacillus anthracis. Now the incidence of anthrax in herbivores and human are rare, but it remains an important health problem in Korea because anthrax is seen as one of the most likely biological weapon. The B. anthracis forms a spore, which is resistant to drought, heat and numerous disinfectants, and the spore can remain viable and infective in the environment for decades. There are three major forms of human disease depending on how infection is contracted, cutaneous, inhalation and ingestion. Inhalational anthrax is the most common form, but the events in the Korea show that gastrointestinal anthrax is the most common. Several cases of anthrax have been reported in Korea. In recent years, 2 cases of bovine anthrax and 5 cases of human anthrax occurred in Changnyeong-gun, 2000, but it haven't occurred any more so far. The most useful microbiological test remains the standard blood culture. Confirmatory diagnostic tests such as polymerase chain reaction can also be used and may help in early diagnosis. Prompt clinical suspicion and rapid administration of effective antimicrobials are essential for treatment of anthrax. Ciprofloxacin or doxycycline should be used for initial intravenous therapy until antimicrobial susceptibility results are known. The best measure to

eliminate human anthrax is control in domestic animals by effective surveillance and by immunization of animals in endemic areas. Also, the government must establish countplan for knowledge and rational policies in dealing with potential bioterrorism attacks.

65: J Prev Med Public Health. 2005 May;38(2):189-196. Korean.

Epidemiologic Investigation on an Outbreak of Shigellosis in Seongju-gun, Korea, 2003.

OBJECTIVES: An outbreak of shigellosis occurred among students and staff of S primary and middle school, Seongju-gun, in 2003. This investigation was carried out to institute an effective counterplan, and study the infection source and transmission of the shigellosis. METHODS: The authors conducted a questionnaire survey among 235 students and staff from S preschool, primary and middle school relating to the ingestion of school lunch and the manifestation of symptoms. Also, the author investigated the drinking water, feeding facility and reconstructed cooking process of the food presumed to be the cause of the shigellosis. The diarrhea cases were defined as confirmed cases and those cases who had had diarrhea more than one time, accompanied with symptoms such as fever, vomiting and tenesmus. RESULTS: From rectal swabs 20 people, between June 28 and July 4, 2003, were confirmed with shigellosis. The diarrhea attack rate was 40.0%. Those who had ingested tomatoes and cubed radish kimchi had significantly higher diarrhea attack rates ($p < 0.05$), with the relative risk of tomatoes being

2.69 (95% CI: 0.98-7.42). The major cause of shigellosis was presumed to be from contaminated tomatoes due to cooking with rubber gloves containing holes. CONCLUSION: The cooks in charge of school lunches must make doubly sure to not only attend to their sanitation, but also to manage the table wear and items used in providing school lunches. The health care authority should introduce higher-leveled criteria for health care among cooks, so that they cannot cook when the have a case of any infectious disease.

66: J Prev Med Public Health. 2005 May;38(2):117-124. Korean.

Changing Patterns of Communicable Diseases in Korea.

Before twentieth centuries and during early twentieth centuries, communicable diseases were the major cause of morbidity and mortality in Korea. But reliable data are not available. After 1975, the overall morbidity and mortality from communicable diseases, rapidly declined. Recently many new pathogenic microbes were recognized: L. monocytogenes, Hantaan virus, Y. pseudotuberculosis, P. multocida, L. pneumophilia, Human immunodeficiency virus (HIV), G. seoi, H. capsulatum, C. burnetii, V. cholerae O139, C. parvum, F. tularensis, E. coli 0157: H7, B. burgdorferi, S. Typhimurium DT104, Rotavirus, hepatitis C virus and so on. Since the first HIV infection recognized in 1985, the reported cases of infection and deaths from HIV/AIDS have been steady increased each year. Legionnaire's

disease, E. coli O157: H7 colitis, listeriosis and crytosporidiasis have been occurring just sporadically among immunocompromized cases. Many re-emerging communicable diseases were occurred in Korea: leptospirosis, malaria, endemic typhus, cholera, tsutsugamushi disease, salmonellosis, hepatitis A, shigellosis, mumps, measles, acute hemorrhagic conjunctivitis, brucellosis and so on. Leptospirosis and tsutsugamushi diseases have been noticed as major public health problems since 1980s. The malaria that had been8 virtually disappeared for a decade has reappeared from 1993 with striking increase of patients in recent 3-4 years. The distributions of salmonella and shigella serotypes have been changed a lot in recent few decades. Furthermore rapid emergence of antibiotic-resistant bacterial strains induces more difficult and complex problems in control of communicable diseases. We must recognize on the importance of environment and ecosystem conservation and careful prescription of anti-microbial agent in order to prevent communicable diseases.

67: J Korean Acad Rehabil Med. 2005 Feb;29(1):63-69. Korean.

Prevalence and Risk Factors of Ulnar Neuropathy at the Elbow in a Rural Population.

OBJECTIVE: The purpose of this study was to investigate the prevalence and risk factors of ulnar neuropathy at the elbow (UNE) in a rural district in Korea. METHOD: Among the 578 residents in a rural district who participated in the health examination, 450 (116 male, 334 female) adults were randomly

selected. A symptom questionnaire and electrodiagnostic studies were used to diagnose UNE. General characteristics, female-related factors, work-related factors and anthropometric measurements were compared between normal and UNE group to identify the risk factors of UNE. RESULTS: Subjects with UNE were 29 (6.4%), symptom without electrodiagnosis findings 23 (5.1%), asymptomatic subjects were 379 (84.2%). Diabetes mellitus, repetitive heavy lifting were risk factors of UNE. CONCLUSION: The prevalence of UNE was 6.4% in a rural district and these data suggest that medical conditions like diabetes mellitus and physical factors like repetitive heavy lifting are risk factors of UNE.

68: Korean J Epidemiol. 2004 Dec;26(2):62-70. Korean.

Status on Influenza Vaccination in Some Community Health Centers.

PURPOSE: This study was conducted to improve the Korean influenza management system and to determine the status of influenza vaccination in some community health centers through a survey of the officers in charge of influenza vaccination.
METHODS: The authors conducted a questionnaire survey by e-mail for the officers in charge of influenza vaccination of 8 community health centers in Daegu-si, 5 community health centers in Ulsan-si and 25 community health centers in Gyeongsangbuk-do. The authors developed a questionnaire including selection methods of the influenza vaccination objects, results of influenza vaccination in 2002, problems of influenza vaccination, the

population within the respondents`jurisdiction and so on.
RESULTS: The proportion of influenza vaccination at community health centers among all residents in the 2002-2003 influenza season was 3.8% in Daegu-si, 14.8% in Ulsan-si, 15.1% in urban Gyeongsangbuk-do and 24.9% in rural Gyeongsangbuk-do. The proportion of influenza vaccination at community health centers among priority cases for vaccination was 12.9% in Daegu-si, 43.1% in Ulsan-si, 39.3% in urban Gyeongsangbuk and 41.6% in rural Gyeongsangbuk-do. The officers in charge of influenza vaccination stated that the problems of influenza vaccination were deficiencies in preparatory examination such as manpower shortage. Twenty-five persons stated as a problem that influenza vaccinations conducted in the hospitals were hardly reported, and fifteen of these clarified that this was due to hospital indifference.
CONCLUSIONS: The authors examined all community health centers of Daegu-si, Ulsan-si, and Gyeongsangbuk-do, and investigated the vaccination states and problems that health officers were dealing with. Therefore, this study is meaningful with its basic data for the management of domestic influenza vaccination.

69: Korean J Occup Environ Med. 2004 Dec;16(4):413-421. Korean.

Urinary Cotinine Concentrations of Cases with Green Tobacco Sickness.

OBJECTIVES: This study was carried out to measure the urinary cotinine concentrations of Korean tobacco harvesters with green tobacco sickness

(GTS).

METHODS: We conducted a questionnaire survey and measured the urinary cotinine concentrations among the cases who visited six health subcenters and the emergency room in the Health Center and County Hospital after harvesting tobacco leaves in Cheongsong-gun, from Jul 15 to Aug 15, 2002. Ten suspected GTS cases were compared to 10 controls matched for residence, age, and sex. Urinary cotinine was analyzed with a reverse-phase high performance liquid chromatography (HPLC) system and expressed as geometric mean and standard deviation. The data collected were evaluated using the SPSS/win statistical package and the urinary cotinine concentrations between the two groups were analyzed by Mann-Whitney U test.

RESULTS: In both the 10 cases and controls, there were 3 males and 7 females. Ages ranged from 22 to 70 years old. Half of the cases were reported within the 6 hours between 12:00 pm and 17:59 pm. The median time from starting work to initially feeling ill was 4.3 hours (min. 2.5; max. 11.0). The GTS symptoms reported were nausea in 10 cases (100.0%), dizziness in 9 (90.0%), weakness in 8 (80.0%), headache in 7 (70.0%) and other symptoms. The geometric mean of urinary cotinine concentrations was significantly higher ($p<0.01$) in cases at 497.6 +/- 2.5 ng/ml (min. 73.1; max. 2,574.3) than in controls at 32.7 +/- 1.8 ng/ml (min. 13.3; max. 76.9).

CONCLUSIONS: Our study suggests that many tobacco harvesters may suffer from GTS in Korea. Therefore, it is very important for doctors to diagnose exactly the GTS. Also we must develop the methods which can prevent GTS, and simple methods of analysis for urinary cotinine.

70: J Korean Med Assoc. 2004 Nov;47(11):1048-1055. Korean.

Brucellosis.

Brucellosis is a systemic bacterial disease with an acute or insidious onset, characterized by continued, intermittent, or irregular fever of variable duration, headache, weakness, sweating, chills, arthralgia, depression, weight loss, and generalized aching. It is predominantly an occupational disease among those working with infected animals or their tissues, especially farm workers, veterinarians, and abattoir workers. Brucellosis has been an emerging disease since the discovery of Brucella melitensis by Bruce in 1887. Worldwide, brucellosis remains a major source of disease in humans and domesticated animals. Although the reported incidence and prevalence of the disease vary widely from country to country, bovine brucellosis caused mainly by B. abortus is still the most widespread form, also occurring in Korea. The vaccination for Brucella was first done on cows in 1998. After inoculation, Brucella developed in a group of cows. And then the first human case occurred in 2002, followed by 16 cases in 2003 by B. abortus. Prevention of brucellosis in humans still depends on the eradication or control of the disease in animal hosts, hygienic precautions to limit the exposure to infection through occupational activities, and effective heating of dairy products or other potentially contaminated foods. Physicians and veterinarians should be concerned about specific environments and clinical patterns of brucellosis. They should make efforts to recognize, evaluate, and control brucellosis.

71: J Prev Med Public Health. 2004 Nov;37(4):312-320. Korean.

Epidemiologic Investigation into the Outbreak of Acute Hemorrhagic Conjunctivitis in Gyeongju-City, South Korea, in 2002.

OBJECTIVES: An outbreak of acute hemorrhagic conjunctivitis (AHC) caused by coxsackie A24 (CA24) virus occurred in South Korea in 2002. CA24 was isolated for the first time from patients with AHC. Therefore, this study was conducted to understand the transmission routes and prevent another AHC outbreaks.

METHODS: The authors conducted a questionnaire survey among 1, 730 students from 2 middle schools and 1 technical high school in Gyeongju city. For statistical analysis the chi-square test was used, and chi-square for trend method showing a level of significance less than $p< 0.05$ was proven to be significant. Variables which were proven to be significant in univariate analysis were analysed by multiple logistic regression.

RESULTS: The attack rate was 57.1%. The student groups with rubbing one's own eyes, computer usage, and sharing cellular phone had a significantly higher AHC attack rate ($p< 0.05$). According to the multiple logistic regression, the odds ratios for male, high school, computer use, sharing cellular phone, and rubbing one's own eyes were significant ($p< 0.05$).

CONCLUSION: The most significant feature of this outbreak was that many students rubbed their own eyes following contact with AHC patients in a deliberate attempt to avoid going to school. Other transmission methods

were computer usage and sharing cellular phone. In the future, health and school authorities must plan new strategies for the prevention of AHC.

72: Korean J Occup Environ Med. 2004 Sep;16(3):336-351. Korean.

Case Series for Occupational Dermatosis in a Factory Treating Liquid Waste Mixtures.

OBJECTIVES: A dermatologic outbreak occurred in a factory usingliquid waste mixtures at Gyeongju. We conducted this study to identify causative factors.
METHODS: We conducted a questionnaire interview composed of general andjob characteristics. A dermatologist carried out medical examination, European standard patch test and 'as is' patch test. We evaluated the cases with over 5 criteria of Mathias's 7 definitions of occupational dermatologic diseases.
RESULTS: Seven of 9 workers were diagnosed with dermatosis and 3 subjects (cases 2, 4 and 6) with Mathias's 7 criteria were confirmed as having occupational dermatologic diseases and were diagnosed as irritant contact dermatitis. Two subjects (cases 1 and 9) with over 5 criteria were evaluated as suspicious status. The remaining 2 subjects (cases 3 and 5) were not compatible with those criteria. The main sources of this outbreak were suggested to be the wasted solvents in the factory of car accessories and aluminum products because the subjects (cases 2, 4, 6, 1 and 9) had the positive skin reaction against those sources in 'as is 'patch test.

Furthermore, 4 of the 5 subjects cases 2, 4, 6 and 9) had some skin reactions against the waste solvents of chemical material in 'as is'patch test.
CONCLUSION: This outbreak occurred by direct skin contact of liquid waste mixtures of a factory making car accessories and aluminum products. The cutting oils and metal working fluids in those liquid waste mixtures were estimated as the most suspected materials. In addition, this outbreak may have been caused by the hazard effect of mixed waste organic solvents of manufacturing of chemical materials.

73: Korean J Occup Environ Med. 2004 Sep;16(3):329-335. Korean.

A Case of Intermediate Syndrome of Organophosphate Poisoning after Dermal Exposure.

BACKGROUND: Organophosphate poisoning is one of the most common toxicologic emergencies in Korea. Acute organophosphate poisoning and delayed polyneuropathy by ingestion are well published. There have been several reports about intermediate syndrome in organophosphate poisoning by ingestion but few about intermediate syndrome via dermal route.
CASE REPORT: We observed a 59-years-old male who had weakness of proximal limb muscles and respiratory muscles 2 days after dermal exposure by unidentified pesticide. The paralytic symptoms lasted up to 20 days but the delayed polyneuropathy did not develop. The patient needed mechanical ventilatory support for 2 weeks and had completely recovered from IMS 6

weeks later. Electrophysiological study was characterized by an axonal polyneuropathy pattern on the proximal limb muscles. Serum acetylcholinesterase level was below half of normal level. Clinical manifestations and electrophysiological study support the clinical diagnosis of intermediate syndrome.
CONCLUSION: Intermediate syndrome is commonly developed by ingestion of organophosphate but, as in this case, dermal absorption can also lead to intermediate syndrome. More detailed history taking and close observation is needed for about 3 or more days after intoxication because of the risk of respiratory failure.

74: J Prev Med Public Health. 2004 Aug;37(3):238-245. Korean.

Smoking Behaviors and Its Relationships with Other Health Behaviors among Medical Students.

OBJECTIVES: This study was conducted to examine the smoking behaviors and the relationship between smoking and other health behaviors among medical students.
METHODS: A self-administrated questionnaire was administered to a sample of 1, 775 students from four medical schools between April and May 2003. Due to the small number of female smokers, the characteristics of smoking behaviors were analyzed only for males.
RESULTS: A total of 1, 367 students (920 males and 447 females) completed the questionnaires, with an overall response rate of 77.7%. The smoking

rates for males and females were 31.5, and 2.2%, respectively. Among the male smokers, 70.7% smoked daily, and 39.0% smoked one pack or more per day. Male students on medical course were more likely to smoke daily, and one pack or more per day, than those on premedical course. Male daily smokers desired to quit smoking less than occasional smokers, and 65.0% of male daily smokers were not ready to quit compared with 37.8% of the occasional smokers. Among the male daily smokers, 29.6% were severely nicotine dependent. The most common reason for not to quit smoking among male smokers was 'no alternative stress coping method' (44.4%), followed by 'lack of will power' (25.4%), and 'no need to quit' (19.4%). Compared with male non-smokers, male smokers were more likely to drink alcohol more often and in larger amounts, take coffee more often, eat breakfast less regularly, and be overweight or obese.

CONCLUSIONS: The results of this study suggest that many male medical students were serious smokers, especially those on medical course. It is necessary to install a smoking prevention program for pre-medical students, provide effective smoking cessation methods for smokers, teach positive stress coping methods, and make the school environment suitable for coping with stress.

75: J Korean Acad Rehabil Med. 2004 Jun;28(3):270-280. Korean.

Prevalence and Risk Factors of the Foot Pain in University Freshmen: One Year Follow-up Study.

OBJECTIVE: The purpose of this study was to investigate the prevalence and risk factors of the foot pain in university freshmen and one year follow-up study. METHOD: The subjects were interviewed about the prevalence and risk factors of the foot pain, which were followed up one year later. RESULTS: In a total of 1, 784 subjects, the prevalence of the foot pain was 22.3% with 17.4% in men and 27.6% in women. The foot pain in the groups was significantly associated with gender, the height of heels, the size of shoes, the recent change of shoes, trauma, change in weight, the shoe type at present and the daily walking distance. There was no significant relation between foot pain and the other parameters, such as body mass index, change of the shoe type or change in height. One hundred and fifty seven subjects were reevaluated after one year. The prevalence of the foot pain was 30.6% with 25.4% in males and 34.9% in females. CONCLUSION: The prevalence of the foot pain was 22.3% in university freshmen and 30.6% in one year follow up study, and was significantly associated with shoe problems and lifestyles.

76: Korean J Epidemiol. 2004 Jun;26(1):59-68. Korean.

Epidemiologic Investigation into an Outbreak of Typhoid Fever Recognized by Electronic Data Interchange in Gyeongsangbuk-do, 2003.

Epidemiologic Investigation into an Outbreak of Typhoid Fever Recognized by Electronic Data Interchange in Gyeongsangbuk-do, 2003

PURPOSE: Fourteen cases of typhoid fever occurred in Gyeongsan-si,

Gyeongsangbuk-do from May 22 to July 19, 2003. Especially, 6 cases were students of Y University located in Gyeongsan-si. This investigation was carried out to study the infection source and transmission of typhoid fever and to institute an effective prevention counterplan.

METHODS: The authors conducted an epidemiologic survey from June 25, 2003 among the 14 cases, restaurants and employees of the whole neighborhood of Y University. Widal test was carried out for the restaurant employees and schoolmates of the 13-year-old case Miss Kim. The authors executed a pulsed field gel electrophoresis (PFGE) for Salmonella typhi cultured from 9 cases. The authors collected data about chloride and turbid levels of treated water from

April to June in Gyeongsan-si water treatment plant and repair work records of waterworks pipe line in Gyeongsan-si city hall.

RESULTS: All cases had used restaurants of the whole neighborhood of Y University within 2 months before the typhoid fever symptoms were developed, but the restaurant that was rarely visited by any of the patients had a case to be matched. In the results of the Widal test, 9 out of 45 of subjects (20.0%) were suspicious cases or showed a value to suggest past infection. In the results of PFGE test, there were 3 subtypes, and each subtype had a similar DNA fragments array. In all cases the patients drank tap water when they visited the restaurants. The restaurants provided drinking water from filtered tap water rather than boiled water, and all patients had drunk this water. The chlorine level of the treated water was the normal value in the Gyeongsan-si water treatment plant, but in 2 times the value was the minimum standard level. There was water pipe construction work in some regions of the whole neighborhood of Y

University. The number of gastrointestinal patients in 2003 was approximately 2 times that in 2002 at the health center in Y University.
CONCLUSIONS: The possibility that typhoid fever was generated in the whole neighborhood of Y University is very high. Among various possibilities, the highest possibility is the pollution of tap water intake by water leakage of a water pipe. There was no case of patient infection after strengthening of the tap water disinfection. Thorough disinfection, boiled drinking water and a periodic carrier inspection are necessary to prevent additional local occurrences of these typhoid infections.

77: Korean J Epidemiol. 2004 Jun;26(1):39-49. Korean.

Epidemiological Characteristics and Changes of Prevalence for Green Tobacco Sickness among Korean Tobacco Harvesters.

PURPOSE: This study was carried out to investigate the epidemiological characteristics and changes of prevalence for green tobacco sickness (GTS) for 2 years.
METHODS: The author conducted a questionnaire survey on the tobacco harvesters (875 persons from 478 out of 555 tobacco harvesting households surveyed last year) in Cheongsong-gun for 4 days from May 26 to 29, 2003.
RESULTS: The study subjects comprised 456 males and 419 females. The prevalence of GTS in 2002 was 50.5%, which was significantly higher than the 43.0% in 2001 ($p<0.01$). The incidence density of GTS in 2002 was 11.3 spells/100 person?working-days, which was lower than the 12.1 spells in

2001. Regardless of risk factors such as smoking, workingdays, and working hours, the prevalence of GTS in 2002 was higher than that in 2001. Among various GTS symptoms reported by the tobacco harvesters in 2002, cases of nausea were increased, while diarrhea, dyspnea and cough were decreased compared with 2001. The degree of GTS symptoms in 2002 was increased in 198 cases (64.5%), and decreased in 42 cases (13.7%). The proportion of harvesters who underwent treatment from their local medical facilities in 2002 was significantly decreased compared with that in 2001.
CONCLUSIONS: In Korea, there are many tobacco-harvesting households, most of which may be stricken with GTS. It is very important for doctors to diagnose the disease exactly and to develop prevention methods for GTS. I expect that more extensive epidemiological studies including the incidence and associated risk factors will be needed. In addition, surveillance system and measurements of urinary cotinine should be conducted.

78: Korean J Epidemiol. 2004 Jun;26(1):1-16. Korean.

Analysis of Transmission Mode of Confirmed Shigellosis in Gyeongju, Korea.

PURPOSE: This study was carried out to investigate the sources of infection and modes of transmission of an 107 (54males, 53 females) confirmed shigellosis in Gyeongju from Sept. 24 to Oct. 24, 1998.
METHODS: We reviewed hospital records of all confirmed shigellosis. Thirty-two strains of Shigella sonnei isolated were analyzed in order to trace the source of infections by plasmid profile, antimicrobial drug

resistance pattern, biotyping and pulsed-field gel electrophoresis (PFGE). RESULTS: The first source of infection was estimated to be contaminated underground water and simple piped water caused by leakage from the cess pool. Antimicrobial drug resistance pattern showed that all strains were multi-resistant. All isolates had the same XbaI and SfiI PFGE patterns, indicating the epidemiological relationship of the outbreaks strains in this area. The major characteristics of diarrhea were watery (84.2%) in nature. The clinical symptoms among the diarrheal cases included abdominal pain (86.1%), fever (83.2%), headache (78.2%), chill (45.5%) and tenesmus (41.6%). Six cases were asymptomatic. From initial manifestation to admission were 6.2+/-1.6 (median 6) days, so it could be transmitted to others in this infective period. Duration of admission were 5.5 +/-2.4 (median 6) days. We can find familial mean secondary attack rate were 38.5%. CONCLUSION: It's transmission to other areas were certified by contaminated water and contact with shigellosis, and unknown cases were estimated to be transmitted by contacts with inapparent infection. By plasmid profile and PFGE, the epidemiological relationship of the outbreaks strains in this area seemed to be the same micro-organisms.

79: Korean J Occup Environ Med. 2004 Mar;16(1):25-36. Korean.

A Study on the Occupational Hazards Associated with Chronic Renal Failure.

OBJECTIVE: Recently, chronic renal failure has become a major public health

problem all around the world, and the number of patients in Korea affected by this disease has sharply increased since the 1980's. Between 3% and 50% of all cases of chronic renal failure may be induced by toxic agents. The purpose of our study was to investigate the occupational hazards associated with chronic renal failure, in order to use the findings as a reference for further epidemiologic studies.

METHODS: The author conducted a questionnaire and reviewed the medical records of chronic renal failure patients (161 people out of 238) in Gyeongju-si and Pohang-si from March 2 to 31, 2003. Those cases of chronic renal failure which were related to work were classified as either 'probable case' if they were of unknown origin or 'suspected case' if they were of known origin and were related to hypertension or diabetes mellitus.

RESULTS: The study subjects consisted of 92 males (57.1%) and 69 females (42.9%). The causalities listed in the medical records were diabetes mellitus in 55 cases (40.4%), hypertension in 28 cases (20.6%), chronic glomerulonephritis in 14 cases (10.3%), and other diseases. There were 10 cases (6.2%) that were believed to be related to the patients' occupations. The hazards that these 10 patients had previously been exposed to were silica, organic solvents, tin, copper, lead, cadmium, and other hazards.

CONCLUSIONS: Through this study, 10 cases that were reportedly related to occupational chronic renal failure were discovered. Further studies such as a case-control study of occupational risk factors related to chronic renal failure will be needed.

80: Korean J Prev Med. 2004 Feb;37(1):80-87. Korean.

Evaluation of Field Epidemiology Specialist Training Program Based on the Satisfaction and the Changes of Educational Needs.

OBJECTIVE: To evaluate the learning achievement and satisfaction levels for the Field Epidemiology Specialist Training Program (FESTP), on infectious disease control between March 19 and October 31, 2002.

METHODS: The FESTP was designed as a set of 84 hours curricula including lectures, discussions, self-studies, and field practicals, and organized both centrally and locally by the Division of Communicable Disease Control of the National Institute of Health and 11 universities. Before and after the program, a questionnaire survey on the educational need (49 items) and satisfaction (15 items) was conducted on 484 trainees, who were responsible for communicable disease control and immunization at 242 regional health centers. The data were analyzed with paired t-tests for comparison of the educational needs between the pre and post scores.

RESULTS: The average score for satisfaction was 3.06 out of 5.0; with relatively higher scores for sincerity (4.10) and professionalism (4.01) of the tutors, adequacy (3.54) and clearness (3.51) of the evaluation criteria, usefulness (3.54) and fitness (3.52) of the contents, but with relatively lower satisfaction for schedule (2.96) and self-studies (2.91). The average for requirement for education improved, as shown by the decrease from 2.72 to 2.22 ($p < .0001$) with the biggest decrease in the outbreak investigation from 2.60 to 2.08.

CONCLUSION: The FESTP was evaluated as being effective, the trainees

showed moderate satisfaction and decrease educational needs. However, the actual schedules and self-studies should be rearranged to improve the satisfaction level.

81: Korean J Prev Med. 2004 Feb;37(1):37-43. Korean.

Prevalence and Risk Factors of Green Tobacco Sickness among Korean Tobacco Harvesters.

OBJECTIVE: This study was carried out to understand the prevalence and risk factors of green tobacco sickness (GTS) among Korean tobacco harvesters.
METHODS: The authors conducted a questionnaire among the tobacco harvesters (1, 064 persons from 555 out of 723 tobacco harvesting households) in Cheongsong-gun for 4 days from May 7 to 10, 2002.
RESULTS: The study subjects were 550 males and 514 females. The recognition and experience of GTS up until 2001 were 96.4% and 61.9%, respectively. The prevalence of GTS in 2001 was 42.5%, and was significantly higher in females than in males (59.0% vs. 26.6%, $p < 0.01$). The incidence density of GTS according to the number of workdays in 2001 was 12.3 spells/100 person' days. The GTS symptoms reported by the tobacco harvesters in 2001 were dizziness in 441 cases (97.6%), nausea in 414 (91.6%), headache in 349 (77.2%) and vomiting in 343 (75.9%). The use of gloves, hat and wristlets, sweating at work and the number of working hours significantly increased the prevalence of GTS ($p < 0.05$). Multiple logistic regression analysis was used to determine the factors significantly

associated with GTS. Odds ratios for smoking, working over 10 hours and sweating at work were 0.26 (95% CI: 0.19-0.35), 1.64 (95% CI: 1.26-2.14) and 1.60 (95% CI: 1.14-2.25), respectively. Of those who reported GTS in 2001, 311 cases (68.8%) underwent treatment from their local medical facilities.

CONCLUSION: In Korea, there are many tobaccoharvesting households, and most may be stricken with GTS. More extensive epidemiological studies, including heincidence and associated risk factors, are expected and a surveillance system including measurements of cotinine in urine should be conducted.

82: J Korean Med Assoc. 2004 Jan;47(1):75-82. Korean.

Guidelines for Work-related Diseases: Health Care Worker's Standpoint.

The major occupational diseases among health care workers in Korea include infectious, cerebrocardiovascular, and musculoskeletaf diseases. Infectious diseases such as tuberculosis, viral hepatitis, and chicken pox would be compensated as occupational diseases among health care workers. Also cerebro cardiovascular diseases would be compensated if they had overworked. Many other diseases would be compensated if they had workrelatedness. The decision of the person responsible for determining the workrelatedness of a disease must be based on an evaluation of the available information. Evidence presented by qualified professionals will generally be sufficient to answer the following questions: (1) has the

disease condition been clearly established? (2) has it been shown that the disease can result from the suspected agent (s)? (3) has exposure to the agent been demonstrated? (4) has exposure to the agent been shown to be of a sufficient degree and/or duration to result in the disease condition? (5) has non-occupational exposure to the agent been ruled out as a causative factor? have all special circumstances been weighed? (7) has the burden of proof of causation been metdid the evidence prove that the disease resulted from, or was aggravated by, conditions at work? If the answers to all of the above are "Yes", the decision can be made that the disease is occupational in origin. Occupational safety and health programs are not yet sufficient in Korea. Occupational safety and health programs are very important to health care workers, and so special management, policies, and laws relevant to these issues should be promulgated.

83: Korean J Epidemiol. 2003 Dec;25(2):47-61. Korean.

Status on Occurrence and Preventive Measures of Disasters in Korea.

Disasters are emergencies of a severity and magnitude resulting in deaths, injuries, illness and/or property damage that cannot be effectively managed by the application of routine procedures or resources. These events are caused by nature, the result of technological or manmade error. Natural disasters include typhoon, flood, heavy snowfall, drought, famine, and earthquake. Natural disasters are unpreventable and, for the most part, uncontrollable. Technological or manmade disasters include fire, nuclear

accidents, bombings, and bioterrorism.
The severity of damage caused by natural or technological disasters is affected by population density in disaster-prone areas, local building codes, community preparedness, and the use of public safety announcements and education on how to respond correctly at the first signs of danger. Recovery following a disaster varies according to the public's access to pertinent information, pre-existing conditions that increase or reduce vulnerability, prior experience with stressful situations, and availability of sufficient savings and insurance.
Epidemiology can be used to investigate the public health and medical consequences of disasters. The aim of disaster epidemiology is to ascertain strategies for the prevention of both acute and chronic health events. Disaster epidemiology includes rapid needs assessment, disease control strategies, assessment of the availability and use of health services, surveillance systems for both descriptive and analytic investigations of disease and injury, and research on risk factors contributing to disease, injury, or death. With both disasters and the number of people affected by such events on the increase, the importance of disasters as a public health program is now widely recognized in Korea. The epidemiologists must do their best effort for prevention of disasters.

84: Korean J Occup Environ Med. 2003 Dec;15(4):351-363. Korean.

Status of the Occupational Health and Safety Programs of Several Hospitals in Korea.

OBJECTIVES: As health care workers suffer from various hazards in hospital, an occupational safety and health program is very important to them. This study was conducted to investigate the status of the occupational safety and health programs of several hospitals in Korea.
METHODS: We conducted a questionnaire survey on 941 hospitals in Korea. All questionnaires were posted to the department of general affairs at each hospital and responses were received from 108 hospitals (11.5%). Eight responses were excluded because of insufficient data and the remaining 100 questionnaires were analyzed. The collected data were evaluated using the SPSS 10.0 statistical package and the demands for occupational safety and health programs between two groups were analyzed by RIDIT analysis.
RESULTS: The study subjects were 53 general hospitals and 47 hospitals. An occupational safety and health committee, infection control committee, safety manager, health manager, activities for vaccination, reports for needle stick injury and records related to occupational injury were more likely to be present in general hospitals than hospitals ($p<0.05$). Demands for occupational safety and health were higher in general hospitals than hospitals. Especially, demands for measurement of working environment, improvement of ventilatory system, management of chemical materials and supply of protective equipment were significantly higher in general hospitals than hospitals ($p<0.05$).
CONCLUSIONS: We concluded from the findings of our study that occupational safety and health programs are not yet sufficient in Korean hospitals. So, we expect that special management, policies and laws for health care workers will be promulgated.

85: Korean J Prev Med. 2003 Aug;36(3):223-229. Korean.

Survey on the Symptoms Related to Hair Dyeing among University Freshmen.

OBJECTIVES: This study was carried out to investigate the prevalence and symptoms related to hair dyeing among university freshmen.

METHODS: The authors conducted a questionnaire survey among 1, 499 university freshmen from Mar 11 to 15, 2002.

RESULTS: The study group contained 710 females and 789 males. Up until 2002, 62.7% of the subjects had experienced hair dyeing, and this was significantly higher in females (66.2%, $p < 0.05$). The period of first experience of hair dyeing was in high school for 361 cases (38.4%), after high school for 345 cases (36.7%) and before high school for 234 cases (24.9%). The major reasons of hair dyeing were 'to improve their appearance' in 466 cases (49.6%), and 'to follow the hair dyeing fashion' in 169 cases (18.0%). The prevalence of hair dyeing in 2002 was 47.8%, and again was significantly higher in females (53.7%, $p < 0.05$). The major symptoms related to hair dyeing were 'cleaved and nonelastic hair' in 498 cases (69.6%), and 'thin and easily breakable hair' in 353 cases (49.3%).
Of those, 361 cases (50.4%) appealed to three or more symptoms related to hair dyeing. Through multiple logistic regression, factors significantly associated with symptoms related to hair dyeing were found to be female (OR=2.14, 95% CI; 1.61-2.83), use of hair dryer (OR=1.36, 95% CI; 1.004-1.854), a frequency of hair dyeing of three or more (OR=1.48, 95% CI;

1.04-2.09), and a duration of processing hair dyeing of over 60 minutes
(OR=2.18, 95% CI; 1.50-3.18).
CONCLUSIONS: The prevalence and experience of hair dyeing were generally high among university freshmen. Therefore, more extensive epidemiological studies on the symptoms related to hair dyeing should be conducted.

86: J Korean Med Sci. 2003 Jun;18(3):429-432. English.

A Case of Gastroenteritis Associated with Gastric Trichuriasis.

A rare human case of gastroenteritis and eosinophilic ascites associated with gastric trichuriasis is described. The patient was a 32-yr-old woman who was working in a farm near Pohang, Korea. She complained of abdominal pain, diarrhea, and vomiting. Endoscopic examination found focal linear hyperemia on the mucosa of the stomach antrum, and endoscopic biopsy confirmed eosinophilic inflammation of the mucosa and submucosa of the stomach, terminal ileum, and cecum. The biopsy specimen of the stomach included a female Trichuris trichiura which was covered by many inflammatory cells on its surface. Ascites and intestinal wall thickening was found by CT scan, and Douglas pouch centesis aspirated bloody ascites which included many eosinophils. She was medicated with prednisolone and albendazole and cured. She is the first case of eosinophilic inflammation of the gastrointestinal tract and ascites associated with trichuriasis in the stomach.

87: Korean J Occup Environ Med. 2003 Jun;15(2):196-204. Korean.

Occupational Diseases on Health Care workers Approved by Korea Labor Welfare Corporation.

OBJECTIVES: This study was carried out to analyze the characteristics of occupational diseases among health care workers in Korea.
METHODS: Using the database of the Korea Labor Welfare Corporation (KLWC), 5, 460 approved occupational disease cases, between Jan 1, 1999 and Dec 31, 2000, and approved by the Jun 30, 2001, were collected. Of these, the 142 health care worker cases were investigated.
RESULTS: The proportion of health care workers among the total cases reported in 1999 and 2000 was 2.6%, respectively. The diseases were infectious in 61 (43.0%), cerebro-cardiovascular in 60 (42.3%) and musculoskeletal in 18 cases (12.6%). The distributions of the occupational diseases by sex were 59 (41.5%) and 83 cases (58.5%), in males and females, respectively. The distributions of the occupational diseases by age were 64 (45.1%), 30 (21.1%), 25 (17.6%) and 17 cases (12.0%), in their 20s, 30s, 40s and 50s, respectively. For the cases of infectious diseases by age there were 48 (78.7%) in their 20s and 12 (19.7%) in their 30s, for the cases of cerebro-cardiovascular diseases by age there were 17 (28.3%) in their 40s, 16 (26.7%) in their 50s and 12 (20.0%) in their 20s, and for the cases of musculoskeletal diseases there were 7 (38.9%) in their 30s and 4 (22.2%) in their 20s. The distributions of the cases of occupational diseases by department were 64 (45.1%), 19 (13.4%), 18 (12.7%), and 10

(7.0%) in the nursing, doctors, officers, and food services departments, respectively.
CONCLUSIONS: The major occupational diseases of health care workers, as approved by the KLWC, were the infectious, cerebro-cardiovascular and musculoskeletal diseases. A special management policy must be considered to prevent occupational diseases of health care workers in Korea.

88: Korean J Prev Med. 2003 Feb;36(1):77-84. Korean.

Cause of Enterohemorrhagic Escherichia coli Infection in Ulju County, Korea.

OBJECTIVES: Two related cases of Hemolytic-Uremic Syndrome (HUS) were reported to the Korea National Institute of Health in May, 2001. Shiga toxin 2 genes were detected in both stool samples. We suspected an enterohemorrhagic Escherichia coli (EHEC) infection as the cause of the HUS, and conducted an investigation to find the source of the infection and its route of transmission.
METHODS: We performed case investigations on these two related HUS cases, and obtained interviews and rectal swabs form the family members and other close contacts. Additionally, we performed rectal swabs on the cattle raised by the household of the index patient.
RESULTS: We found a 20 month old index patient and a 6 year-old cousin had developed HUS, where there had been a 2 day history of contact with the index, and bacteriological examinations for these two patients revealed, indistinguishably, the same E. coli O171. The grandmother of the index

patient was found to be asymptomatic, but E. coli O26 was isolated. We also found a probable case in the mother of the cousin. She reported a history of contact with the index, and developed bloody diarrhea of 3 days duration. The test results for the cattle revealed E. coli O26 in one cow, and E. coli O26 and O55 in another. E. coli O26, which was isolated in both cows and the grandmother of the index, were indistinguishably the same.
CONCLUSIONS: We found that the E. coli O26 in the grandmother had originated from the cows, and that the E. coli O171 found in the index patient had been transmitted to the cousin through person-to-person contact.

89: Korean J Anesthesiol. 2002 Nov;43(5):558-565. Korean.

A Study on Analgesic Effect of Metoclopramide before an Injection of Propofol.

BACKGROUND: Propofol is a good induction agent. but it has a disadvantage of pain on intravenous injection. Pretreatment of metoclopramide or lidocaine have been reported to reduce pain on injection. thus, we have evaluated the quantity and quality of anagesic effect of metoclopramide and lidocaine. We observed differences in quality of pain according to venous cannula sizes and intravenous injection sites as well as nausea and vomiting in the postoperative state.
METHODS: Eighty patients scheduled for an elective operation by general anesthesia were chosen according to ASA (I or II) and divided into four

groups randomly. Each group was injected through venous cannulas with normal saline (control group), metoclopramide 5 mg (group 1), metoclopramide 10 mg (group 2), or 2% lidocaine 40 mg (group 3) respectively. Then, propofol was injected of a 2 mg/kg dose with 0.5 ml/sec to all groups and we asked questions about injection pain after 10 seconds.

RESULTS: Pain relief was shown in all groups compared with the control. but metoclopramide 10 mg and lidocaine 40 mg pretreatment groups showed significant pain reief. Pain was relieved significantly when the drug was injected in the antecubital area. Postoperative nausea and vomiting were not observed.

CONCLUSIONS: Metoclopramide 10 mg or lidocaine 40 mg pretreatment to induction by propofol revealed a good analgesic effect for propofol injection pain.

90: Korean J Prev Med. 2002 Nov;35(4):295-304. Korean.

Epidemiologic Investigation on an Outbreak of Cholera in Gyeongsangbuk-do, Korea, 2001.

OBJECTIVES: This study was carried out to investigate the cause, magnitude and transmission route of the cholera outbreak in 2001.

METHODS: The study population were those persons who ingested foods at the restaurant, were confirmed as cholera patients, had symptoms of diarrhea and served as workers at the restaurant. A questionnaire survey and

microbiological examinations on the microbes isolated from rectal swabs were conducted. Of the cases, 316 food histories were surveyed by an analysis of the restaurant menu.
RESUJLTS: There were 139 confirmed cases of cholera reported in Korea in 2001. Of these, 104 were related to the restaurant. By region, Gyeongsangbuk-do had the highest incidence with 91 cases. Of these 91 cases, 74 had ingested foods at the restaurant, 2 were employees and 3 were secondary infection cases within the families. The results of the odds ratio analysis of the 316 persons having ingested foods at the restaurant were as follows: sandwiches 5.07 (95% CI, 1.85-14.59), soybean curd 2.45 (95% CI, 1.09-5.56), noodles 2.34 (95% CI, 1.24-4.42), steamed squid 2.01 (95% CI, 1.17-3.47) and vinegared rice 1.82 (95% CI, 1.08-3.09). It was certain the restaurant in question was the cause of the 2001 outbreak.
CONCLUSIONS: We suspected that more than one restaurant employee contaminated foods served at the restaurant. In addition, eating raw fishes purchased at the Pohang Fisheries infected the employees of the restaurant. There is a possibility that these raw fishes were themselves contaminated by cholera bacilli in the sea.

91: Korean J Occup Environ Med. 2002 Sep;14(3):213-226. Korean.

Carpal Tunnel Configuration Measured by Ultrasonography as a Risk Factor of Carpal Tunnel Syndrome in Motor Part Manufacturing Workers.

OBJECTIVES: This study was conducted to evaluate individual susceptibility

to carpal tunnel syndrome (CTS) by ultrasonographic measurement of the carpal tunnel configuration in workers doing repetitive work.

METHODS: The study subjects consisted of 24 male and 11 female workers in a soundproof material manufacturing company in Gyeongju. We conducted a self-reported questionnaire survey, a physical examination and an electrodiagnostic study (EDS) in April 2000. After the examination, jobs were rearranged for workers with CTS. A follow up physical examination, EDS, and measurement of the carpal tunnel by ultrasonography was done six months later.

RESULTS: Of those studied, prevalence of CTS was 63.6/100 persons among women and 29.2/100 persons among men. Mean depth and width of wrist was shorter in those with CTS compareal to the controls ($p<0.05$). The risk of CTS was higher in workers whose carpal tunnel ratio (displacement/width) was 0.17 or higher (OR 7.13, 95 % confidence interval 1.18-43.1), and in workers whose carpal tunnel area was less than 300 mm2 (OR 8.00, 95 % confidence interval 1.18-68.5). Carpal tunnel depth and depth/width ratio had a positive correlation with motor latency of the median nerve and median-ulnar sensory latency difference ($p<0.05$), whereas the carpal tunnel width, displacement/depth ratio, and area (width X displacement) had a negative correlation with median nerve latencies after adjusting for gender. Workers who showed an improvement in the clinical stage of CTS after job rearrangement had a significantly lower carpal tunnel displacement/width ratio and displacement/depth ratio. They also had smaller upper carpal tunnel area (width X displacement) and larger upper carpal tunnel area [(depth-displacement) X width] than the controls ($p< 0.05$).

CONCLUSIONS: Ultrasonographic measurement of the carpal tunnel is a good predictor of susceptibility and prognosis of CTS in workers doing repetivive work.

92: J Korean Med Assoc. 2002 Aug;45(8):1027-1035. Korean.

Diagnosis and Management of Green Tobacco Sickness.

Nicotine is a liquid alkaloid present in tobacco leaves at a 1~6% concentration. Green tobacco sickness is an occupational illness caused by absorption of nicotine through skin exposed to wet tobacco leaves. It occurs throughout the world in tobacco-growing areas. The almost universal symptoms of weakness, nausea, vomiting, diarrhea, and dizziness may be confused with pesticide poisoning or heat stress illness. Since its recognition in the 1970s, green tobacco sickness is self-limited, usually lasting 1 to 2 days. There is no antidote, so treatment is supportive (for example, intravenous fluids for hypotension, antiemetics). The diagnosis can beconfirmed by measuring the nicotine concentration in the serum or urine, but its half-life is only 3~4 hours. Cotinine, the major nicotine metabolite, has a half-life of 36 hours, and thus is more useful. Young workers may be at higher risk for green tobacco sickness. Tobacco use is thought to offer weak protection by inducing tolerance. Handling tobacco that is wet from rain or dew increase the risk of green tobacco sickness. Repeated daily exposure over more than a week may also increase the risk. Green tobacco sickness is preventable by use of protective clothing and by

avoiding skin contact with wet tobacco. When clothing becomes wet from environmental dampness or sweating, it should be changed to maintain an effective barrier. The best treatment is avoidance of poisoning : in addition to wearing gloves, long pants and a full shirt, workers picking or handling tobacco leaves should clean their hands frequently.

93: J Korean Med Assoc. 2002 Jun;45(6):741-749. Korean.

Karoshi : Death from Overwork.

The first case of karoshi was reported in 1969 with the death from a stroke of a 29-year-old, married male worker in the shipping department of Japan's largest newspaper company. It was initially called occupational sudden death. Shift work and an increased work load, together with excessive overwork in spite of ill health just before the stroke, were finally recognized as the occupational causes of death. It took five years for the family to receive compensation. In 1982, the first book entitled "Karoshi" was published by three physicians. This was the origin of the term karoshi. Karoshi is not a pure medical term but a sociomedical term. Karoshi-deaths were associated with long working hours, shift work, stress, and irregular work schedules.

In Korea, karoshi was introduced in 1990. The cases with cerebral hemorrhage, subarachnoid cerebral hemorrhage, cerebral infarction, hypertensive encephalopathy, angina pectoris, myocardial infarction, and dissecting aneurysm would be compensated as occupational diseases if the

patients had overworked. Now, the magnitude and kinds of diseases of karoshi is being extended. Medical doctors must understand the karoshi and make efforts to make the victims of karoshi be compensated.
The evidence that overwork causes sudden death is still incomplete. More studies are needed to clarify the causal relationship. To prevent karoshi, the working hours should be shortened and health promotion programs for all workers should be encouraged.

94: Korean J Epidemiol. 2002 Jun;24(1):54-62. Korean.

Outbreak of Salmonellosis Misdiagnosed with Amebiasis in Gumi City and Chilgok County, Korea.

PURPOSES: In May 29, 1999, the health department in Gumi city received a report from a local pediatrician that three children who attended a kindergarten were diagnosed with amebic dysentery. By May 31, fifteen more children from the same kindergarten were diagnosed with amebic dysentery. We conducted an investigation in order to verify the diagnosis, and to implement appropriate control measures.
METHODS: We conducted a questionnaire survey on 264 children in 3 kindergarten in Gumi city. Furthermore, 726 children in 4 kindergarten and 13 academies in Chilgok county whose lunch is supplied by the same unlicensed catering company were
monitored for diarrheal symptoms.
RESULTS: Of 264 children in Gumi city, 74 children fitted the case

definition (attack rate, 28%). Of 726 children in Chilgok county, 50 children were reported to have diarrheal symptoms. The clinical picture was dominated by the following symptoms; abdominal pain (85.1%), fever (83.8%), headache (50.0%), chilling (45.9%), vomiting (28.4%). The median duration of diarrhea was 2 days, and the median frequency of diarrhea was 3 times/day. Salmonella Typhimurium of the same antibiogram pattern were isolated from fifteen cases. However, no evidence of amebiasis was found from laboratory results or epidemiologic pattern.
CONCLUSIONS : This epidemic was caused by Salmonella Typhimurium, which were present in lunch supplied by the unlicensed catering company. Improvement of the diagnostic ability in local health centers as well as public health centers and
reinforcement of strict protocols regarding appropriate management of catering services should be emphasized

95: Korean J Epidemiol. 2002 Jun;24(1):29-36. Korean.

Green Tobacco Sickness on Tobacco Harvesters in a Korean Village.

PURPOSE: This study was conducted to understand the proportion and the risk factors of green tobacco sickness (GTS).
METHODS: The authors conducted a questionnaire among tobacco harvesters; 94 people from 59 households at a village in Youngduk-gun from Feb 1 to Feb 3, 2002.
RESULTS: The study group contained 49 females and 45 males. The mean

durations of employment and the length of the working day were 23.3 14.2 years and 11.8 2.7 hours, respectively. The symptoms related to tobacco harvest were dizziness in 64 cases (68.1%), headache in 46 cases (48.9%), nausea in 45 cases (47.9%), sore eyes in 37 cases (39.4%), and vomiting in 36 cases (38.3%). The experience of GTS up until 2001 was 70.2%. The proportion of GTS in 2001 was 67.0% and was significantly higher in females (84.9%, $p<0.01$). The proportion of GTS by work days in 2001 was 16.4 spells/100 person days. The proportion of non-smokers was significantly higher than smokers ($p<0.01$). Therefore, smoking was negatively associated with GTS. The use of gloves and wristlets significantly increased the proportion of GTS ($p<0.05$). Through multiple logistic regression, significantly associated factors with GTS were found to be smoking (OR=0.18, 95% CI: 0.06-0.54), wearing of gloves (OR=9.20, 95% CI: 1.27-66.52), and sweating (OR= 3.52, 95% CI: 1.08-11.47). Of those who reported GTS in 2001, 65.6% underwent treatment from the local medical facilities. The distribution of the medical facilities utilized was pharmacies 39.3%, and medical health centers 26.2%.

CONCLUSIONS : In Korea, there are many tobacco harvesters, and most may be stricken with green tobacco sickness. In the future it is hoped that more extensive epidemiological studies will be conducted

96: Korean J Occup Environ Med. 2002 Jun;14(2):204-212. Korean.

Cold Sensitization Occurring in a Worker of a Cold Storage Warehouse.

OBJECTIVES: The following is a report on a case of cold sensitization in a worker who served as a forklift truck driver in a cold storage warehouse for 5 years from 1996.
METHODS: We examined the patient's blood chemistry and infrared computerized thermographic measurements. We also reviewed the environmental temperatures at his workplace and interviewed other workers.
RESULTS: The worker was a 32-year-old male who had worked in a cold storage warehouse for 5 years (1996-2000). He complained of headaches, arthralgia, and slight dyspnea after 2 years work serving as a forklift truck driver in a cold storage facility. In the laboratory record of his blood and those of others, the C-reactive protein, rheumatoid facror, anti-nuclear antibody, cryoglobulin and other blood chemistries were all negative except that the anti-streptolysin O titer was reactive and alkaline phosphatase was also increased. The radiologic findings were normal. Using infrared computerized thermographic measurements, the patient's hands were at around 30 degrees C and below in the first infrared image and around 27 degrees C in the third image. Accordingly, he was diagnosed as having cold sensitization, which occurs in persons exposed to cold for long time, especially below freezing point.
CONCLUSIONS: We confirmed that the cold sensitization occurred in this patient as an occupational disease due to an exposure to cold. To prevent cold injury, we must modify work procedures, begin comprehensive medical surveillances, educate workers on risks of cold, and increase workers awareness of safety regulations in the workplace.

97: Korean J Occup Environ Med. 2002 Mar;14(1):97-106. Korean.

Lung Cancer Occurring in a Worker Exposed to Coke Oven Emissions.

OBJECTIVES: We report a case of lung cancer (small cell carcinoma) occurring in a worker exposed to coke oven emissions.
METHODS: We examined the chest CT and pathologic findings of the patient. We reviewed previous environmental measurements for coke oven emissions at the workers place of employment. Also we measured the airborne concentrations for coke oven emissions and total polycyclic aromatic hydrocarbons in the work area. Finally, we analyzed the 1-hydroxypyrene and 2-naphthol concentrations in the subjects urine and compared this with student controls.
RESULTS: This case was a 56-year-old male who had worked in a coke oven plant within a steel manufacturing factory for 21 years (1977-1998). The airborne concentrations of coke oven emissions at the worksite were above the permissible exposure level (0.2 mg/m3) in 45 cases (33.1%) among 136 workers. The concentrations of 1-hydroxypyrene and 2-naphthol in the subjects urine were statistically significantly elevated as compared with those of controls ($P<0.01$).
CONCLUSIONS: We confirmed that the lung cancer occurring in this patient was as an occupational disease due to exposure to coke oven emissions. To prevent occupational cancer in coke oven plant workers, we must remodel the engineering procedure, begin comprehensive medical surveillance, educate workers on risks and the benefits of smoking cessation, and increase awareness of safety regulations in the workplace.

98: J Korean Acad Rehabil Med. 2002 Feb;26(1):26-31. Korean.

Musculoskeletal Pain in Preadolescent Children.

OBJECTIVE: To investigate the prevalence and contributing factor of musculoskeletal pain in preadolescent children.
METHOD: Four hundreds and four primary school students without history of trauma or serious medical conditions were investigated. Self-reported questionnaire and physical examination were done.
RESULTS: One-week and 1-year overall pain prevalence were 25.9% and 33.7%, respectively. Prevalence of wide spread pain (WSP) was 7.2% and that of myofascial pain syndrome (MPS) was 7.9%. Prevalence of overall pain and MPS was higher in the 6th grade students than the 4th grade. WSP was more frequent in girls than boys. Joint hypermobility, physical fitness, body mass index and life style including computer use, regular exercise and satisfaction to desk-chair did not affect pain prevalence.
CONCLUSION: Musculoskeletal pain was common in preadolescent children. Age and sex rather than physical state or life style seem to be the contributing factors to pain prevalence.

99: Korean J Prev Med. 2002 Feb;35(1):72-75. Korean.

Investigation of Health Hazards in the Underground Storage Facilities of

Ginger Roots.

OBJECTIVES: To evaluate the health hazards in the underground storage facilities of ginger roots.
METHODS: The authors reviewed the emergency rescue records from the Seosan fire department over the period Jan 1, 1996 to Aug 31, 1999. The atmospheres in 3 different underground storage locations were analyzed for O2, CO2, CO, H2S and NH4.
RESULTS: From the emergency records, we were able to identify 20 individuals that had been exposed to occupational hazards in the underground storage facilities. Among these 20 cases, 13 were due to asphyxiation (resulting in 7 deaths) and 7 were due to falls. In the first atmospheric tests, performed on Feb 25, 1998, the O2 level inside the underground storage facility, located about 5~6 meters below the surface, was 20.6% and the CO2 level was about 1,000 ppm. CO, H2S and NH4 were not detected. In the second tests on Jul 6, 1999, measurements of the O2 level at 3 meters below the surface in two different storage locations were 15.3 and 15.1%. And the O2 levels inside the storage facilities were 12.2 and 12.1%. The CO2 level was above 5,000 ppm (beyond upper limits of measurement). CO, H2S and NH4 were not detected.
CONCLUSIONS: We conclude that asphyxiation in the underground storage facilities for ginger roots was not due to the presence of toxic gases such as CO, H2S and NH4, but rather the exclusion of oxygen by carbon dioxide was responsible for causing casualties. For the development of a hazard free working environment, safety education as well as improvements in storage methods are needed.

100: J Korean Acad Rehabil Med. 2001 Oct;25(5):818-826. Korean.

Prevalence of and Risk Factors for Carpal Tunnel Syndrome in a Rural Population.

OBJECTIVE: The purpose of this study was to investigate the prevalence of and risk factors for carpal tunnel syndrome in a rural population in Korea.

METHOD: Among the 1004 residents in a rural district who participated in the health examination, 450 (165 male, 285 female) adults aged between 30 and 79 years were randomly selected. Hand symptom questionnaire and electrodiagnostic studies were used to diagnose and classify carpal tunnel syndrome. General characteristics, female-related factors, work-related factors and anthropometric measurements were compared between normal and carpal tunnel syndrome group to identify the risk factors for carpal tunnel syndrome.

RESULTS: Subjects with carpal tunnel syndrome were 76 (16.9%), symptom only subjects were 168 (37.3%), asymptomatic slowing 27 (6.0%) and peripheral polyneuropathy were 16 (3.6%). Age, farming, body mass index and wrist depth width ratio were associated with risk of carpal tunnel syndrome and odds ratio were 1.03 (95% confidence interval 1.01~1.07), 2.62 (95% confidence interval 1.17~5.86), 2.24 (95% confidence interval 1.14~4.40) and 3.13 (95% confidence interval 1.64~5.96), each.

CONCLUSION: These data suggest that the prevalence of carpal tunnel

syndrome is high in a rural population and physical factors like wrist shape and body mass index, occupation and aging are associated with risk of carpal tunnel syndrome.

101: Korean J Epidemiol. 2001 Jun;23(1):59-68. Korean.

An Epidemiologic Study on Sudden Deaths of Cattle Occurred in Kyongju.

PURPOSE: This study was conducted to provide the baseline data for the epidemiologic and microbiologic investigation for the etiology of sudden deaths of cattle in Sara-Ri, Seo Myun, Kyongju.

METHODS: This survey was performed between April 11 and 22, 1994. Epidemiologic investigation consisted of interview of the residents, as well as pathologic and microbiologic test on tissues and blood samples from cardiac puncture.

RESULTS: The dead numbers of cattle were 149 in 35 households during about 20 years. The cows(63.9%) were more than bulls(36.1%) and most of them were raised in playpen(95.7%). The first death occurred in 1974, and then number of deaths increased until 1994. Besides the age of cattle at death was over two years old (88.3%), most of them(69.4%) died within one hour after onset of noticeable symptom by the farmers. The most common symptom of cattle at death was 'sudden death after screaming(71.1%)' and 'seizure (33.3%)'.

Colonies from blood of case 3 showed double hemolysis in blood agar plate. The microbiologic test results in the culture of Clostridium perfringens. The pathological features were characterized as most of renal tubules

revealed coagulative necrosis. Some gram-positive bacilli are scattered in interstitium.

CONCLUSIONS: Above results suggest C. perfringens as a possible pathogen of this ourbreak in livestock. The possibility of human infection, although nonfatal, and lack of vaccination against C. perfringens raises a need for stronger preventive action toward this communicable disease of cattle on this village.

102: Korean J Epidemiol. 2001 Jun;23(1):23-35. Korean.

Epidemiological Researches on the Health Hazards in Veterans of United States of America.

The Department of Veterans Affairs(VA) maintains some large automated databases that provide the opportunity for studying long-term health effects of military service. The Beneficiary Identification and Record Locator Subsystem(BIRLS) is an excellent source of vital status information on veterans. The VA Patient Treatment File(PTF) is a computerized hospital discharge abstract system of inpatient records, including patients' demographic data, surgical and procedural transactions, and patient movements and diagnosis. The computerized Agent Orange Registry data include veteran's name, address, some information on military service, and findings at the time of his physical examination.

The US conducted 235 atmospheric nuclear tests from 1945 through 1962. Many of the 250,000 test participants were exposed to low levels of

radiation. The overall average radiation dose was estimated as 0.6 rem per year. In 1976, a claim relating acute myelocytic leukemia to radiation exposure from nuclear weapon testing received extensive publicity. Several thousand "atomic veterans" have sought medical care and compensation from VA for medical conditions that they believe are related to the nuclear weapon testing. Many WWII veterans have contracted the US VA about health problems that they attribute to their exposure to mustard gas.

From 1962 to 1971, 75 million liters of herbicides, including over 41 million liters of the phenoxy herbicide Agent Orange, were sprayed on almost 9% of Vietnam. Many studies have been conducted to determine the association of various cancers with military service in Vietnam. Some diseases have been compensated for Vietnam veterans. Health problems reported following the Gulf War include a wide variety of symptoms similar to those found in acute combat reaction, posttraumatic stress disorder, and chronic fatigue.

Health problems associated with war have continued and in some ways intensified. Therefore, The United States developed a plan for establishing a national center for the study of war-related illnesses and post-deployment health issues.

103: Korean J Leg Med. 2001 May;25(1):40-43. Korean.

Asphyxia due to Oxygen Deficiency in the Cargo-hold Shipping Wood.

Oxygen deficiency has been frequent in a closed space. Wood consumes oxygen

and discharges carbon dioxide instead of photosynthesis in closed space without light, so do some microorganisms on the surface. We experienced a case that a healthy insect-proofer fell down and died of asphyxia on stair-board at 7 m below the hatch of the cargo-hold shipping wood. Analysis of gases in cargo-hold revealed O2; 12.3%, CO; 105 ppm, CH4; 2.7%, and H2S; 1.9% at 1 m below the hatch, and then O2; 6.1%, CO; 220 ppm, CH4; 2.9%, and H2S; 2.3 ppm at 2.5 m below the hatch. Autopsy findings were unremarkable. We justiced the cause of death asphyxia due to oxygen deficiency. As seen in this case, the serious oxygen deficiency was accounted for oxygen consumption by wood and microorganisms.

104: Korean J Prev Med. 2001 Feb;34(1):80-88. Korean.

A Study on the Correlation between Categorization of the Individual Exposure Levels to Agent Orange and Serum Dioxin Levels Among the Korean Vietnam Veterans.

OBJECTIVES: In an epidemiologic study on the health impact of Agent Orange exposure, the valid estimation of exposure level is the most important step. Based on recent studies, we examined the correlation between exposure levels categorized by personal exposure estimates and serum 2,3,7,8-tetrachlorodibenzo-p-dioxin (2,3,7,8-TCDD, Dioxin), exploring the possibility of utilizing the exposure level as a surrogate for the estimate of exposure to agent orange.
METHODS: During the study period (Jan 1996-Feb 1996), blood specimens of

745 subjects taken randomly among 1,329 persons and kept frozen, were analyzed for 2,3,7,8-TCDD and six other dioxin congeners. The serum dioxin and congeners were measured in 1998 by CDC ,adjusted for serum lipids. We categorized the total exposure scores into five groups based on Agent Orange exposure data collected by interview and military records. Pearson and Spearman's correlation coefficients & multiple regression analysis were used to identify the relationship of the exposure level categorized with serum concentration of 2,3,7,8-TCDD, and six other dioxin congeners.

RESULTS: Dioxin and the other congeners, except 1,2,4,6,7,8-HpCDD, showed significant correlations to exposure categories ($p<0.005$); 2,3,7,8-TCDD and OCDD showed positive correlations, whereas the other congeners did negative. The values of 2,3,7,8-TCDD differed according to exposure category and proportionally increased from the low exposure group to the high, a dose-response relationship, even after other possible confounding variables were adjusted for. In multiple regression analysis, age(beta=0.033),

dioxin(beta=0.433), 1,2,3,7,8-PeCDD(beta=0.998),

1,2,3,4,7,8-HxCDD(beta=0.773),

1,2,3,6,7,8-HxCDD(beta=0.255), 1,2,3,7,8,9-HxCDD(beta=3.468),

1,2,3,4,6,7,8-HpCDD(beta=0.109)
were found to be significantly related to the total exposure
score($p<0.005$).
CONCLUSION: This study demonstrated that the use of such categorizations as
a
surrogate measure of agent orange exposure in identifying exposure degrees
in a health
impact study is valid.

105: Korean J Epidemiol. 2000 Dec;22(2):148-158. Korean.

Epidemiologic Investigation of a Mumps Outbreak in a Middle School in Pohang, Korea: Effect of vaccination during outbreak.

BACKGROUND: There was a widespread outbreak of mumps at elementary and middle schools in Pohang city in March 1999. We have carried out an epidemiologic survey to trace the source and mode of transmission of the outbreak, as well as evaluating the effect of vaccination as a measure of controlling the outbreak.
METHODS: Questionnaire survey on 959 students showed that attack rate was 10.7%. There was no significant difference between genders in terms of attack rates. However, second graders outnumbered other groups as 16.9%,

comparing to 8.1% among first graders and to 7.0% in the third graders.

RESULTS: Cases of mumps were reported in 1998, primarily in the neighboring elementary schools. The disease was also reported earlier in 1999, sporadically. In March, however, number of the cases was sharply on the rise as the middle schools opened for a new semester. Our investigation revealed that infection stemmed from more than three different sources, particularly in the classes of second graders, and then spread to all students, corresponding to the distance between students. Effect of vaccination during the outbreak was pretty good: attack rate of the group vaccinated since May 1st, considering the maximum latent period of infections (21 days), was 0.8%, comparing to 5%. The efficiency of vaccination marked 84.8%.(95% confidence interval 79.66-89.94) Although the authors recommended the students to wear flue masks and wash their hands as often as possible to screen infections among the students, it turned out that such measures had little effect for the prevention of spread.

CONCLUSIONS: The number of mumps cases dropped remarkably among vaccinated the students the prevalent period of the disease. Authors concluded that is effective to control the outbreak if it is done at early stage.

106: Korean J Prev Med. 2000 Dec;33(4):477-483. Korean.

Asbestos and Non-Asbestos Fiber Content in Lungs of Autopsied Subjects in Pohang with no Known History of Occupational Asbestos Exposure.

OBJECTIVES: To obtain reference values for the pulmonary asbestos and

non-asbestos fiber contents of residents in Korea and to compare them with similar results from Japan.

METHODS: The autopsied lung specimens from 22 deceased people (20 males and 2 females) in Pohang, without any known occupational history of asbestos exposure, were analyzed for incidence of asbestos and non-asbestos fibers by transmission electron microscopy with energy dispersive X-ray analysis after using low temperature ashing procedures.

RESULTS: Chrysotile fiber (46.2%) was the major fiber type found in the lungs of the subjects. The asbestos fiber concentrations found in males and females were 0.09x106 fibers/(g of dry lungs) and 0.30x106 fibers/(g of dry lungs), respectively, showing a geometric mean concentration 0.09x106 fibers/(g of dry lung tissue), due to the predominance of males in the sample. The non-asbestos fiber contents in males and females were 4.61x106 fibers/(g of dry lungs) and 17.79x106 fibers/(g of dry lungs), respectively, with a geometric mean concentration 5.21x106 fibers/(g of dry

lung tissue).

CONCLUSIONS: Residents in Pohang had significantly lower levels of both asbestos and
non-asbestos fibers than urban residents in Korea. Furthermore, Koreans had

significantly lower levels of both asbestos and non-asbestos fibers than Japanese.

107: Korean J Occup Environ Med. 2000 Jun;12(2):218-226. Korean.

Study on the Pre-employment Lumbo-sacral Simple X-Ray Examination.

OBJECTIVES: A study was conducted for investigating the status of simple X-rays on lumbosacral
regions at pre-employment health examination and analysing the effectiveness of simple X-rays on lumbosacral regions.

METHODS: The study data were pre-employment health examination data in a university hospital from Jan 3, 1993
through October of 1997. And, 97 newly employed workers who claimed no low back pain at pre-employment health
examination of a certain company were follow-up survey about low back pain and related factors in April, 1995
and October of 1997. The data were collected by a self-reported questionnaire and a medical examination with

a rehabilitation specialist.

RESULTS: Taking the simple X-rays on lumbo-sacral regions was 1, 591 workers(10.5%) among 15,166 pre-employment health examination from Jan 3, 1993 through October of 1997. And yearly application rates of these X-ray tests have been significantly statistical increasing ($p<0.01$). 2. The abnormal findings of simple X-rays on lumbo-sacral regions at pre-employment health examination were 40 workers. But, there was not significantly statistical difference between normal and abnormal group for the complaint rate of low back pain. 3. The number of case with symptoms of low back pain at the follow-up survey were 46, so the incidence density was 37. 1 persons/ 100 person-years. And the incidence density was not significantly statistical difference between normal and abnormal group of simple X-rays on lumbo-sacral regions. 4. Through the multivariate logistic regression, significantly associated factors with low back pain were found to be tenure(OR=0.36, 95%CI: 0.17-0.79), lifting of heavy materials(OR=5. 86, 95%CI: l. 58-21. 74).

CONCLUSION: The simple X-rays on lumbo-sacral regions at pre-employment health examination is required further research for utilizing pre-employment health examination according to above results.

108: Korean J Prev Med. 2000 Jun;33(2):184-192. Korean.

Prevalence and Risk Factors of Myofascial Pain Syndrome on School Boys.

OBJECTIVES: To inquire the prevalence and the risk factors for myofascial pain syndrome (MPS) on young boys in order to use these results as the fundamental data for the prevention of their MPS.

METHODS: For 7 days in May 1999, this research was taken on 489 male students ranging from 6th to 12th grade. We randomly selected a class for every group and from these classes we operated physical examinations, self-reported questionnaires and from a rehabilitation doctor, MPS test was taken. Thoracic kyphosis and lumbar lordosis were also taken by using the inclinometer. We defined MPS as a regional pain complaint, palpable taut band that is painful on compression.

RESULTS: The shoulder MPS prevalence of the subjects were 29.7 persons/100 persons. The statistics revealed that as grades went up, the percentage significantly increased in the MPS prevalence. As of case-control study, 145 students who were tested postive in all aspects were placed as cases, and 176 students who were perfectly

normal as
controls on risk factors. As a result of comparing the student groups who
were satisfied
with their chairs to the student groups were not satisfied, the latter
showed a
significantly higher odds ratio ($p<0.01$). By the multiple logistic
regression test, we
concluded that the MPS disease was prevailed far more in the students in
the higher
grades (Odds ratio: 1.16, 95% C.I.: 1.03-1.31), and also those who were
dissatisfied with
their chairs than in the ones who were satisfied (Odds ratio: 1.92, 95%
C.I.: 1.17-3.17).
CONCLUSIONS: Significant correlations showed between the MPS diagnosed
group and the
students who are dissatisfied with their chairs. As a result, more research
and
observation has to be made concerning this disease, and the desks and
chairs should be
adjusted to suit the student? physical standards.

109: Korean J Prev Med. 2000 Mar;33(1):10-16. Korean.

Epidemiologic Investigation of an Outbreak of Shigella sonnei among
Students in Bonghwa, 1999.

OBJECTIVES: This study was carried out to investigate the sources of infection and modes of transmission of an outbreak of shigellosis that occurred among students of B middle and high school in Bonghwa, Korea from May 1 to 21, 1999.

METHODS: We conducted questionnaires to 468 students, 38 staffs and 9 food handlers twice times (May 6, May 21) for follow up and secondary attack rate. Personal details and history of illness and exposure to particular foods were sought. And we conducted rectal swab for culture to 243 students, 33 staffs and 9 food handlers. Bacteriological examinations of water in the school were done. Cases were identified as subjects who had diarrhea (two or more loose stools in a 24-hour periods) on or after May 1.

RESULTS: A total of 307 cases (attack rate: 59.6%) of 515 subjects were identified, including 50 confirmed (46 students and 4 staffs) by S. sonnei. All 9 food handlers denied illness and were had rectal swab for culture at May 6 that were negative for S. sonnei. 146 of 307 reported fever, 156 had tenesmus, 44 reported vomiting, and only 5 of 307 reported blood in the stool. The median duration of diarrhea was 4 days

(range:

1-18 days). The mean incubation period until onset of diarrhea was 63 hours

(range:

16-144 hours) and the secondary attack rate was 2.8% (43 cases of 1,561

family

members). Risk for illness was higher among students who had eaten watered

kimchi at

March 30 than among those who did not [301(72.7%) of 417 versus 5(9.6%) of

52;

RR=7.51; 95% CI=3.26-17.31].

CONCLUSION: The source of infection was estimated to be contaminated

watered kimchi

by one or two food handler who is presumed to be carrier.

110: Korean J Prev Med. 2000 Mar;33(1):1-9. Korean.

Epidemiologic Investigation of an Outbreak of Shigellosis in Kyongju, Korea.

OBJECTIVES: This study was carried out to investigate the sources of

infection and modes of transmission of an outbreak of shigellosis that

occurred among pupils of "M" primary school and residents near the school

in Kyongju from Sept. 24 to Oct. 24, 1998.

METHODS: The subjects who completed a questionnaire and a rectal swab for

microbiologic examinations were 1,534 persons (781 males, 753 females),

including 469

pupils of "M" primary school (268 males, 201 females). Bacteriological examinations of
underground water and simple piped water were done.
RESULTS: The attack rate of diarrhea was 28.7% in the subjects from Sept. 24 to Oct.
24, 1998. There was no difference in attack rate of diarrhea by gender, but it was
significantly higher in the pupils of "M" primary school than others ($p<0.01$). The
attack rate of diarrhea by resident areas was no different to the pupils of "M"
primary school, but was significantly higher in the residents of Mohwa 2 Ri except
pupils that "M" primary school is located in ($p<0.01$). The distribution of date of
onset revealed the exposure date to be Sept, 22 and 23 in consideration of incubation
periods and common source outbreak followed propagative spread in the epidemic curve.
The major characteristics of diarrhea were watery (89.1%) in nature, 1~3 days (72.5%)
in duration, 2~3 times (63.9%) in frequency. The clinical symptoms among the diarrheal
cases included abdominal pain (74.1%), fever (56.4%), headache (55.9%), chill (40.4%)
and tenesmus (31.4%).

CONCLUSIONS: The source of infection was estimated to be contaminated underground
water and simple piped water caused by leakage from the cess pool. It is highly
necessary that the management of drinking water and cess pools should be done
thoroughly.

111: J Korean Acad Rehabil Med. 2000 Feb;24(1):100-107. Korean.

The Significance of Anti-type I Collagen Antibody Titer in Occupational Low Back Pain.

OBJECTIVE: To assess the significance of anti-type I collagen antibody titer in estimating cumulative trauma and predicting the presence of occupational low back pain.
METHOD: Under the hypothesis that cumulative trauma on the spine will expose collagen and stimulate the formation of auto-antibody, we measured the serum anti-type I collagen antibody titers (IgM and IgG) in 408 male workers of a metal welding and manufacturing company. The antibody titers were measured in duplicates by ELISA. Statistical analysis was done to compare the titers according to occupational profiles (type of occupation and duration of employment) and clinical profiles (occurrence of low back pain, duration of low back pain and clinical impression).
RESULTS: The anti-type I collagen IgG antibody titers were significantly

increased in labor workers (n=357) in comparison with office workers (n=51)(p<0.05). Among the labor workers both IgM and IgG antibody titers were increased in the low back pain group (n=50) though it did not reach statistical significance (p-value of IgM antibody titers=0.07). IgM and IgG antibody titers were increased in the chronic low back pain group (> or =3 months)(n=8).
CONCLUSION: These data suggest that anti-type I collagen IgM and IgG antibody may be useful in predicting the presence of occupational low back pain and estimating cumulative trauma, respectively.

112: Korean J Epidemiol. 1999 Dec;21(2):159-175. Korean.

Development of Lipoma among Residents Exposed to Glass Fiber Waste.

This study was conducted to determine the relationship between exposure to glass fiber waste from an insulator factory and the development of cluster of lipomas among local residents in suburb Incheon, Korea. Authors surveyed 152 residents(71 males and 81 females) living near an insulator factory with a questionnaire and physical examination. Unused and disposed fiberglass from the waste site, along with ground water samples were examined under light and polarizing microscope and scanning electron microscope(SEM). Subcutaneous tumors excised from three of the residents were also examined under light and polarizing microscope, SEM with energy dispersive X-ray analysis(EDX), and transmission electron microscope(TEM). Analysis of elemental composition of the fibers and fiber concentration was

done by with EDX under SEM and TEM after low temperature ashing.

Twelve(7.9%) had subcutaneous tumors among the 152 residents. Tumors were surgically excised from 3 of them and they were all lipomas, consisting of mature fat tissue. These lipomas contained abundant birefringent fibers and particles under polarizing microscope. The concentrations of the fibers were 6.7, 71.8 and 499.2 million fibers per gram dry tissue, respectively. The birefringent fibers were composed of needle shaped particles with rectangular fractured ends up to 17 micrometer in length and 0.5 micrometer in diameter. EDX and x-ray diffraction analysis of the fibers showed that 71 to 100% of the fibers were magnesium silicate, talc. Magnesium silicate fibers were also found in the glass fiber sampled from the waste site. Glass fibers and magnesium silicate fibers were also identified in the ground water.

Based on the fact that the magnesium silicate fibers found in the lipomas were similar in morphology and elemental composition to those found in the ground water and those from the waste site, these particles are likely to be introduced into the gastrointestinal tract through consumption of the contaminated ground water. It is suggested that fibrous magnesium silicate, talc, a component of fiberglass waste, may be associated with the development of lipomas.

113: Korean J Occup Environ Med. 1999 Dec;11(4):546-556. Korean.

Effects of occupation, life style and genetic polymorphism of CYP1A1, GSTM1, and GSTT1 on urinary 1-hydroxypyrene and 2-naphthol concentration.

OBJECTIVES: This study was performed to describe the distribution patterns of urinary 1-hydroxypyrene (1-OHP) and 2naphthol concentration in coke oven workers and workers not occupationally exposed to polycyclic aromatic hydrocarbons (PAH), and to determine the effects of occupation life style, and genetic polymorphism of cytochrome P450 1A1 (CYP1A1), glutathione Stransferase mu 1 (GSTM1) and theta 1 (GSTT1) on urinary 1- OHP and 2-naphthol concentration.

METHODS: The study subjects were 19 coke oven workers and 156 shipyard workers. A questionnaire was used to obtain data about detailed smoking and food intake history. Urinary 1OHP and 2naphthol concentration and genetic polymorphism of CYPIA1, GSTM1, and GSTT1 were analyzed.

RESULTS: The urinary 1-OHP and 2-naphthol concentration was higher in the coke oven workers and in smokers. Urinary 1-OHP concentration was significantly correlated with time after last intake of roasted meat in non-smoking coke oven workers, whereas urinary 2-naphthol concentration was with amount of cigarette smoking at the sampling day in smoking shipyard workers. Urinary 1-OHP, but not 2-naphthol, concentration of the shipyard workers with Ile/Ile type of CYP1A1 vas significantly lower than that of the shipyard workers with other CYP1A1 genotype.

CONCLUSIONS: Urinary 1-OHP would be a better marker for occupational exposure to PAH in coke oven workers, and urinary 2naphthol might be better for non-occupational inhalation exposure to PAH. CYP1A1 would not play an important role in the metabolism of naphthalene but in the metabolism of pyrene.

114: Korean J Pathol. 1999 Nov;33(11):1024-1032. Korean.

Talc Deposition in Lipoma: A Pathologic and Mineralogic Study.

Three cases of lipomas associated with heavy talc deposits are reported in local inhabitants near a fiber glass factory. Pathologic and mineralogical analysis by polarizing microscopy, scanning and transmission electron microscopy with energy dispersive X-ray analysis and X-ray diffraction of mass were done. Simultaneously, we performed an epidemiological survey and a mineralogical study of disposed fiber glass from waste site and ground water. All tumors consisted of mature fat tissue containing an abundant birefringent talc fibers and particles under polarized light. The concentrations of the fibers were 494.7, 6.7 and 50.7 million fibers per gram of dry tissue. The fibers were needlelike with rectangular fractured ends, up to 17 micrometer in length and 0.5 micrometer in diameter. EDX and X-ray diffraction analysis of the fibers showed that 71 to 100% of the fibers were magnesium silicate, talc. We also identified magnesium silicate fibers in the fiber glass from the waste site and in the

ground water which
were similar to talc fibers in lipomas,. We concluded that fibrous talc, a component of
fiberglass waste, might be associated with the development of lipomas via unknown exposure
route.

115: Korean J Pathol. 1999 Sep;33(9):662-674. Korean.

Manganese Intoxication in the Rat   A neuropathologic study and distribution of manganese in rat brain.

We investigated a topographical distribution of managanese, and immunohistochemical density of tyrosine hydroxylase (TH), and histopathologic findings
in globus pallidus and substantia nigra according to manganese dose and time course in
the brain of rats which received MnCl2 intravenously. Topographical distribution of
manganese was also investigated after injection of FeCl2. The manganese concentrations
of brain in control and experimental group were highest in pituitary gland and thalamus,
and lowest in the cerebral cortex. The manganese concentration of blood was increased

proportionally to the dose administered, and the biological half-life of blood manganese was between 21 and 42 days. The manganese concentrations of brain were increased proportionally to the dose, and increase rate was highest in olfactory bulb, and the biological half-lives of brain manganese ranged from 42 days to 90 or more days; the longest were observed in pituitary gland, medulla oblongata and cerebral cortex. In case of administration of FeCl2, the manganese concentrations of brain were higher than that of control group in dose of 2.5 mg/kg, and decreased proportionally to the administered dose, resulting in lower level compared with control group in high dose of FeCl2 administered. Significantly decreased number of nerve cell and increased gliosis in globus pallidus were observed in experimental group, which were closely correlated with the duration after manganese injection, but no significant change of number of nerve cell expressing TH and gliosis were observed in substantia nigra. Density of immunohistochemical reaction for TH in globus pallidus made little difference between control and experimental group. These results suggest that pathology of

manganese
intoxication is caused by the loss of nerve cells in globus pallidus, and
closely correlated
with the duration after manganese exposure.

116: Korean J Prev Med. 1999 Sep;32(3):421-426. Korean.

Epidemiologic Survey on Outbreak of Dermatosis Associated with Ants,
Pachycondyla chinensis.

OBJECTIVES: An outbreak of dermatosis occurred at a city gas manufacturing
factory in
Pohang in Aug, 1998. The authors conducted a study to find the cause and
prevent the
dermatosis.
METHODS: We conducted a questionnaire survey of 73 workers in the factory
twice,
once on Aug. 14th and then Sept. 11th, 1998. Also, a dermatologist examined
their skin
lesions. We suspected ants as the cause, so we collected them for
identification.
RESULTS: Twelve cases of dermatosis were identified with an attack rate of
16.4%. The
attack rates were not different by age, educational level or tenures. The
attack rates

were 66.6% among production workers, 2.0% among clerical workers, 16.7% among tank trailer drivers and 0% among guards. The attack rate among production workers was significantly higher than that of the others ($p<0.01$). There were no histories of the same dermatosis for the past one year. Histories of other skin diseases and allergies were very rare in both cases with skin diseases and control. Multiple, pruritic, rice-grained to bean sized erythematous macules or papules with a central biting point could be found after initial severe itching occurred. Three cases showed signs of dermatosis with an allergic nature. The onset of dermatosis was between July 30 and Aug 12 and the durations varied from 5 days to over a month. The most frequent sites of skin lesions were the chest and abdomen (66.6%), and they were also observed on the neck (33.3%), arms (33.3%), shoulders (16.7%) and back (16.7%). Over 10 ants with their wings were collected in the work place and identified as Pachycondyla chinensis, subfamily Ponerinae.

CONCLUSIONS: We thought that the outbreak of dermatosis was brought about

by the

ants flying into the work place through the open windows. Further studies

on the

factors contributing to the prosperity of the ants in this area are needed.

117: Korean J Epidemiol. 1999 Jun;21(1):64-71. Korean.

Occupational Relationship of Cancer Patients Diagnosed in Two University Hospitals.

OBJECTIVES: This study was performed to evaluate the occupational relationship on 190 cases of cancer selected out of 622 cases of cancer registered in two university hospitals from January 1, 1996 to December 31, 1997.

METHODS: The selection criteria was for the patient to be more than 40 years old with lung, liver, urinary bladder, nasal cavity and skin cancer or leukemia. We reviewed the medical records to update the missing data and occupational histories. Telephone interviews were used to obtain complete occupational histories on the subjects.

RESULTS: The sites of cancer in the order of relative frequency was lung (51.0%), followed by liver (32.9%), urinary bladder (14.1%) and skin (2.0%) in male, liver (41.5%), followed by lung (31.7%), skin (19.5%) and urinary bladder (7.3%) in female. The occupational histories of 190 cases with suspected cancer-causing occupations were recorded 5.8% on the doctor's medical records and 33.2% on the nursing records. The response rates of the

telephone interviews were 87.4%. The distribution of occupation according to the telephone interviews was farmer (47.7%), office worker (16.1%), salesman (12.8%), production worker (6.7%), simple laborer (3.4%) and unknown (13.4%) in male, housewife (63.4%), farmer (17.1%), saleswoman (9.8%) and unknown (9.8%) in female. And there were two cases of suspected occupational relationships in the lung cancer cases.

CONCLUSIONS: We could not discover definite cases of occupational cancer but found out two cases of suspected occupational relationships. Occupational cancer is likely to increase in the near future, so the efforts to detect occupational relationships with cancer should be continued.

118: Korean J Epidemiol. 1999 Jun;21(1):36-52. Korean.

Incidence Density and Risk Factors of Low Back Pain among the Workers in a Welding Material Manufacturing Factory.

This study was performed to understand the incidence density and detect the risk factors of occupational low back pain. A cross-sectional study was conducted with a questionnaire in a welding material manufacturing factory in October, 1993. Therefore, we selected 140 workers who had never complained of low back pain as cohorts. Also, we added 236 newly entered persons who had never complained of low back pain. The total number of cohorts were 376 workers. And then we continued with a questionnaire survey in October, 1996 and with a questionnaire survey and medical examinations

by a specialist in October, 1997. Follow-ups were done for 337 workers.

The number of newly developed low back pain among workers were 127. The characteristics of low back pain were as follows. The durations of pain were less than or equal to 2 days (42.6%), from 3 days to less than 1 week (8.7%), from 1 week to less than 1 month (11.0%), 1 month or more (6.2%). The frequency was everyday (7.9%), once per week (21.3%), once per month (14.2%), once per 2-3 months (9.4%), once per 5 months (11.0%). The severity of pain was slight (9.4%), mild (33.1%), moderate (13.4%), severe (10.2%) and very severe (1.6%). The onset of most low back pain was insidious (41.7%). The diagnosis of low back pain was muscle strain (37.8%), lumbar sprain (23.6%) and myofacial pain syndrome (3.9%).

The number of newly developed low back pain among workers were 127, their incidence density was 15.7 per 100 person-years. In univariate analysis, age, marital status, educational level, smoking habit, category of job, tenures and frequency of stretching exercises showed a statistical significance. The multivariate logistic regression analysis confirmed that category of job and tenures ($p<0.05$) were independent risk factors for low back pain among workers.

The number of newly developed occupational low back pain among production workers were 71, their incidence density was 11.3 per 100 person-years. In univariate analysis, age, marital status, educational level, regular exercise, tenures, posture of waist and lifting of heavy materials showed a statistical significance. The multivariate logistic regression analysis confirmed that posture of waist ($p<0.05$) and lifting of heavy materials ($p<0.1$) were independent risk factors for occupational low back pain among production workers.

119: Korean J Occup Environ Med. 1999 Jun;11(2):181-195. Korean.

Health Hazards and Diagnostic Methods of Glass Fiber Workers.

This study was conducted to evaluate the health hazards and to develop diagnostic methods of glass fiber workers. We examined 40 male glass fiber workers (exposure group) and 57 male non-glass fiber workers (reference group) with a questionnaire, physical examination, chest x-ray and pathological examination in Mar, 1997. Also we examined 65 male glass fiber workers (exposure group) and 42 male non-glass fiber workers (reference group) with the same methods also we did some energy-dispersive x-ray analyses with a scanning electron microscopic examination in Sep. 1997.
Most of the clinical symptoms were significantly more frequent among the exposure group than the reference group. Coughing (32.5%), itching of the nose (30.0%), irritation of eyes (27.5%), irritation of the nose (25.0%) and sputum (22.5%) were the major symptoms among the exposure group in Mar. 1997. Only itching of the nose was significantly more frequent in Sep.
1997. No cases of pneumoconiosis were observed among the groups and there were no differences in chest abnormalities between the exposure group with the reference group on both examinations. The prevalence of dermatosis among the exposure group was 20.0% (8 cases) and the cumulative prevalence was 72.5% (29 cases) in Mar. 1997. The prevalence of dermatosis among the exposure group was 23.1% (15 cases) and the cumulative prevalence was 58.5% (38 cases) in Sep. 1997. Onset of dermatosis among the exposure group was

most frequent within one month after handling. The frequent sites of skin lesions were the hands and arms on both examinations. Glass fiber induced skin lesions can be diagnosed by the scotch tape method or KOH mount and then can be examined under the light, polarizing and phase-contrast microcopies. Glass fibers can be identified by some energy-dispersive x-ray analyses with a scanning electron microscopic examination.

120: Korean J Anesthesiol. 1999 Mar;36(3):524-528. Korean.

Hypercarbia Due to Mistaken Supply of Carbon Dioxide Originating from Nitrous Oxide Gas Tank: A case report.

A 49 year-old male was scheduled for a cholecystectomy, thereafter a 37 year-old female scheduled for removal of a epidural hematoma in the same operating room. Both of them had no specific medical problems and past medical histories for anesthesia. For those reasons, anesthesia was induced with thiopental sodium and succinylcholine with endotracheal intubation. After induction, vital signs including body temperatures were stable. But moisture dew in the unidirectional valves and corrugated tubes, and color changes of soda lime were discovered. At that time, severe hypercarbia was recognized by arterial blood gas analysis in both cases. In both cases, there were no malfunctions in unidirectional valves, expiratory valves, corrugation tubes, soda lime, ventilators and there connection parts in the anesthetic machines. Also there were no abnormalities of blood pressures, electrocardiograms, pulse oxymeters, temperatures and the pulse in the

patient monitoring systems except capnography. At first, we thought that medical signs revealed malignant hyperthermias. But vital signs, air way pressures and functions of all kinds of anesthetic machine components including ventilators were normal. After discontinuing N_2O gas deliveries in the operation room, hypercarbias disappeared. Thus, anesthetic gas delivery systems via central piping systems were checked and it was discovered that CO_2 gas was in the N_2O gas tank instead of N_2O.

121: Korean J Occup Environ Med. 1999 Mar;11(1):52-65. Korean.

Prevalence and Risk Factors of Occupational Low Back Pain among the Production Workers in a Steel and a Welding Material Manufacturing Factories.

A cross-sectional study was conducted for detecting the risk factors and to propose an effective control program for occupational low back pain. The subjects were 1,665 male production workers employed at a steel factory and a welding material manufacturing factory. The data were collected by a self-reported questionnaire and a medical examination with a rehabilitation specialist for ten days in September, 1997. The contents of the questionnaire were as follows: general characteristics, physical characteristics, employment status, type of work, working environment and the experience of low back pain. The number of cases with symptoms of occupational low back pain were 321, so the point prevalence was 19.3 persons/100 persons. The number of cases with a history of occupational low

back pain for one year were 554, so the one year period prevalence was 33.3 persons/100 persons. The point and one year period prevalences of occupational low back pain showed no significant differences in age, marital status, educational level and body mass index. The point and one year period prevalences of occupational low back pain showed no significant differences in alcohol drinking, smoking, stretching exercise and regular exercise. The point and one year period prevalences of occupational low back pain were significantly higher in the unsatisfied group than the satisfied group($p < 0.01$), However, no significant differences were found among tenures and shift work. The point and one year period prevalences of occupational low back pain were significantly higher in the lifting of heavy materials group than the nonlifting group($p < 0.01$). However, no significant differences were found among posture of the waist and the working posture. Through the multivariate logistic regression, significantly associated factors with occupational low back pain were found to be dissatisfaction with job(point prevalence: OR=1.78, 95% CI: 1.21-2.61; one year prevalence: OR=1.76, 95% CI: 1.26-2.47), lifting of heavy materials(point prevalence: OR=1.94. 95% CI: 1.44-2.61: one year prevalence: OR=2.17, 95% CI: 1.70-2.77) and tenure(point prevalence: OR=1.03. 95% CI: 1.01-1.06: one year prevalence: OR=1.02, 95% CI: 1.00-1.05).

122: Korean J Epidemiol. 1998 Dec;20(2):202-211. Korean.

An Epidemiological Investigation of an Outbreak of Rubella Occurred in a

Male High School in Kyongju.

This epidemiological study was carried out to investigate the sources of infection and modes of transmission of an outbreak of rubella that occurred among male high school students in Kyongju in march, 1996. 770 male students(286 third grade, 262 second grade, 222 first grade students) were selected as the study subjects. A questionnaire was completed by the subjects. The anti-Rubella antibodies IgM and IgG were examined and tested by ELISA on all sera from 770 students and the environmental status of class rooms, the dormitory conditions and study rooms were tested. The positive IgM result rate was 17.8% and when the IgM negative was combined with IgG positive the results was 74.9%. IgM IgG negative rates were 7.3%, however, out of a total 770 students. Amongst the older students, the IgM positive rate showed an increase($p<0.01$). Out of 137 cases(with a cummulative incidence rate of 71.0%), there were 97 apparant cases(cummulative incidence rate 50.3%) and 40 inapparent cases(cummulative incidence rate 20.7%). Again, the cummulative incidence rate showed an increase in the older students($p<0.04$). In the apparent cases, major symptoms included eruption(96.9%), fever(85.6%), lymphadenitis(82.5%), generalized aches and muscle tenderness(76.3%), and a sore throat(61.9%). These symptoms lasted anywhere from 2 degrees =/ 13 days, the average duration being 4 days in length. Initially, there were 9 people on third grade and suspected origin was from more than 2 external sources. It progressed from the upper grades down to the lower grades. In dormitory students, the cummulative incidence ratio was 1.70 and 1.78 amongst study room students.

123: Korean J Epidemiol. 1998 Dec;20(2):187-201. Korean.

An Epidemiological Survey on a Salmonella enteritidis Outbreak in Kyongju, Korea.

This study was conducted to investigate the cause and magnitude of food poisoning among residents and visitors for a birthday party in a rural area of Kyongju city in september, 1996. The total subjects were 137 persons, 119 residents(53 males, 66 females), including 7 hospitalixed patients and 18 visitors(8 males, 10 females). The investigation consisted of an interview survey, a study on clinical characteristics for the patients hospitalized, bacteriological examinations of the underground water and microbiologic examinations on microbes isolated from the patients. The population at risk was 59 persons(24 males, 35 females). The attack rate was 50.0%(12 cases) for males, 48.6% for females(17 cases). The cases were distributed evenly for all age groups. The attack rates for each party-food and possible fooe groups were not significantly different. Most clinical symptoms were significantly more frequent among cases than non-ases: abdominal pain(73.1%), diarrhea(73.1%), chilliness(69.2%), fever(65.4%), anorexia(61.5%), nausea(53.8%), general weakness(50.0%) and dizziness(50.0%) were the major symptoms among the cases. The admission rate among the cases was 24.1%(1 male, 6 females). In the stool cultures, Salmonella species group D was isolated from 6 persons of 85 examinees. It was identified as Salmonella enteritidis by serological diagnosis. The

samples of underground water were assessed Enon-compatible for drinking in 10 wells(83.3%) of this rural area and it was suggested that it could have been contaminated from cattle feces. There were two wells in the party house, one of them was contaminated by bacterias including E. coli. The contaminated water was stored in a water tank located on the rooftop. This water was used for dish washing and cooking. Wd concluded that the cause of this epidemic was the underground water contaminated by Salmonella enteritidis from guman carriers or domestic animal carriers.

124: Korean J Occup Environ Med. 1998 Dec;10(4):548-561. Korean.

The Development of Health Risk Appraisal at the Worksite.

Health Risk Appraisal(HRA) is usually defined as a process by which we expect of individual's chances of death or acquiring specific diseases within a defined period of time. The concept of worksite as an area of health maintenance and promotion is newly developing. Our movement for health promotion of employees has been increasingly known in the worksite. The aim of this study is to develop health risk appraisal tools about health promotion at the worksite for employees. We performed this study by two steps: one step was to develop a HRA questionnaire of worksites in Korea, another was to evaluate the reliability of the questionnaire. For developing HRA questionnaire, we reviewed scientific examples at first, and then weighted the score by delphi. To evaluate the reliability of developed questionnaire, we carried out survey by test-retest method. A total of 131

employees completed HRA questionnaire on two times. The results are as follows. The total score of health risk in unhealthy workers was higher than that of healthy workers. The range of test-retest reliability of responses to the questionnaire was 0.57-0.94. Therefore it seems that this questionnaire was very suitable to assess the health behavior of workers. In conclusion, the developed HRA questionnaire can be used as a tool for evaluating health behavior and for providing health counseling materials.

125: Korean J Occup Environ Med. 1998 Dec;10(4):493-504. Korean.

Prevalence of Allergic Diseases of Workers Exposed to Phthalic Anhydride.

Phthalic anhydride (PA) is widely used in the production of alkyd and unsaturated polyester resins. It has been reported that some workers exposed to PA have developed dermatitis, rhinitis and asthma. The authors intended to investigate the prevalence of PA induced allergic diseases and to develop preventive measures of occupational diseases. Forty-five male workers from PA production and handling were selected as an exposure group. Forty-four male workers not handling PA at the same factory were chosen as a reference group. A symptom questionnaire, doctor's examination, eosinophil count, serum-total IgE and PA-specific IgE were done on the subjects. There were no significant differences in age, educational level, tenure or smoking habits between groups. Most clinical symptoms were significantly more frequent among the exposure group than the reference group: sneezing(62.2 %), rhinorrhea(57.8 %), nasal stuffiness(53.3 %),

coughing(44.4 %) and nasal itching(35.6 %) were the major symptoms among the exposure group ($p<0.01$, $p<0.05$). There were no significant differences in eosinophil count and serum-total IgE between groups but the mean level in PA-specific IgE, in the exposure group, was statistically significantly higher than the reference group ($p<0.01$). Abnormal rates of eosinophil count, serum-total IgE and PA-specific IgE were not significantly different from each other. The prevalence rate was 68.9 % (31 cases) for allergic rhinitis in the exposure group, statistically significantly higher than the reference group ($p<0.01$). The prevalence rate was 6.7 %(3 cases) for bronchial asthma and contact dermatitis in the exposure group, not more statistically significant than the reference group. In the exposure group, the prevalence rate for age, educational level, tenure, smoking habits and use of protective devices are not statistically significant. In PA-specific IgE, sensitivity is 10.8 %, specificity is 100.0 %, positive predictability is 100.0 % and negative predictability is 61.2 % when criteria point is 3.5 KU/l or above. As a result, it was recognized that the exposure group had a high prevalence of PA induced allergic rhinitis. Also, in diagnosis of PA induced allergic diseases, medical histories and specific symptoms were the most important where as eosinophil count, serum total IgE are the only indirect informations. PA-specific IgE has been needed to evaluate the meanings more.

126: Korean J Prev Med. 1998 Nov;31(4):644-665. Korean.

A study on manganese health hazards among experienced welders.

This study was conducted to evaluate the health hazards and to develop early diagnostic methods of the manganism in experienced welders and to know the meaning of signal intensities on the brain Magnetic Resonance images. It was carried out from December 1996 to February 1997 with 277 male welders, the duration of welding was at least 5 years or more. The study was consisted of a questionnaire, physical examination and measurements of blood and urine manganese concentrations. Brain Magnetic Resonance imaging was done on 19 study subjects by random sampling. As the duration of welding increases, the positive rates of clinical symptoms, neurological examinations and blood manganese concentrations were also increased. However, physical examinations and urine manganese concentrations were not statistically significant with the duration of welding. Authors couldn't observe any Parkinsonism-like diseases. There were statistically significant correlations between duration of welding and blood manganese concentrations($r=0.16$, $p<0.01$). There were not statistically significant correlations between duration of welding and urine manganese concentrations($r=0.06$). There were statistically significant correlations between blood and urine manganese concentrations($r=0.34$, $p<0.01$). By viewing brain Magnetic Resonance images, 13 welders(68.4 %) among 19 welders were found to have signal intensities. The positive rates of clinical symptoms, physical examinations, neurological examinations and blood and urine manganese concentrations were not statistically different between those with signal intensities and those without signal intensities. We would like to suggest that some non-specific clinical symptoms and neurological signs are correlated with the duration

of welding but any Parkinsonism-like diseases had not been observed with these welders. Next we suggest that the high signal intensities on T1WI of brain Magnetic Resonance images are not the sign of manganese intoxication but the sign of manganese deposition.

127: Korean J Prev Med. 1998 Aug;31(3):414-423. Korean.

A Case of Metal Fume Fever Associated with Copper Fume in a Welder.

Metal fume fever has been known as an occupational disease is induced by intense inhalation of fresh metal fume with a particle size smaller than 0.5 nm to 1 nm. The fumes originate from heating metals beyond their boiling point, as happens, for example, in welding operations. Oxidation usually accompanies this process. In most cases, this syndrome is due to exposure to zinc oxide fumes; however, other metals like copper, magnesium, cadmium, manganese, and antimony are also reported to produce such reactions. Authors report a case of metal fume fever suspected to be associated with copper fume inhalation. The patient was a 42-year-old male and was a smoker. He conducted inert gas tungsten arc welding on copper-coated materials without safety precautions such as a protective mask and adequate ventilation. Immediately after work, he felt metallic taste in his mouth. A few hours after welding, he developed headache, chilling sensation, and chest discomfort. He also complained of myalgia, arthralgia, feverish sensation, thirst, and general weakness. Symptoms worsened after repeated copper welding on the next day and subsided

gradually following two weeks. Laboratory examination showed a transient increase of neutrophil count, eosinophilia, elevated erythrocyte sedimentation rate, and positive C-reactive proteinemia. Blood and urine copper level was also increased compared to his wife. Before this episode, he experienced above complaints several times after welding with copper materials but welding of other metals did not produce any symptoms. It was suggested that copper fume would have induced metal fume fever in this case. Further investigations are needed to clarify their pathogenic mechanisms.

128: Korean J Epidemiol. 1998 Jun;20(1):32-38. Korean.

A Case of Ulceroglandular Tularemia Occurred In Korea.

Tularemia is a zoonosis caused by Francisella tularensis. It is primarily a disease of wild animals. Human infection is incidental and usually results from interaction with biting or blood-sucking insect, wild or domestic animals or the environment. It is common in United States. An increasing number of cases have been reported from the Scandinavian countries, eastern Europe, Siberia, and Japan. But In Korea it has not been reported. A 40-year old male visited the department of Surgery on Jan 13, 1997 complaining multiple swollen lymph-nodes on his axillae and upper right arm for about ten days. On Dec 25, 1996, he found a dead wild rabbit at mountainside nearby, cooked it himself and ate it with his friends. He informed us that he got light injury on both hands while he was walking on

the mountainside. On Dec 28, he started to suffer from high fever, fatigue and loss of appetite lasting for a day. After medication at a local clinic for several day, symptoms were somewhat relieved. A week later(Jan 4, 1997), several erythematous lesions developed on his both hands, which left ulcerations on the skin. Both axillary lymph nodes were swollen at both sides, but not tender. He visited the department of surgery on Jan 13 and he admitted on Jan 15. During hospitalization, the lymph nodes were surgically removed from both axillae and upper left arm. On microbiologic examination, small aerobic gram negative coccobacilli were grown on the chocolate agar plate in aerobic condition with 5% CO_2 at 37 degrees centigrade. On Feb 10, fine needle aspiration from the liver abscess was done, drawing 3 ml of yellowish thick pustular material, but the microorganism was not isolated at the smear and culture of this material in the same condition as described above. After admission, he was treated with antibiotics(cefazole and marocin). His general conditions and laboratory results, including liver function, were markedly improved. He was discharged on Feb 12 and appears well on subsequent follow-ups. The microorganism and lymph nodes were sent to Centers for Disease Control and Prevention in the United States for further evaluation. A twostep indirect immunoalkaline phosphatase technique using an anti-F. tularensis antibody was performed on the lyph nodes having a positive reaction. The immunohistochemical stain demonstrated intense positivity in the stellate abscesses and fine granular reaction in some of the vessels in the paracortical region. Also F. tularensis was identified in the agar plug by culture morphology and immunofluorescence antibody test. We report a case of F. tularensis in Korea for the first time. Further studies were

recommened for epidemiological characteristics and prevention of the disease.

129: Korean J Occup Environ Med. 1998 May;10(2):290-298. Korean.

Three Cases of High Signal Intensity by Brain Magnetic Resonance Imaging in CO2 arc Welders.

We experienced three cases of high signal intensity observed by Brain Magnetic Resonance Imaging in CO2 arc welders of steel-frame manufacturing industry. Case 1 was a 35 years old man who has been an CO2 arc welder for 10 years, admitted a sudden onset of tonic clonic seizure. He complained fever, chilling and myalgia since 3 days before admission. On admission, in the test of manganese exposure indices, manganese concentrations of blood, urine and scalp hair were 5.17 microgram/dL, 22.00 microgram/l and 31.25 ppm respectively. Case 2 was a 35 years old man who has been an CO2 arc welder for 20 years. On admission, He complained fatigue, numbness and weakness of extremities, and decrease of libido. In the test of manganese exposure indices, manganese concentrations of blood, urine and scalp hair were 6.34 microgram/dL, 14.62 microgram/l and 57.87 ppm respectively. In neurologic examination, Palmo-mentis reflex and Myerson sign were observed. Case 3 was a 33 years old man who has been an CO2 arc welder for 16 years. On admission, He complainer loss of appetite, numbness of extremities, fatigue and decrease of attention. In the test of manganese exposure indices, manganese concentrations of blood, urine and scalp hair were 5.14

microgram/dL, 13.79 microgram/l and 50.08 ppm respectively. In neurologic examination, Myerson sign was observed. In brain magnetic resonance imaging, T1WI showed symmetrical high signal intensity in basal ganglia and midbrain of three cases. Authors argued that they were developed by manganese exposure, and we considered that follow up study would be necessary.

Publication Types:
Case Reports

130: Korean J Clin Pathol. 1998 Mar;18(1):90-95. Korean.

A Case of Tularemia Caused by Francisella Tularensis.

Tularemia is a major laboratory acquired zoonoses caused by Francisella tularensis that have high virulence, and usually transmitted to humans from direct contact with infected wild animals like rabbits or insect vectors like ticks. Clinical tularemia can be divided with 6 major syndromes that are delineated by the mode of organism aquisition, in which ulceroglandular type is the most common. F. tularensis have 3 different biogroups which have homogeneous antigenecity, type A (biogroup tularensis), type B (biogroup palearctica) and biogroup novicida, and can be confirmed by serology most frequently. In the domestic area, there was no reports of tularemia in humans or presence of bacteria in the reservoirs. Authers experienced a case of tularemia which is suspected as F. tularensis type B, ulceroglandular type. A healthy 40-year-old man admitted the hospital for

lymph node swelling in both axillary and upper arm area and for furuncles in both forearm and palm. He contacted with dead rabbit and eated it after cooking before 20 days from admission day. In laboratory cultures, F. tularensis did not grow in any of the routine or anaerobic culture media except for one blood agar plate at 5 days. After subculturing that to cystine containing chocolate agar plate at 37C degree, 5% CO2 incubator, we could see the accelerating growth of colony. In microbiological test, it was oxidase and urease negative. In acid production in cystine trypticase agar base, it was glucose positive and sucrose, maltose, glycerol negative. In agglutinating test, F. tularensis antiserum titer (Difco, USA) with isolates was 1:160 or over and antibody titer to F. tularensis antigen (Difco, USA) was 1:320 or over. Anti-F. tularensis-IF assay and Anti-F. tularensis-indirect-EIA with isolates were positive.

131: Korean J Occup Environ Med. 1998 Feb;10(1):121-127. Korean.

Chronic Subdural Hematoma Due to Cumulative Trauma to the Head.

Chronic subdural hematoma usually occurs as a consequence of minor trauma. But, chronic subdural hematoma of occupational origin has not been reported yet in Korea. We experienced a case of chronic subdural hematoma induced by repeated trauma to the head. The patient was a 45-year-old male. He complained of repeated headache and nausea. By the computed tomogram, he diagnosed as chronic subdural hematoma and took the operation. He was not an alcoholism and had no intracranial disease nor coagulopathy. We studied

all possibilities through working environment and personal environment survey. As a result, we decided the case as an example of cumulative trauma disorder to the head by occupational origin. We report a case of chronic subdural hematoma in the worker exposed repeated head trauma of occupational origin.

132: J Korean Acad Fam Med. 1998 Jan;19(1):95-106. Korean.

A Study on Bronchitis Associated with Welding at a Manufacture Factory.

BACKGROUND: The adverse health effects of welding come from chemical, physical, and radiation hazards. An outbreak of bronchitis was reported among welders at a manufacture factory in Pohang at Sep 1996. The object of this study is to assess the cause of the bronchitis in the welders.
METHODS: Authors conducted a questionnaire survey for 12 welders in the factory and a family physician examined their health status. Authors also collected their routine health examination reports and air concentrations of their work environment at that time.
RESULTS: The incidence rate of the bronchitis among welders was 91.7%(11 cases among 12 welders). The symptoms were developed at Feb 1996, 3 months after welding against steelplates. They were aggravated during worktime and relieved at weekend. The symptoms were cough(100.0%), sputum(90.9%), sore throat(72.7%), and general fatigue(72.7%). No abnormal findings were observed in the physical examinations and the radiologic findings. Though air concentrations of welding fume in the confined space(geometric mean =

14.68mg/m3) were over the permissible exposure limit(5mg/m3), those in open space(geometric mean=3.66mg/m3) were below. The air concentrations of metal components(Mn, Pb, Zn, Cu, Fe, Ni) in the fume were within each of the permissible exposure limit.
CONCLUSIONS: Authors thought that the outbreak of bronchitis was brought about by CO2 arc welding against paint-free steelplates. Further studies on the factors contributing to the bronchitis will be needed.

133: Korean J Pathol. 1998 Jan;32(1):68-71. Korean.

Granulomatous Inflammation of Hand following Sea Urchin Sting: 2 cases report.

Injuries from sea urchins are induced by from penetration of the calcareous spines into the skin. Apart from the transient episode of excruciating pain, there is usually no residual disability. Complications arise, however, when spines are embedded over bony prominences, or within joints. Two cases are reported with injury and protracted disability of fingers resulting from contact with the purple sea urchin, Anthocidaris crassispina, a common echinoderm inhabitant of the Korean east coast. After a latent period of several months in both cases, Case 1 presented as caseating granulomas in the synovium and case 2 exhibited as the usual soft tissue nonsynovial foreign body and noncaseating granulomas. There appears to be a paucity of published data regarding the effects of puncture wounds caused by the spines of this animal. The granulomas have appeared after a

latent interval of several months in a proportion of the sufferers, suggests a delayed hyperserisitivity reaction similar to that produced by Mycobacterium species.

134: Korean J Prev Med. 1997 Dec;30(4):805-814. Korean.

A Case Report on the Meniscal Tear due to Repetitive Foot-Switch Stepping.

Injuries to the menisci occur in a variety of ways, most commonly with a twist, pivot, squat, or valgus stress to the knee. Tear patterns are classified to longitudinal, horizontal, or transverse features according to the mechanism of injury. Work-related meniscal tear usually occurs with a repetitive usage of the foot, hence it can be classified as a cumulative traumatic disorder. We found a 47 year-old female worker who had been taking charge of repetitive foot-switch stepping for 8 years. She suffered from pain in the right knee since 5 months ago. Tenderness along the medial joint line of the right knee was observed and pain was aggravated with full flexion of the right knee. On magnetic resonance imaging, high signal intensity was observed at the posterior horn of the medial meniscus of the right knee. Degenerative longitudinal and transverse complex tear in the medial meniscus was observed on arthroscopy. Arthroscopic partial meniscectomy was performed. We surveyed the work process and the health status of co-workers. It turned out that the work process was compatible to injure the meniscus and nine out of fourteen co-workers(64.3%) complained pain of the knee. No other factors related to her meniscal tear could be

found except for the situation at her work. Therefore, we conclude that meniscal tear is related to the repetitive stepping of foot switch.

135: Korean J Prev Med. 1997 Dec;30(4):752-763. Korean.

Respiratory symptoms of workers exposed to the fume containing manganese.

To evaluate the effect of manganese on the respiratory system, we investigated the respiratory symptoms of 63 male workers exposed to fume containing manganese (Mn), iron (Fe), and silica (Si), and compared them with those of 66 male workers not exposed to the fume in a manganese alloy smelting factory. The prevalence ratios of the seven respiratory symptoms were not different between two groups. The presence of any respiratory symptom was not related with the age, duration of employment, smoking status of workers, and exposure to fume. In furnace workers, it was not related with the airborne Mn, Fe, and Si concentration in the total or respirable fume. Airborne Mn concentrations of all 4 furnaces in the respirable fume were below 1 mg/m(3). There were two suspicious cases of pneumoconiosis among furnace workers and one definite case(1/2) among casting workers who were not exposed to fume. The above results suggest that the exposure to the low airborne Mn concentration is not related with respiratory symptoms and pneumoconiosis. However, it is necessary to study the respiratory effects of Mn using the symptom questionnaire with consideration of the severity and persistence of symptoms and the time interval from exposure.

136: J Korean Acad Fam Med. 1997 Aug;18(8):855-865. Korean.

Musculoskeletal Symptoms and Ganglions Developed in Repetitive Job Workers.

BACKGROUND: Musculoskeletal symptoms, such as pain, numbness, and ganglions on involved joints were common problems in repetitive job workers, who exposed to prolonged, repetitive use of the wrist in factory. This study was performed to compare the degree of musculoskeletal symptoms and prevalence of soft tissue mass (esp. ganglion on wrist) in repetitive job group with those of non-repetitive job group and to evaluate the association of development of ganglion and the duration of wrist use.
METHODS: Study subjects were 253 workers who work on a brick manufacturing factory and they were composed of 153 non-repetitive job workers and 100 repetitive job workers. Authors conducted a questionnaire survey among workers in the factory and examined their lesions.
RESULTS: There were no significant difference statistically in age, level of education and work duration in both groups. It was statistically significant that the degree of wrist use was more frequent in repetitive job group than in non-repetitive job group($P<0.01$). Musculoskeletal symptoms, such as pain and numbness on affected joints were more common in repetitive job group than in non-repetitive job group. Affected side was bilateral in three joints and right only in two joints($P<0.01$, $P<0.05$). The prevalence of ganglions in repetitive job group(6 subjects, 6.0%) was much higher statistically than that in the non-repetitive job group(2 subjects,

1.3%). In a comparison of two groups according to the existence of ganglions, no stasistically significant differences were found in age, level of education and work duration. However, duration of wrist use was statistically significant relation with development of wrist ganglions($P<0.05$). The 8 subjects with ganglion on wrist all worked on manufacturing part in the factory. The duration of work ranged from two to fourteen years. The affected sides of ganglions on wrist were right in 3 cases and left in 5 cases. Size of ganglions ranged from 0.5 to 3 cm in diameter. Painful symptom in the ganglion was complained by two cases(25%) among 8 cases.
CONCLUSIONS: We suggest that prolonged, repetitive activities such as carrying a brick, have a tendency to develop musculoskeletal symptoms and ganglions in the workers. Because high prevalence of ganglions in this factory seems to be an important occupational problem among repetitive job workers, further studies on the factors contributing to development of ganglions in this factory are needed.

137: Korean J Occup Environ Med. 1997 Jun;9(2):283-291. Korean.

A Case of Occupational Asthma Associated with Chromium.

Occupational asthma is an important occupation-related disease. We experienced a case of asthma induced by chromium. After discovery, chromium has been widely used in industry. Health effects of chromium and its compounds are contact dermatitis (allergic and irritant), mucosal

irritation, basal septal perforation, asthma, and lung cancer. Their toxicity depends on valency, solubility, concentration, pH, exposure duration and route. The patient was a 47-year-old male and an ex-smoker. He complained of cough, sputum and dyspnea while working. Total eosinophil count was elevated but serum IgE value was normal. Bronchoprovocation test with 0.5 % (w/v) chromium solution revealed early and late asthmatic responses. Although his initial methacholine bronchial challenge test shoved a negative result, bronchial hyperresponsiveness developed after bronchoprovocation test with chromium solution. After discharge, he has complained of an intermittent dyspnea. It was suggested that chromium could induce occupational asthma in exposed workers. Further investigations are needed to clarify their pathogenetic mechanisms.

138: Korean J Prev Med. 1997 Mar;30(1):145-156. Korean.

Phototoxic Dermatosis among Coal-tar Pitch Workers.

Interview survey and dermatological examination have been performed to investigate the health problems of workers continuously exposed to coal-tar pitch. The phototoxicity of coal-tar pitch was confirmed by the photopatch tests for six healthy adults. The main results are followings;

1. There was no special history of allergic diseases in both the exposed and non-exposed group.
2. The frequency of the phototoxic dermatosis and the coal-tar acne in the exposed group was significantly greater($p<0.05$) than that of the control

group. In the exposed group, the phototoxic dermatosis and the coal-tar acne were observed in nine workers(90%) and seven workers(70%), respectively. However, those disease were not observed in the control group.

3. Five results(83%) were positive to the photopatch test for coal-tar pitch 48 hours after UVA irradiation. But the lesion was subsiding 72 hours after UVA irradiation.

4. Malignant cancers were not reported among workers ever exposed to coal-tar pitch.

In conclusion, it appears that workers exposed to coal-tar pitch have high risks of phototoxic dermatosis and coal-tar acne. A health policy should be provided to prevent phototoxic dermatosis among coal-tar pitch workers. More studies are required to determine malignancy.

139: Korean J Occup Environ Med. 1997 Feb;9(1):140-155. Korean.

Cumulative Trauma Disorders among Telephone Directory Assistance Operators.

To evaluate the prevalence of cumulative trauma disorders in relation to ergonomic evaluation of their work, 260 female directory assistance operators employed in three branch facilities of the nation-wide telecommunication company. Ergonomic evaluation of the work status was done by two industrial hygienists through inspection. Workers were surveyed with standardized self-administered questionnaire and examined by family physicians and an orthopaedician for cumulative trauma disorders.

Laboratory examination of serum aspartate aminotransferase(AST), creatinine phosphokinase(CPK), aldolase and rheumatoid factor was done and X-ray of cervical spine and both shoulders was taken from all subjects and nerve conduction velocity test was done on 57 subjects with suspected nerve entrapment cases. Cumulative trauma disorders were categorized into 3 category according to work-relatedness of the disorders. On ergonomic evaluation of the working posture and work environment, improvement of work posture revealed inappropriate postures and devices. Mean age of the subjects were 39 years old and more than 80% graduated high school or above. Ninety-eight percent of the workers employed in the company for more than 15 years and 74.6% of the workers worked at the VDT job for more than 4 years. Mean daily work hours was 8.0 hours per day and mean break hours was 90.5 minutes per day with mean extra work hours of 10.7 hours per month. Mean daily calls were 1128.6 calls in maximum and 864.8 calls in minimum. On laboratory examination, 13 (5.0%) workers had abnormal AST, 53(20.4%) had abnormal CPK, and 1 (0.4%) had abnormal aldolase level. On x-ray examination of cervical spine, 111 (42.7%) had loss of or reversed normal cervical lordosis, 10(3.8%) had narrowing of intervertebral space. Nerve conduction velocity test was abnormal in 11 (19.2%) among 57 workers. Among disorders categorized as work-related, 131 (50.4%) were diagnosed to have fatigue myalgia of levator scapulae, 108 (41.5%) had cervical strain or sprain, 47 (18.1%) had fatigue arthralgia of phalangeal joints, and 47 (18.1%) had probable carpal tunnel syndrome, in 10 (3.8%) of those diagnosis was confirmed electrophysiologically. Age, seniority, work duration at present job, workload nor location of facilities were associated with the prevalence of symptoms nor specific diagnoses on simple

statistical analysis and multiple logistic regression, which deems to be due to highly selected and homogeneous characteristics of the study subjects. Above results shows that cumulative trauma disorders are very prevalent among directory assistance operators and elaborate effort is needed to reduce the cumulative trauma disorders among the operators.

140: Korean J Prev Med. 1996 Sep;29(3):617-638. Korean.

A Study on the Establishment of Management Methods about Occupational Dermatoses.

Occupational dermatosis is one of the most prevalent occupational disorders. However, the extent of the occupational dermatoses including incidences and prevalencies of each disease entity, and etiologic materials are not yet well stated in Korea. Authors reviewed the literatures on the statistic data and reports on the occupational dermatoses, and surveyed on the occupational dermatoses in two factories, and surveyed the physicians responsible to the occupational dermatoses with formed questionnaire. The results are as follows; 1. Among medical journals published since 1964, there were 31 articles on the occupational dermatoses. Of 31 articles, 18 were case reports and all others were review articles. Of 18 case reports, 9 were epidemiologic survey. The Workers' Periodic Health Examinations revealed that prevalence of the occupational dermatoses was highest(4.36 per 10,000 workers) in 1974, but number of the cases reported were decreased sharply since 1978 with some tendency to increase since 1987.

There were 2,240 reported cases of occupational dermatoses between 1966 and 1992, which is 1.90% of all the reported occupational diseases. Skin infection and injuries due to chemicals were most frequent and there were 6 cases of skin cancer. 2. In an epidemiological survey on the dermatoses among 995 workers in a metal product manufacturing factory and 225 workers with acne, 130 workers with scar, 123 workers with deformity of toe nails. Scars, photosensitivity dermatitis, deformity of finger and toe nails, and acne were more prevalent in the metal product manufacturing factory($p<0.05$). In the metal product manufacturing factory, workers treating organic solvents and oils had more dermatoses than those without treating the materials($p<0.05$). On the skin patch performed on 16 workers in the metal product manufacturing factory, there were 8 cases of irritation dermatitis and 5 cases of contact dermatitis. Prevalence of contact dermatitis in the metal product manufacturing factory was 1.3%. 3. On the questionnaire survey, 34 dermatologists, 29 doctors of preventive medicine, and 22 family physician replied. The proportion of occupational etiology among all dermatoses assumed by the physicians were below 9%, and the most important occupational dermatosis in Korea was contact dermatitis. Main etiologic materials related to the occupational dermatosis were organic solvent, acid and alkali, and metals. The reason for the scarcity of report of occupational dermatoses were difficulty in diagnosis and physician's ignorance of the occupational etiology. They replied that to prevent the occupational dermatosis in the workplace, the use of protective devices was most important, and development of diagnostic criteria on the occupational dermatoses is urgent. Above results shows us that there is many workers with occupational dermatoses, but they are mostly unreported.

Measures to prevent and manage the occupational dermatoses are not satisfactory at present. Hence, authors suggest measures for the precises diagnosis, report and prevention of the occupational dermatoses. a. Dernatikigustm orevebtuve physician, and industrial hygienist should work as a team to examine the high risk group and establish the preventive measures. b. Disease entities, diagnostic criteria of occupational dermatoses should be listed, criteria for the compensation and job fitting at recruitment should be established, and manual for the proper treatment and effective prevention of each occupational dermatosis should be developed. c. Patch test antigens against each occupational category should be developed and it should be available to any physicians responsible. d. To facilitate the diagnosis of occupational dermatoses by the doctors responsible for the Workers' Periodic Health Examination, development of standardized questionnaire, education on the techniques of the patch test, and cooperation with the dermatologist in diagnosis of occupational dermatoses is essential.

141: Korean J Prev Med. 1996 Mar;29(1):113-132. Korean.

A Survey on Health Behavior of Male Workers in Steel Industry.

This study was carried out to evaluate the relations among workers' general characteristics, work-related behaviors and health related behaviors in a steel industry with 1,134 workers in Pohang. The results were as follows;
1. The mean age of workers was 50 years old and working duration was 15

years and over. Most of them were married(94 5%) and had studied beyond high school (53.0%). They performed three shift work and most of them(63.0%) had experienced industrial accidents. The frequency of noise and dust exposure was defined by a minimum of 6 hours per day, and workers complained about noise exposure(62.9%) and dust(55.6%). There were current smokers(67.7%), current drinkers(74.3%) and current exercising workers(32.3%) in the industry. The number of cigarette consumption in current drinkers was significantly high (13.6 +/- 8.4 pieces/day) and the alcohol consumption in current smokers was significantly high(l04.5 +/- 113.5 g/wk). And the number of cigarette consumption of exercisers was significantly low and the alcohol consumption of exercisers was higher than non-exercisers. 3. The ratio of current smokers on frequent noise in exposed workers versus non-exposed workers was not significantly high but the current drinkers in frequent noise and dust exposed workers was significantly higher than non--exposed. 4. The normal levels of SGOT workers in non-smokers were significantly higher than in current smokers, and the abnormal levels of LFT(SGOT, SGPT, gamma-GTP workers in nondrinkers were significantly high. The normal levels of SBP and DBP workers in current smokers were not significantly high but were significantly high in non-drinkers. 5. The ratio of current smokers in workers unsatisfied with their job and working condition was higher than non-smokers, but the ratio of current drinkers in workers satisfied with their job and working condition were higher. 6. The significant factors for SGOT and gamma-GTP were age, the drinking amount and BMI. But the only significant factor for r-GTP was BMI. The significant factors for DBP were age, the alcohol consumption and BMI And the significant factors for SBP were age, BMI.

142: Korean J Occup Environ Med. 1995 Oct;7(2):332-346. Korean.

A Study on the Status of Preemployment Health Examination.

Preemployment health examination, stipulated by the Occupational Safety and Health Regulations, plays an important role in workers' health management. Recently, employers are getting more aware of the importance of screening recruits before employment to decrease the development of occupational disorders and work loss due to health problems. Accordingly, additional test items are being included and selection criteria for preemployment health examination is getting more strict. However, studies and. data on preemployment health examination are scarce until now. Authors reviewed preemployment health certificates of 3,261 recruits issued by a university hospital in Pohang from January 3 to December 31, 1993, analyzed regulations on preemployment health examination of 16 companies in the Pohang area, and surveyed 80 health and safety personnel with questionnaire to evaluate the present status of preemployment health examination. Of 3,261 recruits 125 (3.8 %) failed to recruit. Main causes of. failure were chronic liver disease, tuberculosis of undetermined activity. The regulations of 16 companies on preemployment health examination were reviewed. Most companies adopted additional test items in addition to basic test items listed on the Regulation; test of physical strength, pulmonary function test, pure tone audiometer (2000 Hz and 4000 Hz in addition to 1000 Hz), exercise provocation test, urine erythrocyte and urobilinogen,

complete blood count, stool examination, VDRL, arid sputum examination for acid fast bacilli In most companies, obesity, hearing disturbance, color vision disturbance was included in the selection criteria. Hepatitis B carrier was not accepted in 4 companies, accepted conditionally in 2, and accepted in 5 companies. Of 80 health and safety personnel of companies in the Pohang area, 56 (70. 0 %) perceived preemployment health examination as a tool for selection of recruits and 14(17.5%) as a procedure for job fitting. Most of them were against the acceptance of recruits with hepatitis B carrier, lumbar spine deformity, and hearing disturbance. In conclusion, preemployment health examination is performed mainly for the purpose of selection of healthier employee, but test items and selection creteria is not seem to be adequate. Authors suggest that further studies are needed to select the test items for specific job, and application of selection criteria be restricted only to some specified jobs. Consecutive filling of preemployment health examination to the periodic health examination was also suggested.

143: Korean J Prev Med. 1995 Sep;28(3):551-562. Korean.

A Study on Safety Accidents Occurred for 5 Years at a Welding Material Manufacturing Factory.

To assess the status of safety accidents, authors reviewed and analysed the records of safety accidents of a welding material manufacturing factory at pohang city from January 1989 to December 1993. The results are; 1. The

total incidence of safety accidents was 295 spells for five years. 2. Average age of workers with accident was 35.7 years. Average duration of employment was 6.2 years and the duration of employment increased as the year increased. 3. There was no statistical significance on season, month, weekday and time by year in the incidence of safety accidents. The most frequent part of body injured was upper and the most frequent type of injury was abrasion. 4. Mean admission rate of safety accidents was 12.6% and the ratio of treated spells as occupational injury was 7.8%. 5. The most frequent cause of injury was worker's mistake and the most frequent action for the prevention of further accidents was safety education. 6. The incidence rate of safety accidents on 1993 was 116.2 spells per l,000 persons. Above results suggest that to prevent safety accidents, safety education should be done continuously, the environmental and human factors were controlled and more exact reporting system of safety accidents was needed.

144: Korean J Epidemiol. 1995 Jun;17(1):76-93. Korean.

An Epidemiologic Study on the Health Hazards of Inhabitants chronically exposed to Glass Fiber.

Fiberglass, as a substitute of asbestos, is used for more than 60 years as a insulator material. Health hazards including irritation of skin, mucosa and respiratory system associated with use of fiberglass is reported. Many studies on the fibrogenicity and carcinogenicity of fiberglass was

conducted but evidence is not sufficient to confirm the carcinogenicity or fibrogenicity. Authors studied the health hazards among 152 inhabitants(71 men and 81 women) from 32 households living around the fiberglass factory which produced the fiberglass insulators and glasswool panels for 20 years. Questionnaire survey on household and persons, examination of underground water, pathologic examination of subcutaneous tumors and examination of fiberglass in tumor tissues were done. The results are as follows; 1. Fiberglass concentration of underground water sampled from 33 households in the study area was 13. 7-95. 9 fiber/cc with the diameter to length ratio more than 1:20. 2. Prevalence of dermatosis among study subjects was 23.0 % (35 cases). Prevalence was not associated with the distance from the factory nor duration of exposure. 3. There were 15 cases of subcutaneous tumor with prevalence of 9. 9 %. Age of subcutaneous tumor cases was all above 30 year-old except one cases, who was 5 year old child, who lived in the surveyed area since he was born. Prevalence of subcutaneous tumor was significantly high in area A(42.9 %) than area B(4.6 %, $p<0.01$). Prevalence of subcutaneous tumor was higher in long-term exposed inhabitants, but was not statistically significant. 4. Pathologic examination exhibits partly encapsulated fat tissue masses and cut surfaces were pale yellow with gritty sensation. The masses consist of mature fat cells showing variation in size and shape. On polarizing microscope, the peripheries of tumors include small irregular threads of doubly refractile material probably represent glass fibers in fibrocollagenous tissue. Concentration of fiberglass in tissue was 5.1-10.2 fiber/rag wet tissue in case 1, 25.8-184.9 fiber/mg wet tissue in case 2 and 40.8-126.5 fiber/mg wet tissue in case 3. Length of fiberglass was shorter than that in underground water.

5. Cases of malignant tumor among inhabitants since last 10 years were 4, 3 of whom was developed in a same household just near the factory. Diagnoses of malignancy cases were stomach cancer, stomach and esophageal cancer, oral cavity cancer, and stomach cancer with liver metastasis. On review examination of tissues of endoscopic biopsy specimen from a case of stomach cancer, there was adenocarcinoma with no evidence of fiberglass materials. Authors concluded there is evidences that fiberglass was strongly associated with the development of the health hazards including dermatosis and benign subcutaneous tumor. However, the association of fiberglass exposure with the development of malignant tumor was not clear, although strongly suggested. For the prevention of development of further health hazards, it is recommended that under ground water source should be closed and further experimental study to confirm the mechanism of the tumorigenesis and follow up survey on the inhabitants should be conducted.

145: Korean J Prev Med. 1995 Jun;28(2):406-420. Korean.

A Study on the Manganese Exposure and Health Hazard among Manganese Manufacturing Woman Workers.

No abstract available.

146: Korean J Prev Med. 1995 Mar;28(1):13-26. Korean.

An Epidemiologic Study on Occupational Dermatosis Associated with Mites.

An outbreak of dermatosis occurred in a livestock fodder factory at Kyongju in May 1994. Authors conducted a questionnaire survey on 60 workers in the factory and a dermatologist examined their skin lesion. Authors also collected mites and identified them. The obtained results were summarized as follows; 1. Twentyeight cases of dermatosis were identified with attack rate of 46.7%. Attack rate was not different by department, age, sex, educational level and employment duration. Attack rate was 67.5% in productive worker and 5.0% in clerical workers ($p<0.01$) but was not significantly different between departments among productive workers ($p>0.05$). 2. Three cases among 28 dermatosis cases and one subject among 32 non-cases had a history of same dermatosis last year. Only one of dermatosis cases had a history of dermatosis among family members. History of other skin lesion and allergy was very rare in both cases and non-cases. 3. Skin lesions of the cases were rice-sized erythematous papules or vesicles with a central biting point. Onset date of dermatosis was between May 1 and June 10. Duration was from one day to more than 30 days. Skin lesion was most frequent at the back(75.0%). and also observed at the arms(64.3%). abdomen(60.7%), legs(57.1%), chest(32.1%) and neck (25.0%). Skin lesion was aggravated while workers are sweeping the floor(35.7%), working at the workplace(21.4%) and in bed at night(28. 6%). 4. Total l,637 mites were collected and identified into 3 suborder, 7 families and 17 species. Dermafophagoides farinae was most frequent Nnd most of the species identified were blood sucking mites. Authors concluded that the outbreak of dermatosis was brought about by mite-bites and grain beetle parasitizing

Acarophenax tribolii was the most suspected species. Further studies to identify the specific species causing dermatosis and route of import are needed.

147: Korean J Occup Environ Med. 1995 Feb;7(1):120-127. Korean.

A Study on the Change of the Bone Density among Workers Exposed to Hydrofluoric Acid.

Hydrofluoric acid is one of the strongest irritating, corrosive and poisonous inorganic chemicals. The most significant chronic consequence of excessive fluoride exposure is the disorder of skeletal system and connective tissue. The first stage of osteofluorosis consists of an increase in the density of flatbone such as pelvic bone and vertebral bones, with coarseness and blurring of bone trabecular. Therefore we wanted to observe the change of the bone density among workers exposed to hydrofluoric acid. Questionnaires and radiological investigations were performed for 39 hydrofluoric acid manufacturing factory worker sexposed to hydrofluoric acid. And then authors checked urinary fluoride level by fluoride ion method. the radiographs were doubly read by two radiologists for reliability.

The results were as follows;

1. There was a significant difference in urinary fluoride level ($p<0.01$), but not in age, in tenure($p>0.05$) among workers by the job title.

2. By the two radiologists, eight cases were read as bone fluorosis. The

prevalence rate of bone fluorosis was 20.5%.

3. There was not a significant difference in general characteristic such as age, tenure and in muculoskeletal signs and symptoms between the fluorosis group and the normal group.

4. However, a close relationship between the occurence of the change of bone density and the degree of fluorde exposure such as burn was found. The difficulties in diagnosing fluorosis result from the questionable sensitively of x-ray techniques and from the non-specific associated signs and symptoms. A quantitative method such as densitometry to assessosteosclerosis and bone structure alteration and a follow-up study are needed.

148: Korean J Occup Environ Med. 1995 Feb;7(1):21-27. Korean.

Validity and Reliability of Data Derived from Questionnaire on Neurobehavioral Symptoms.

There are many studies on the neurobehavioral symptoms due to organic solvent exposure using questionnaire. However there is little published evidence on validity and reliability of the questionnaire on the neurobehavioral symptoms. In present study, the authors tested the validity and reliability of our questionnaire, which was designed for screening neurobehavioral disturbance in organic solvent exposer. Questionnaire was administered to the workers of one paint manufacturing plant and one coil manufacturing plant. In order to evaluate validity of the questionnaire,

the average questionnaire scores of two plants were compared. The average score of paint manufacturing plant were higher than that of coil manufacturing plant and the difference was statistically significant. After adjustment of age, duration of employment, education, smoking history and alcohol consumption, significance was maintained. Test-retest reliability was evaluated by kappa statistics. More than 50% of question items showed values of kappa above 0.4, Cronbach coefficient alpha whoch reflects internal consistency of the questionnaire was 0.86. Overall the data showed that validity and reliability of the questionnaire were generally acceptable.

149: Korean J Prev Med. 1994 Dec;27(4):747-762. Korean.

A Baseline Study on the Choice of Optimal Screening Test Items among Workers with Abnormal Liver Function Tests on Workers' Periodic Health Examination.

Workers', periodic health examination is the main tools used to manage the health problems of most workers in Korea. The most common health problem found in workers' periodic health examination is liver disorder. Liver disorder is also one of the most common health problems in general population and one of the leading causes of mortality in adult population. Regulation proposed by government(NO. 207, Ministry of Labor, 1992) defines the criteria for selection of workers with the liver dysfunction for further evaluative examination and the examination items used for diagnosis

of the workers with liver dysfunction. This study was designed to evaluate the proficiency of each examination items presently defined in Regulation and propose the optimal examination items for detection of the liver disorders found by workers' periodic health examination. Study subjects are 186 workers with abnormal liver function tests in screening examination of workers' periodic health ex amination. Questionnaire survey including past history of liver disorder, drinking history, height and weight was done. Physical examination by physician, routine test items defined by Regulation (SGOT, SGPT, gamma- GTP, protein, albumin, total and direct bilirubin, alkaline phoshatase, alpha-feto protein, HBsAg and anti-HBs), anti-HCV antibody test and liver ultrasonography were done. Results are as follows; 1. Result of evaluative examination utilizing only the items defined in Regulation was; There were 75 workers with suspected liver disorder(40.3%), 63 with no liver dysfunction(33.90%), 13 with suspected hepatitis B(7.0%), 10 workers with hepatitis B(5.4%) 10 workers with hepatitis B carrier state(5.4%), 10 with alcoholic liver disorders(5.4%), 5 with fatty liver(2.7%). When alternative diagnostic criteria applying additional examination items (drinking history, body mass index, anti-HCV antibody and ultrasonography) diagnosability of liver disorder was increased. When all four items were included, final results were; 23 workers (l7.8%) with hepatitis B(10 carriers, 13 suspects and 10 hepatitis B), 10(5.4%) with hepatitis C(4 carriers, 5 suspects and 1 hepatitis C), 13(7.0%) with alcoholic liver disorder, 45 (24.2%) with fatty liver (40 suspects, 5 fatty liver), 41 0%) with suspected liver disorders and 44(23.7%) with normal liver. 2. Of examination items defined by Regulation, only SGOT, SGPT, gamma-GTP and HBsAg were significantly different in abnormal rate and mean

value, and all other laboratory findings did not showed significant difference between two groups. Drinking history, body mass index and anti-HCV antibody test which are the items that authors included in this study, also showed significant difference between two groups. Utilization of body mass index(BMI) for abnormal liver function group in diagnosis of fatty liver had high specificity(97.6%) but sensitivity (22.3%) was low. Therefore we suggest that SGOT, SGPT, gamma-GTP, HBsAg, alcohol drinking history, BMI and anti-Hcv Ab were useful for diagnosis of liver disorders among worker's periodic health examination.

150: Korean J Prev Med. 1994 Dec;27(4):693-710. Korean.

An Epidemiologic Investigation on an Outbreak of Anthrax Occurred in Kyongju by Eating Dead Cow's Meat.

This epidemiologic study was carried out to investigate cause and magnitude of food-poisoning like epidemic occurred among inhabitants of a village who have eaten dead cow's meat near Kyonng in February of 1994, around lunar new year. The investigation consisted of interview survey on all inhabitants of 77 households (l11 males and 119 females) and their visitors (40 males and 35 females), skin test with anthraxinum(Russian product), study on clinical characteristics for the patients hospitalized, and microbiologic examination on microbes isolated from cow's meat, patient and soils of dead cow's barn. The results obtained are as followings; 1. The proportion of the inhabitants who ingested the dead cow's meat was 36.4%.

The incidence rate of the disease was 65.1% for males, 41.7% for females and the cases were distributed evenly for all age groups. The group ingested raw meat showed higher incidence than the group ingested cooked meat. There was no case among people who did not eat the meat. 2. The most clinical symptoms were significantly more frequent among cases than non-cases; sore throat (57%), nausea (51%), fever(47%), indigestion(43%), cough(41%), anorekia(41%), abdominal distention(41%), and abdominal pain(39%) were the major symptoms among cases. 3. Among 29 cases hospitalized out of total 61 cases, three patients, all old and feeble persons, deceased from the disease resulting in 4.9% fatality rate among total patient and 10.3% among hospitalized. Septicemia and meningitis were the causes of the deaths. 4. Three strains isolated from patients, and three strains from dead cow's meat and soil revealed typical microbiologic characteristics of Bacillus Anthracis, which also proved to be fatal to experimentally infected mice.

151: Korean J Occup Environ Med. 1994 Sep;6(2):439-446. Korean.

A Case Report on Glass Fiber-induced Health Hazard due to Car interior Material.

Glass Fiber is manufactured from typical glass-making raw materials, silicon dioxide with various metals and other elements. There are three distinct types of commercial glass fiber products: glass wool, an entangled mass of interlocking fibers; continuous glass filament, a product with a

more ordered arrangement of fibers; and special-purpose glass fiber, a small-diameter fiber (less than 3 um) .

We report a case of glass fiber-induced health hazard due to continuous glass filament from fiber reinforced plastics (FRF) as car interior material. A 40 years old man complained a sudden onset of severe itching on whole body, especially neck and forearms while driving a new car. He also complained burning of eyes, sore throat and productive cough.

We diagnosed the glass fiver-induced skin lesion by placing skin scraping on a slide glass and examining the specimen under a microscope. We found same fibers from the air samples in the car and from the material of the interior surface of the car by the microscopic examination. Therefore we confirmed that the patient's symptoms and signs were induced by glass fiber.

152: Korean J Occup Environ Med. 1994 Feb;6(1):98-112. Korean.

A Study on the Manganese Exposure and Health Hazards Among Manganese Manufacturing Workers.

To estimate the manganese (Mn, below) exposure of workers in the ferromanganese manufacture factory and to evaluate its health effects, airborne, blood and urine Mn concentration measurements, questionnaire and other neurologic examinations were performed on 80 Mn-handing productive male workers (exposed group), 47 non-Mn-handling productive male workers (internal control group) and 144 productive male workers in other factory

(external control group).

The results obtained were as follows;

The highest airborne Mn fume concentration among the work process was found at charging (0.42 mg/m3), and ferromanganese crushing process (1.14 mg/m3) was the highest in Mn dust. However all of them were below threshold limit value. Mean Mn concentrations in blood and urine of crushing workers were higher than those of other part workers. Among all of them, workers whose urine Mn concentration were exceed normal reference level (10 microgram/l) were 31 (18.5%). There was statistically significant correlation between airborne and urine Mn concentration (r=0.60), and so between airborne and blood Mn concentration (r=0.49), while there was no statistically significant correlation between blood and urine Mn concentration. Mean Mn concentration in airborne (0.60 mg/m3), urine (6.92 microgram/l) and blood (3.16 microgram/dl) in exposed group were significantly higher than those of control groups (p<0.01). Clinical symptoms such as excessive sweating, hypoesthesia, libido change, anosmia, decreased visual acuity and difficulty in writing showed higher positive rate in exposed group.

Positive rate of clinical signs such as eye blinking and masked face in exposed group was higher than external control group. However clinical and laboratory findings such as blood pressure, blood chemistry, grip strength in exposed group were not statistically different from those of control groups.

The results suggested that further studies were followed to evaluate the workers whose blood Mn concentration were below normal reference level but urine Mn concentrations above normal reference level, and to establish the questionnaire and the diagnostic tools to detect the Mn poisoning workers

early.

153: Korean J Prev Med. 1993 Dec;26(4):587-598. Korean.

A study on occupational hydrofluoric acid burns in a hydrofluoric acid manufacturing factory.

Hydrofluoric acid is one of the strongest irritating, corrosive and poisonous inorganic chemicals. Hydrofluoric acid burns are occurring with ever-increasing frequency due to the wide use of this acid in industries. Hydrofluoric acid burns are characterized by severe progressive tissue destruction and excruciating pain due to the unique properties of the freely dissolvable fluoride ion. The authors reviewed medical records of 32 cases(36 spells) of hydrofluoric acid burns which occurred in a hydrofluoric acid manufacturing factory from Sep. 1, 1990 to June 30, 1993. The results are as follows; 1. Eleven measurements of air concentrations of hydrofluoric acid by detection tube method from 1990 to 1992 were all below TLV(Department of Labor, R. O. K). 2. There were 19 cases(22 spells) of hydrofluoric acid burns which occurred during the study period among regular employees. The overall incidence density of hydrofluoric acid was 17.8 cases(20.6 spells) per 100 person-year. Incidence density was 19.0 cases(22.0 spells) per 100 person-year among male workers and there were no female cases. Incidence density was 32.9 cases(38.3 spells) per 100 person-year among production workers and 1.9 cases(1.9 spells) per 100 person-years among management workers with the difference being

statistically significant(P<0.01). 3. Of 32 cases(36 spells) of hydrofluoric acid burns among workers who were regularly employed or temporarily employed, 26 spells(81.2%) were between age 20 to 39. In 15 spells(41.7%) burns occurred between 12 : 00 and 17 : 59 with 16 spells(44.3%) having arrived at hospital within 2 hours after the accident. 4. Of 36 spells, the main cause of hydrofluoric acid burns were by splashes(8 spells, 22.2 %). The most frequent site of burns were fingers and pain was the most frequent symptom. Thirty spells(83.3%) of the hydrofluoric acid burns were treated with local injection of antidote(calcium gluconate). Complete recovery without scarring were observed in most of the cases(34 out of 36 cases, 94.4%). The study results suggest that to prevent hydrofluoric acid burns, environmental control and the wearing of hydrofluoric acid resistant protective clothes and gloves are important. It is also stressed that establishment of an emergency management and a transfer system for hydrofluoric acid burn victims is necessary.

154: Korean J Prev Med. 1993 Dec;26(4):480-507. Korean.

Determinants Of Health: Environmental Factors.

Environmental pollution is common problem of the present world that is intimately related to the future survival of human beings. The problems of environmental pollution originate from the pursuit of benefit by enterprises, insufficient countermeasure of government and ignorant life

style of the people. Health hazards due to environmental pollution have characteristics of irreversibility, difficulty in measurement and ineffectiveness of personal prevention. Objects of this article are to review the various aspects of environmental pollution, to outline the present status of environmental pollution and strategy to control environmental pollution in Korea. In the first part of this article, causes of environmental pollution are presented. International relationships, world-wide status of environmental pollution and health hazards due to environmental pollution are briefly reviewed. In the second part, present status of air, water, soil and ocean pollution in Korea is presented. Pollution by radioactive materials, noise, vibrations, odor, wastes and chemicals is reviewed. Climate changes related to environmental poisoning, problems of workplace environment, pesticide and defoliants are also reviewed. Finally, control measures for environmental pollution including the role of government are reviewed.

155: Korean J Occup Environ Med. 1993 Sep;5(2):195-204. Korean.

A follow-up study on diatomaceous earth pneumoconiosis in the diatomite factory.

No abstract available.

156: Korean J Prev Med. 1993 Sep;26(3):371-386. Korean.

A study on diagnostic criteria of noise-induced hearing loss among workers in an iron foundry.

This study was carried out to evaluate diagnostic criteria of noise-induced hearing loss (NIHL) among workers in an iron foundry. Of 1,093 workers under the observation of noise-specific health examination, 184 workers were selected by way of first and second screening audiometric examination. A questionnaire survey, otological examinations, Rinne test and audiometric test were performed and the results were as follows; The degree of hearing impairment in the left ear was more severe than in the right ear($p<0.05$). The difference between hearing threshold of the first and the second hearing test at 1,000 Hz was about 5 dB with a narrow range of deviations while the difference at 4,000 Hz was about -7dB with a wide range. Of the total study workers, 84.8% were tested within 15 hours away from noise exposure, and the rest after 16 hours. This study has identified that mean hearing loss at 4,000 Hz showed a significant statistical difference among the two study groups while mean hearing loss by 4-divided classification did not. The same phenomena were observed between the group with and without tinnitus and between the group with and without difficulty in hearing($p<0.05$). Among 184 workers, 10 workers(5.4%) diagnosed as NIHL by old diagnostic criteria in contrast to 150 workers diagnosed as NIHL by the new diagnostic criteria. There was a significant difference between the two groups in the average hearing loss at 4,000 Hz and 4-divided classification($p<0.01$), but there were no significant difference in age, the duration of employment, blood pressure and the duration wearing the

personal hearing protector($p>0.05$). If we apply Early Loss Index(ELI) method, some workers in younger age group diagnosed as NIHL by the new diagnostic criteria were fallen into within the normal range. In the mean time older age group show reverse results in contrast to the above finding. It is too early to confirm the value of the usage of the new diagnostic criteria in hearing examination. Further study is called for to verify the value of this criteria.

157: Korean J Prev Med. 1993 Mar;26(1):147-164. Korean.

Development Of An On Site Diagnostic Tool To Detect Neuropsychiatric Impairment Due To Chronic Organic Solvent Exposure.

A study has been conducted on developing questionnaire to serve as on site diagnostic tools for the early detection of neuropsychiatric impairment among workers chronically exposed to low-level organic solvents. Two drafts of tentative questionnaire have been developed as follows ; several question items were selected from questionnaires which were administered to workers exposed to organic solvents in previous studies and were grouped into each symptom category based on the presence of its association using Guttman scaling method, then these selected items were reviewed by neuropsychiatry specialists. The final draft of the questionnaire (total symptom score=36) was developed by selecting 33 question items which had more than a 0.88 Guttman coefficient of reproducibility in each symptom category from a pilot study in which these tentative questionnaires were

administered to workers manufacturing soles. Three plants using organic solvents and one plant never using organic solvents as a control group were selected to test the reliability and validity of the developed questionnaires. The major organic solvent in the workplace environment detected by a personal air sampler and GC/MSD was toluene. The concentration of toluene in air from the department using organic solvent was statistically different from that of the department never using organic solvent. The concentration of toluene from almost all of the workplace did not exceed the allowable level. There was no statistically significant difference between the concentration of urinary hippuric acid from the workers of the department using organic solvent and that of the department never using it. Total symptom score of the plant never using organic solvents was 9.8 and those of the three plants using organic solvents were 15.6, 14.7, and 13.7 respectively. In order to evaluate the validity of the questionnaires, the workers from two different department of the plant in which usage of organic solvents are totally different were compared. The total symptom score was 17.8 for workers of the department using organic solvent and 13.5 for the department never using organic solvent and scores of each symptom group between exposure and non-exposure group also showed statistically significant difference. The finding that total symptom score of the usefulness of the developed questionnaire to assess the health effects of chronic exposure to organic solvents. The correlation coefficient, which was calculated to evaluate the test-retest reliability, was 0.581(p=0.001). The coefficient of Crohnbach which reflects the internal consistency of the questionnaire was 0.91. In conclusion, the reliability of the questionnaire was well maintained over the time lapse

between the two administrations of the questionnaire and despite the seasonal difference.

158: Korean J Occup Environ Med. 1993 Feb;5(1):152-162. Korean.

Blood carboxyhemoglobin levels and clinical symptoms of furnace-repairing workers.

No abstract available.

159: Korean J Occup Environ Med. 1993 Feb;5(1):104-113. Korean.

A study on the subjective symptoms of the workers exposed to chronic low dose organic solvents.

No abstract available.

160: Korean J Prev Med. 1992 Dec;25(4):343-356. Korean.

A Study on the Status of Seeking Intervention among the Workers with Health Problems Identified by the Workers' Periodic Health Examination.

Authors studied the workers' knowledge about the health problems detected

through the previous workers' periodic health examination, content of follow-up management and actions taken for their health problem detected on previous health examination. From June to September 1992, workers' periodic health examination was performed on workers employed in 10 companies located in 2 middle-sized Korean cities. A questionnaire survey was done for 150 workers who reported to have D2 result of either hypertension or liver disorder at the previous workers' periodic health examination done in 1991. The results are as follows; 1. Of 160 workers who had D2 result of either hypertension or liver disorder in previous examination one year before, only 85 workers(51.3%, 43 workers with hypertension, 38 workers with live disorder) responded that they have such disorders. The other 65 workers responded to questionnaire were all those with C results. Respondents' knowledge about their diagnoses was relatively precise (95.2% in hypertension group, 94.6% in liver disorder group) but knowledge about classification of diseases was poor. 2. The main efforts to solve the health problem was self management (26 spells, 55.3%), visiting clinic or hospital (6 spells, 12.8%), use of herb medicine (2 spells, 4.3%) and use of drug store (2 spells, 4.3%) in hypertension group. In liver disorder group, 30 spells (71.4%) relied on self management, 6 spells (14.3%) on hospital or clinic and 9 spells (21.4%) had no effort to improve the health problem. Content of self management was low salt diet, quit smoking, regular exercise and quit alcohol drinking in order. Avoidance of salt in diet was high in hypertension group and quitting alcohol drinking was high in liver disorder group. In those with self management, 80.7% of hypertension group and 83.3% of liver disorder group continued previous effort. Those, however, who utilized clinic or hospital, only 16.7% and

50.0% were still visiting hospital or clinic. 3. Fifty seven percent of hypertension group and 64.3% of liver disorder group was presently smoking, 8.5% and 11.9% reduced smoking and 21.3% and 14.3% stopped smoking. Forty nine percent of hypertension group and 28.6% of liver disorder group was presently drinking. Reduced alcohol intake was reported in 29.8% and 40.5%, 12.8% and 23.8% stopped alcohol drinking. Sixty six percent of hypertension group and 73.8% of liver disorder group did no regular exercise, but 12.8% and 11.9% of each group increased their physical exercise for last one year. Forty three percent of hypertension group and 38.1% of liver disorder group was overweight (defined by bodymass index greater or equal than 25). Reduced body weight was reported in 17.2% and 16.7% of each group. Reduced dietary salt intake was high in hypertension group (51.5%). The study results suggest that follow-up management after workers' periodic health examination is not satisfactory. In order to improve this situation, adequate information on the result of the workers' periodic health examination should be distributed to each worker group with health education and counselling.

161: Korean J Occup Environ Med. 1992 Oct;4(2):190-198. Korean.

A study on the status of management among workers diagnosed as hearing loss in an iron foundry.

No abstract available.

162: Korean J Epidemiol. 1992 Jun;14(1):59-69. Korean.

A study on the health status of the inhabitants exposed to cementdust.

No abstract available.

163: Korean J Occup Environ Med. 1992 Feb;4(1):61-69. Korean.

Development of diatomaceous earth pneumoconiosis in the diatomitefactory.

No abstract available.

164: J Korean Acad Fam Med. 1991 Oct;12(10):22-29. Korean.

A study on factors related with low visual acuity in elementaryschool children.

No abstract available.

165: J Korean Acad Fam Med. 1991 May;12(5):66-71. Korean.

A case report of a fatal mercury poisoning.

No abstract available.

166: Korean J Prev Med. 1991 Mar;24(1):86-92. Korean.

The Effect Of Gunshot Or Cannonade Training During Military Service On Hearing Threshold Levels.

To test if exposure history to rifle fire or cannonade training during military duty can induce hearing loss, history of personal military service and history of gunshot exposure were asked to 228 male college students with self-administrative questionnaire. Otoscopic examination and Rinne's test were performed if any abnormal finding was detected by pure-tone audiometry. Average hearing threshold levels of 500 Hz, 1,000 Hz, 2,000 Hz, 4,000 Hz and threshold levels at 4,000 Hz were calculated for 112 students who were remained after exclusion of cases with history of ear disease, of ototoxic drug administration, and of neuropsychiatric disease, and mean of those were compared between group of students who have completed military duty (completed group) and group of those who have not (not-completed group), and between group exposed(exposed group) and group unexposed to gunshot sound(unexposed group). Mean of average hearing threshold level and mean of threshold levels at 4,000 Hz of completed group and those of exposed group were higher than those of not-completed group and unexposed group, respectively. Proportion of cases that average threshold level was greater than 40 dB of threshold levels at 4,000 Hz was greater than 50 dB

were higher also in completed group and exposed group than in duty not-completed group and unexposed group, respectively. Multiple linear regression analysis including age, duration of military service, degree of gunshot sound exposure as independent variables and average hearing threshold level as dependant variable, was performed in order to estimate the effect of age on hearing, and any considerable effect of age on hearing could not be found. In conclusion, hearing impairment can be induced by rifle fire or cannonade training.

167: Korean J Prev Med. 1991 Mar;24(1):37-44. Korean.

Formaldehyde exposure in the plywood manufacturing factory.

In the plywood manufacturing factory established in 1979, female 3 workers who had exposed to adhesives containing formaldehyde for more than 48 months of duration suffered from eye, nasal, oral, throat and skin irritation and dyspnea as chief complaints. The actual level of the exposure to formaldehyde were not estimated when the exposed workers started to have above symptoms. The environmental monitoring of workplace was measured on April 25, 1990, and the concentration of formaldehyde revealed 0.2 ppm, however the exhaustive ventilatory system was already installed at that time. Twenty six subjects from entire factory were examined by questionnaire, physical examination and spirometry, etc. on August 22, 1990. Significant difference was observed in symptoms and signs of nasal, oral and throat irritation between teh exposed group with longer

duration and that with short duration. When the exposed group with longer duration was compared to the group of nonexposed, symptoms and signs of nasal, oral, throat and skin irritation, chest tightness and dullness were significantly increased in the former group. The results of the spirometric test showed that the forced vital capacity and the forced expiratory volume at 1 second were decreased amont the exposed group with longer duration but not significant.

168: Korean J Prev Med. 1982 Oct;15(1):205-212. Korean.

A Survey on the Damage done to the Farmers by Agrochemicals in a Rural Area of Korea.

This survey was conducted to find out demage done to the farmers by ;Agrochemicals in a rural area of Korea from January to October, 1980.
Choon Sung Gun, Kang Won Province was the survey area and the 412 males were surveyed among all those that have sprayed agrochemicals. during 10 Months in 1980.
Obtained results and findings from this survey are summarized as follows;
1. The total spray days of 413 males were 3,114 days and avarage spray days per person were 7.54 days. Also avarage spray hours per person were 4.7 hours. 2. The incidence rate per 100 persons of self-recognized skin manifestation was 12.6 persons and incidence rate per spray day was 2.7 percent. The incidence rate per 1Q0 persons of self-recognized intoxication was 23.0 persons and incidence rate per spray day was 3.6 percent. 3. In

cases where mask was not used, when it was syrayed in hot weather, when stronger solution was used, the results were higher percentage in self-recognized intoxication ($P<0.01$). 4. The symptoms of self-recognized intoxication were headache (55.8%), dizziness (46.9%),. nausea (17.7%), fatigue (17.0%), and vomiting (17.0;%). 5. Number of intoxication per 100 used standardized unit by agrochemicals was parathion (93.8 spells), sumithion (91.8 spells) and folithion (66.7 spells). 6. Treatment was done by health facility utilization (27 cases), visits to drug store (13 cases) and visits to health center (7 cases).

PubMed only articles (16)

1. Int J Environ Res Public Health. 2018 Jun 1;15(6). pii: E1143. doi: 10.3390/ijerph15061143.

Awareness and Work-Related Factors Associated with Scrub Typhus: A Case-Control Study from South Korea.

This study aimed to examine the awareness and the work-related factors associated with scrub typhus to provide data essential for evidence-based preventive strategies. A community-based case control study was carried out in the rural areas of Gyeongsangbuk-do, South Korea. Confirmed cases of scrub typhus (n = 57) were based on laboratory tests performed by the Korean Centers for Disease Control and Prevention (KCDC), 114 matched neighborhood controls, age (±6 years), gender and area of residence in the Gyeongsangbuk-do of South Korea. These cases

were contracted over the 12-month period of January to December 2015. Overall, 61.4% cases and 79.8% of the control group had heard about scrub typhus. Cases were less aware about the fact that mites are mainly found in the bushes and that long sleeves and full-length pants and boots helped prevent scrub typhus. However, more were aware of the eschar lesion as a characteristic sign of scrub typhus. Work related risk factors such as having a wetland or puddles of water surrounding the house, dry field farming and working in the livestock industry were significantly associated with the scrub typhus. Health promotion strategies, such as creating general awareness, personal protection methods and improving personal hygiene and environmental sanitation in collaboration with relevant sectors, are recommended to reduce the burden of scrub typhus. Further intervention studies on awareness and behavioral and environmental modifications are required to investigate the effectiveness of such interventions.

2. Environ Health Toxicol. 2018 Jan 16;33(1):e2018004. doi: 10.5620/eht.e2018004. eCollection 2018.

Health effects of environmental pollution in population living near industrial complex areas in Korea.

Several epidemiological studies have reported an association between environmental pollution and various health conditions in individuals residing in industrial complexes. To evaluate the effects of pollution from industrial complex on human health, we performed a pooled analysis of environmental epidemiologic monitoring data for residents living near national industrial

complexes in Korea. The respiratory and allergic symptoms and the prevalence of acute and chronic diseases, including cancer, were used as the outcome variables for health effects. Multiple logistic regression analysis was used to analyze the relationship between exposure to pollution from industrial complexes and health conditions. After adjusting for age, sex, smoking status, occupational exposure, level of education, and body mass index, the residents near the industrial complexes were found to have more respiratory symptoms, such as cough (odds ratio [OR], 1.18; 95% confidence interval [CI], 1.06 to 1.31) and sputum production (OR, 1.13; 95% CI, 1.03 to 1.24), and symptoms of atopic dermatitis (OR, 1.10; 95% CI, 1.01 to 1.20). Among residents of the industrial complexes, the prevalence of acute eye disorders was approximately 40% higher (OR, 1.39; 95% CI, 1.04 to 1.84) and the prevalence of lung and uterine cancer was 3.45 times and 1.88 times higher, respectively, than those among residents of the control area. This study showed that residents living in the vicinity of industrial complexes have a high risk of acute and chronic diseases including respiratory and allergic conditions. These results can be used as basic objective data for developing health management measures for individuals residing near industrial complexes.

3. Ind Health. 2016 Aug 5;54(4):370-6. doi: 10.2486/indhealth.2015-0159. Epub 2016 Mar 25.

The association among ferruginous body, uncoated fibers, asbestos and non-asbestos fibers in lung tissue in terms of length.

To demonstrate the correlations between the concentrations of ferruginous body as

well as uncoated fiber both of which can be observed with phase-contrast microscope and the concentration of various inorganic fibers including asbestos which requires the observation with TEM or SEM, we measured those indices among Japanese and Korean cases. Though the concentration of ferruginous body in lung tissue is an important index of asbestos exposure, uncoated fibers observed with phase-contrast microscope might be another index especially in such cases with relatively low exposure due to their history of living in a general environment. However, to establish the reliability of uncoated fibers as an index of asbestos exposure, analysis with more cases and from various backgrounds must be carried out.

4. PLoS One. 2016 Mar 18;11(3):e0151849. doi: 10.1371/journal.pone.0151849. eCollection 2016.

Humidifier Disinfectants Are a Cause of Lung Injury among Adults in South Korea: A Community-Based Case-Control Study.

BACKGROUNDS: An outbreak of lung injury among South Korean adults was examined in a hospital-based case-control study, and the suspected cause was exposure to humidifier disinfectant (HD). However, a case-control study with community-dwelling controls was needed to validate the previous study's findings, and to confirm the exposure-response relationship between HD and lung injury.
METHODS: Each case of lung injury was matched with four community-dwelling controls, according to age (±3 years), sex, residence, and history of childbirth since 2006 (for women). Environmental risk factors, which included type and use

of humidifier and HD, were investigated using a structured questionnaire during August 2011. The exposure to HD was calculated for both cases and controls, and the corresponding risks of lung injury were compared.
RESULTS: Among 28 eligible cases, 16 patients agreed to participate, and 60 matched controls were considered eligible for this study. The cases were more likely to have been exposed to HD (odds ratio: 116.1, 95% confidence interval: 6.5-2,063.7). All cases were exposed to HDs containing polyhexamethyleneguanidine phosphate, and the risk of lung injury increased with the cumulative exposure, duration of exposure, and exposure per day.
CONCLUSIONS: This study revealed a statistically significant exposure-response relationship between HD and lung injury. Therefore, continuous monitoring and stricter evaluation of environmental chemicals' safety should be conducted.

5. Am J Ind Med. 2015 Jul;58(7):764-72. doi: 10.1002/ajim.22463. Epub 2015 May 5.

Biomarkers for polycyclic aromatic hydrocarbons and serum liver enzymes.

BACKGROUND: Limited evidence suggests that human liver toxicity is associated with exposure to polycyclic aromatic hydrocarbons (PAHs).
METHODS: The association of urinary PAH metabolites with serum liver enzymes was tested among 288 workers at a petrochemical plant, using a general linear model (GLM) and multiple logistic regression.
RESULTS: Urine 2-naphthol levels were positively correlated with serum AST after adjustment for covariates in GLM. Comparing third tertile versus first tertile of

2-naphthol levels, the odds ratios (OR) were elevated for abnormal serum AST levels [OR = 4.1 (95%CI 1.6-10.2)] and abnormal serum ALT levels [OR = 2.4 (95%CI 1.2-4.9)].

CONCLUSIONS: Although confounding by alcohol intake was not completely ruled out, our findings demonstrate an association between PAHs exposure and elevation in serum liver enzymes. Urinary 2-naphthol is a biomarker of exposure to PAHs that is associated with liver toxicity.

6. Cien Saude Colet. 2014 Dec;19(12):4809-18.

A study of the status of exposure to polycyclic aromatic hydrocarbons (PAHs) in relation to its metabolites among workers in a Korean chemical factory.

The study was conducted to evaluate the status of worker exposure to polycyclic aromatic hydrocarbons (PAHs) through the measurement of urinary metabolites such as 1-hydroxypyrene (OHP) and 2-naphthol. A survey using a questionnaire involving 326 workers with measurement of urinary metabolites of 1-OHP and 2-naphthol was conducted. The differences in urinary 1-OHP and 2-naphthol concentrations, and changes in work, smoking habits and lifestyle were analyzed. The number of male subjects was 314 (96.3%), the largest age group was the fifth decade (170 cases, 52.1%). The urinary 1-OHP and 2-naphthol concentrations were significantly higher in the production workers. The urinary 1-OHP and 2-naphthol concentrations were significantly higher in smokers. In a multiple regression model, log (1-OHP) increased in smokers and production workers, while log (2-naphthol) only increased in smokers. Our results suggest that workers in this factory were

exposed to PAHs from non-occupational as well as occupational sources. The occupational exposure to PAHs can be reduced through the improvement of the process, but the exposure due to smoking can be prevented only by giving up smoking.

7. East Mediterr Health J. 2014 May 1;20(4):280-1.

Proposal for further study of risk factors and health policy for human brucellosis in Northern Jordan.

Comment on

East Mediterr Health J. 2013 Feb;19(2):135-40.

8. Vet Parasitol. 2014 Jun 16;203(1-2):29-34. doi: 10.1016/j.vetpar.2014.01.001. Epub 2014 Jan 11.

Cross-sectional analysis of the seropositivity and risk factors of Toxoplasma gondii infection among veterinarians, in relation to their public professional activities.

Toxoplasma gondii is a zoonotic pathogen that can infect most warm-blooded animals and humans. Although numerous epidemiological studies of T. gondii have been published, very limited data exist on the prevalence of T. gondii infection among veterinarians. In this study, a survey was used to analyze the

seropositivity of and risk factors for T. gondii infection among public veterinarians (PV) and veterinarians in veterinary service laboratories (VVSL). Blood samples were collected from individuals in both groups and examined by enzyme immunoassay (EIA). A questionnaire survey on work activities and personal dietary habits was also conducted. The positive rate for specific anti-T. gondii IgG antibody was 8% overall, comprising 13.4% in the PV group and 5.5% in the VVSL group. The seropositivity of T. gondii infection in the PV group was significantly related to involvement with the enforced destruction and contact with animals that were infected with zoonotic pathogens. Consumption of raw pork and drinking unboiled groundwater were also critical dietary risk factors for seropositivity for T. gondii infection. This is the first study of the factors that contribute to T. gondii infection among veterinarians with public professional activities in Korea. Further preventive educational programs for veterinarians with public professional activities are necessary to prevent exposure to T. gondii infection in this population.

9. Cien Saude Colet. 2013 Jun;18(6):1859-60.

Proposal of the global network for the study of green tobacco sickness.

10. Nephrology (Carlton). 2012 Mar;17(3):278-84. doi: 10.1111/j.1440-1797.2011.01552.x.

A simple prediction score for kidney disease in the Korean population.

AIM: Screening algorithms for chronic kidney disease have been developed and validated in American populations. Given the worldwide burden of kidney disease, developing algorithms for populations outside the USA is needed.
METHODS: Using simple, non-invasive questions, we developed a prediction model for chronic kidney disease from national population samples in Korea. The Korean National Health and Nutrition Examination Survey (n = 6565) was used for model development while validation was performed in two independent population samples, internal (n = 2921) and external datasets (n = 8166). Chronic kidney disease was defined as glomerular filtration rate < 60 mL/min per 1.73 m(2).
RESULTS: Seven factors - age, female gender, anaemia, hypertension, diabetes mellitus, cardiovascular disease and proteinuria - were significantly associated with prevalent chronic kidney disease. Integer scores were assigned to variables based on the magnitude of associations: 2 for age 50-59 years, 3 for age 60-69 years and 4 for age 70 years or older, and 1 for female gender, anaemia, hypertension, diabetes, proteinuria and cardiovascular dis ase. Based on the Youden index, a value of 4 or greater defined a high risk population with sensitivity 89%, specificity 71%, and positive predictive value 19%, and negative predictive value 99%. The area under the curve was 0.83 for the development set, and 0.87 and 0.78 in the two validation datasets.
CONCLUSION: This prediction algorithm, weighted towards common non-invasive variables, had good performance characteristics in an Asian population, and provides new evidence of the similarity of the algorithms for Western and Eastern populations.

11. Osong Public Health Res Perspect. 2011 Dec;2(3):198-201. doi: 10.1016/j.phrp.2011.11.045.

Prevalence of Farm and Slaughterhouse Workers Carrying Shiga Toxin-Producing Escherichia coli in Korea.

OBJECTIVES: The aim of this study was to investigate the distribution of Shiga toxin (Stx) gene-positive stool samples from dairy farmer and slaughterhouse workers in Gyeonggi-Do province.
METHODS: A total of 621 samples from healthy farmers and 198 samples from slaughterhouse workers were screened by polymerase chain reaction (PCR) for Shiga toxigenic Escherichia coli (STEC) infection on stool samples.
RESULTS: The PCR product of Stx-encoding genes was detected in 21 (3.4%) of 621 farmers and 15 (7.6%) of 198 slaughterhouse workers' stool samples. Distribution of the Stx PCR positive workers by age increment revealed an increase in STEC infection with age increment in both workers. Distribution of the Stx PCR positive workers by working years revealed an increase in STEC infection with working years in farmers.
CONCLUSION: These results of the study show that slaughterhouse workers are at higher risk of STEC infection than farmers. In addition, slaughterhouse workers have a more potential source of food contamination of STEC and transmission.

12. Scand J Infect Dis. 2012 Mar;44(3):168-73. doi: 10.3109/00365548.2011.631574. Epub 2011 Nov 10.

Anti-Shiga toxin immunoglobulin G antibodies in healthy South Korean slaughterhouse workers.

BACKGROUND: Slaughterhouse workers are in direct contact with cattle nearly every day. The purpose of this study was to survey the presence and distribution of anti-Shiga toxin 1 (Stx1) immunoglobulin G (IgG) in slaughterhouse workers, enabling a study of the serologic response to this toxin while working in an area at high-risk of Stx-producing Escherichia coli (STEC) infection.
METHODS: One thousand seven hundred and twenty-nine serum samples from healthy slaughterhouse employees were collected and surveyed by indirect enzyme-linked immunosorbent assay (ELISA).
RESULTS: Among the 5 slaughterhouse positions, slaughterers had the highest distribution of anti-Stx1 IgG values by an ELISA. Based on the ELISA values, 25% (433/1729) of the workers had anti-Stx1 IgG. Slaughterers, residual products handlers, inspectors, livestock hygiene controllers, and grading testers had anti-Stx1 IgG-positive rates of 28%, 25%, 20%, 19%, and 17%, respectively. The ELISA values of anti-Stx1 IgG increased with increases in the number of years worked by slaughterers, but not by residual products handlers, inspectors, livestock hygiene controllers, or grading testers.
CONCLUSIONS: From these results, slaughterhouse workers are healthy and asymptomatic; slaughterers in particular are at high-risk for STEC exposure.

13. Scand J Infect Dis. 2011 Apr;43(4):275-9. doi: 10.3109/00365548.2010.547990. Epub 2011 Jan 13.

Prevalence of Shiga toxin-encoding genes and risk factors among dairy farmers in Gyeonggi Province, Korea.

BACKGROUND: Dairy farmers perform various types of work and are in direct contact with dairy cattle nearly every day. The purpose of this study was to assess the prevalence of Shiga toxin-encoding genes (stx) among dairy farmers and to evaluate the relationship between stx and risk factors.
METHODS: A questionnaire developed in-house was sent to dairy farmers in Gyeonggi Province, Korea by registered mail. Researchers obtained stool samples and identified or administered the questionnaires by interview. The stool samples were examined for stx genes by polymerase chain reaction.
RESULTS: Twenty (3.4%) of 589 stool samples from dairy farmers were stx-positive. The distribution of stx-positive stool samples revealed an increase in Shiga toxin-producing Escherichia coli infection with age, duration of work, and herd size. There was no association between stx-positive stool samples and type of work. For artificial insemination, taking a shower after work was significant, and the proportion of stx-positive dairy farmers increased as taking a shower after work decreased.
CONCLUSIONS: Hygiene-related education to include taking a shower after sessions of artificial insemination should be considered. However, the stx-positive dairy farmers were small in number and the results should be interpreted with caution.

14. Emerg Infect Dis. 2009 Jul;15(7):1127-9. doi: 10.3201/eid1507.080399.

Rapid increase of scrub typhus, South Korea, 2001-2006.

15. FEMS Immunol Med Microbiol. 2009 Jun;56(1):41-7. doi:
10.1111/j.1574-695X.2009.00545.x. Epub 2009 Mar 20.

Asymptomatic healthy slaughterhouse workers in South Korea carrying Shiga toxin-producing Escherichia coli.

A total of 1602 stool samples from healthy employees in a slaughter company were screened by PCR for Shiga toxin (Stx)-producing Escherichia coli (STEC). The PCR product of Stx-encoding genes was detected in 90 (5.6%) of 1602 stool samples. Among the 90 stx-positive workers, the Residual Products Handlers and Slaughterers had rates of 8.0% and 6.0%--higher than Inspectors, Grading Testers and Livestock Hygiene Controllers at 3.3%, 2.0% and 3.5%, respectively.
Forty-nine (54.4%) were shown to have stx2; 25 (27.7%) carried stx1 and 16 (17.7%) had both stx1 and 2. Distribution of the stx PCR-positive workers by age revealed an increase in STEC infection with age ($P<0.05$). Phenotypic and genotypic traits of nine STEC strains isolated from eight slaughter plant workers were characterized. A variety of serotypes, five O serogroups (O8, O54, O59, O103 and O153) and two H serogroups (H7 and H32) were found, but none of the strains belonged to the serogroup O157. Eight Vero cell cytotoxicity assay-positive strains were isolated from the workers and these workers were asymptomatic and healthy. The results of the study show that slaughter plant workers are at high risk of STEC infection.

16. Am J Trop Med Hyg. 2009 Mar;80(3):442-6.

A community-based case-control study of behavioral factors associated with scrub typhus during the autumn epidemic season in South Korea.

A community-based, case-control study was carried out to investigate risk factors for scrub typhus in South Korea. Cases (n = 299) were defined as persons who were diagnosed serologically within the past two weeks. Two neighborhood control subjects were selected by matching for sex, age, and occupation. Taking a rest directly on the grass, working in short sleeves, working with bare hands, and squatting to defecate or urinate posed the highest risks, with adjusted odds ratios (aORs) and 95% confidence intervals (CIs) of 1.7 (1.2-2.3), 1.6 (1.1-2.4), 1.7 (1.2-2.4), and 2.0 (1.4-2.9), respectively. Wearing a long-sleeved shirt while working, keeping work clothes off the grass, and always using a mat to rest outdoors showed protective associations, with aORs and 95% CIs of 0.5 (0.3-0.9), 0.6 (0.4-0.9), and 0.7 (0.5-0.9), respectively. These results might be useful in the establishment of a detailed control strategy for scrub typhus.

17. J Occup Health. 2008;50(6):525-8. Epub 2008 Oct 23.

Prevalence of chronic widespread pain and chronic fatigue syndrome in Korean livestock raisers.

18. Ind Health. 2008 Oct;46(5):448-54.

Occupational infectious diseases among Korean health care workers compensated with Industrial Accident Compensation Insurance from 1998 to 2004.

Using the database of the Korea Labor Welfare Corporation (KLWC), which is the public organization for workers' compensation in Korea, we analyzed the occupational infectious diseases among the health care workers who were compensated by the Industrial Accident Compensation Insurance (IACI). From January 1998 to December 2004, 307 cases of infectious diseases were approved as being cases of occupational diseases. Women accounted for 83% (254 cases) of the compensated cases. The most common age group was 20-29 yr of age (228 cases, 74.3%). The majority of infections were tuberculosis (203 cases, 66.1%), hepatitis (42 cases, 13.7%), chickenpox (11 cases, 3.6%), AIDS (8 cases, 2.6%) and scabies (7 cases, 2.3%). The major types of occupations were nurses, including 18 aid-nurses (223 cases, 72.6%), doctors (37 cases, 12.1%), clinical pathology technicians (18 cases, 5.9%) and workers who were taking care of patients (8 cases, 2.6%). The mean working duration after infection was 3.9 yr and the mean duration of recuperation was 9.7 months. The most common department where the infected workers were working was the inpatient ward (161 cases, 52.4%), followed by the intensive care unit (51 cases, 16.6%). Through this study, we were able to elucidate the characteristics of occupational infectious diseases among Korean health care workers. These results have to be considered when establishing the management policy for prevention of occupational infectious diseases among Korean health care workers. Also, all knowledge from these Korean cases will be helpful to make good practices to promote occupational safety and health in the new era of globalization.

19. Ind Health. 2005 Oct;43(4):647-55.

Comparison of disability duration of lumbar intervertebral disc disorders among types of insurance in Korea.

The incidence of work-related musculoskeletal disorder including low back pain sharply increased since 2000 in Korea. The objectives of the present study are to compare disability duration of lumbar intervertebral disc displacement among types of insurances, and to obtain its appropriate duration. The medical records of all patients whose final diagnosis in discharge summary of chart was lumber specified intervertebral disc displacement (LIVD) in 6 large general hospitals in Korea were reviewed to compare the length of admission and disability among different types of insurances. The information on age, gender, the length of admission, the length of follow-up for LIVD, occupation, operation, combined musculoskeletal diseases, and type of insurance was investigated. 552 cases were selected and analyzed to calculate arithmetic mean, median, mode, and geometric mean of disability duration. There was a significant difference in the length of admission and disability among types of insurance after controlling covariates such as age and combined diseases by the analysis of covariance. The length of admission in cases of IACI and AI was much longer than that of HI, and the length of disability in cases of IACI was much longer than that of HI. Prolonged duration of admission and disability was not assumed due to combined diseases, complication or other unexplainable personal factors in cases of those with industrial accidents compensation insurance and automobile insurance. This means

that proper management of evidence-based disability duration guidelines is urgently needed in Korea.

20. Ind Health. 2004 Apr;42(2):171-8.

Airborne asbestos and non-asbestos fiber concentrations in non-occupational environments in Korea.

Both airborne asbestos and non-asbestos fiber concentrations were evaluated in Korean non-occupational environments. The airborne fiber concentrations were analyzed in 96 air samples, from 48 different points, by transmission electron microscopy, with energy-dispersive X-ray analysis. The geometric means of the airborne asbestos and non-asbestos fiber concentrations were 0.62 and 67.86, and 0.30 and 17.47 fibers/liter in urban and rural areas, respectively. There were significant differences in both the airborne asbestos and non-asbestos fiber concentrations between the urban and rural areas ($p<0.05$). The geometric means of airborne asbestos and non-asbestos fiber concentrations were 0.67 and 37.93, and 0.27 and 30.67 fibers/liter at the points less than 10 m and more than 30 m away from highways, respectively. The airborne asbestos concentrations were significantly higher at the points less than 10 m away than at the points more than 30 m away from highways ($p<0.01$).

21. Ind Health. 2004 Apr;42(2):163-70.

Pulmonary asbestos and non-asbestos fiber concentrations in autopsied inhabitants in Pohang, Korea.

To establish reference values for pulmonary asbestos and non-asbestos fiber concentrations in rural Korean residents, and their comparison with those of urban Korean residents and the Japanese, autopsied lung samples from 22 subjects (20 males and 2 females), in Pohang, without known occupational asbestos exposure histories, were analyzed for asbestos and non-asbestos fibers, using transmission electron microscope equipped with an energy dispersive X-ray analyzer. Chrysotile was the major fiber type found in the lungs of the subjects. The residents in Pohang had significantly lower asbestos and non-asbestos fiber concentrations than the Korean urban residents. The Koreans had significantly lower asbestos and non-asbestos fiber concentrations than the Japanese.

22. Ind Health. 2003 Jul;41(3):149-57.

Impact of Agent Orange exposure among Korean Vietnam veterans.

In order to determine whether Agent Orange exposure was associated with increased frequency of medical problems, we conducted a cross-sectional epidemiologic study of Korean veterans during 1995-1996. 1,224 Vietnam and 154 non-Vietnam veterans were included in the study. Exposure to Agent Orange was assessed by structured in-depth interview on the participants' history of service in Vietnam. Health outcomes were assessed by a standardized comprehensive clinical investigation by a group of clinical specialists. The differences in the prevalence of various

medical diagnoses were assessed by Cochran-Mantel-Haenszel chi-square tests comparing the exposure levels of Vietnam veterans, adjusting for age. Multiple logistic regression was performed to estimate the effect of "service in Vietnam" adjusting for age, smoking, alcohol, body mass index, education, and marital status. Vietnam veterans had an increased frequency of eczema (odds ratio [OR] = 6.54), radiculopathy (OR = 3.98), diabetes (OR = 2.69), peripheral neuropathy (OR = 2.39), and hypertension (OR = 2.29), compared to non-Vietnam veterans, adjusting for potential confounders. In addition, higher levels of exposure among Vietnam veterans were associated with increased frequency of ischemic heart disease ($p < 0.01$), valvular heart disease ($p < 0.01$), and retinopathy ($p < 0.01$). We conclude that exposure to Agent Orange is associated with various health impacts in Korean Vietnam veterans.
